# A Novel Terminal-Repeat Retrotransposon in Miniature (TRIM) Is Massively Expressed in *Echinococcus multilocularis* Stem Cells

**DOI:** 10.1093/gbe/evv126

**Published:** 2015-07-01

**Authors:** Uriel Koziol, Santiago Radio, Pablo Smircich, Magdalena Zarowiecki, Cecilia Fernández, Klaus Brehm

**Affiliations:** ^1^Institute of Hygiene and Microbiology, University of Würzburg, Germany; ^2^Sección Bioquímica y Biología Molecular, Facultad de Ciencias, Universidad de la República, Montevideo, Uruguay; ^3^Laboratorio de Interacciones Moleculares, Facultad de Ciencias, Universidad de la República, Montevideo, Uruguay; ^4^Departamento de Genética, Facultad de Medicina, Universidad de la República, Montevideo, Uruguay; ^5^Parasite Genomics, Wellcome Trust Sanger Institute, Wellcome Trust Genome Campus, Hinxton, Cambridge, United Kingdom; ^6^Cátedra de Inmunología, Facultad de Química, Universidad de la República, Montevideo, Uruguay

**Keywords:** retrotransposition, long noncoding RNA, nonautonomous, neoblast, pluripotency

## Abstract

Taeniid cestodes (including the human parasites *Echinococcus* spp. and *Taenia solium*) have very few mobile genetic elements (MGEs) in their genome, despite lacking a canonical PIWI pathway. The MGEs of these parasites are virtually unexplored, and nothing is known about their expression and silencing. In this work, we report the discovery of a novel family of small nonautonomous long terminal repeat retrotransposons (also known as terminal-repeat retrotransposons in miniature, TRIMs) which we have named *ta-TRIM* (taeniid TRIM). *ta-TRIM*s are only the second family of TRIM elements discovered in animals, and are likely the result of convergent reductive evolution in different taxonomic groups. These elements originated at the base of the taeniid tree and have expanded during taeniid diversification, including after the divergence of closely related species such as *Echinococcus multilocularis* and *Echinococcus granulosus*. They are massively expressed in larval stages, from a small proportion of full-length copies and from isolated terminal repeats that show transcriptional read-through into downstream regions, generating novel noncoding RNAs and transcriptional fusions to coding genes. In *E. multilocularis*, *ta-TRIM*s are specifically expressed in the germinative cells (the somatic stem cells) during asexual reproduction of metacestode larvae. This would provide a developmental mechanism for insertion of *ta-TRIM*s into cells that will eventually generate the adult germ line. Future studies of active and inactive *ta-TRIM* elements could give the first clues on MGE silencing mechanisms in cestodes.

## Introduction

Mobile genetic elements (MGEs) have the capacity to replicate within the genome of their host. This gives them a selective advantage over other genetic elements, resulting in their ability to increase their frequency in natural populations (Kidwell and Lisch 2001; Werren 2011). In this sense, they act as “selfish” genetic elements, making a sizeable portion of the genomes of most eukaryotes (Wicker et al. 2007; Werren 2011). However, like all genetic elements they are a substrate for natural selection and can be an important source of variation and novelty for the evolution of genomes (Kidwell and Lisch 2001; Werren 2011).

Long terminal repeat (LTR) retrotransposons are a large group of MGEs present in almost all eukaryotes, including animals (metazoans) (Boeke and Stoye 1997; Havecker et al. 2004; Wicker et al. 2007). These elements consist of two identical direct repeats (LTRs) that flank sequences coding for structural components (Gag) and enzymatic components (protease [PR], reverse transcriptase [RT], RNAse H [RH], and integrase [IN]), which are required for the retrotransposition cycle. PR, RT, RH, and IN are encoded in a polyprotein (Pol) that is later processed into individual polypeptides by PR. The 5′-LTR functions as an RNApol II promoter for the transcription of the retrotransposon RNA. Although it has an identical sequence, the 3′-LTR is instead the site of 3′-end processing of the retrotransposon transcript, which is cleaved and polyadenylated by the cellular machinery (Klaver and Berkhout 1994; Schrom et al. 2013). The retrotransposon RNA, which therefore lacks the 5′ most region of the 5′-LTR (U3 region) and the 3′ most region of the 3′-LTR (U5 region), is exported to the cytoplasm where it is translated. In the cytoplasm, the retrotransposon RNA associates with Gag to form viral-like particles (VLPs) where reverse transcription takes place (Telesnitsky and Goff 1997; Havecker et al. 2004). Reverse transcription is a complex mechanism that results in the formation of double-stranded retrotransposon cDNA with complete LTRs (Telesnitsky and Goff 1997). For reverse transcription, two other sequence elements are crucial. The first is the primer binding site (PBS), located immediately downstream of the 5′-LTR, where binding of a tRNA to a sequence of 8–18 bases complementary to its 3′-end serves as the primer for the synthesis of the (−) retrotransposon cDNA strand (Mak and Kleiman 1997). The other is the polypurine tract (PPT), located immediately upstream of the 3′-LTR, from which the synthesis of the (+) retrotransposon cDNA strand begins (Telesnitsky and Goff 1997). The retrotransposon cDNA is imported into the nucleus, where it is integrated into the genome by IN. For this, IN recognizes sequences at the ends of the LTRs (around 8–20 bases), of which the most crucial part are the invariable 5′-CA-3′-OH ends (Hindmarsh and Leis 1999; Zhou et al. 2001). IN catalyzes the joining of the ends of the retrotransposon cDNA to a staggered double-stranded cut in the genomic DNA. The single stranded gaps between the retrotransposon cDNA and the genomic DNA are repaired by the cell, resulting in the duplication of the cut target sequence as direct repeats flanking the integrated retrotransposon (target site duplications; TSD) (Hindmarsh and Leis 1999; Ballandras-Colas et al. 2013).

In addition to these functional retrotransposons, other elements have been described that lack most or all of the coding sequences, but which are still mobilized by the machinery of functional (autonomous) elements (Havecker et al. 2004; Wicker et al. 2007; Schulman 2012). These nonautonomous elements only have the noncoding sequences that are essential for the retrotransposition cycle, such as the LTRs, PBS, and PPT. Nonautonomous elements include large retrotransposon derivatives (LARDs, >4 kb) and terminal-repeat retrotransposons in miniature (TRIMs, <4 kb, typically around 0.5–1 kb in length) (Witte et al. 2001; Jiang et al. 2002; Havecker et al. 2004; Wicker et al. 2007; Schulman 2012). LARDs and TRIMs were originally described in plants (Witte et al. 2001; Jiang et al. 2002; Kalendar et al. 2008), and only recently was the first example of a group of TRIMs described in a metazoan (the red harvester ant, *Pogonomyrmex barbatus*; Zhou and Cahan 2012). TRIMs of plants and ants are presumed to be the result of convergent reductive evolution from autonomous elements, retaining only the minimal sequences required to efficiently complete the retrotransposition cycle (Schulman 2012).

Because of the deleterious effect of MGEs, the hosts have developed effective mechanisms to suppress their expression and expansion. This is particularly important in the germ line of animals, as replication of MGEs in these cells would lead to their accumulation in the next generation. The PIWI pathway is a conserved metazoan mechanism that silences MGEs in the germ line (Juliano et al. 2011). PIWI is a member of the Argonaute family of proteins, which are involved in gene silencing through small RNAs (Hock and Meister 2008). PIWI proteins are associated with a specific class of small RNAs (PIWI-associated RNAs, or piRNAs), and the PIWI/piRNA complex can silence the MGEs at the epigenetic level and by posttranscriptional regulation of RNA stability (Juliano et al. 2011).

Many invertebrates have a discontinuous germ line, which is generated from multipotent stem cells after embryonic development (Extavour and Akam 2003; Juliano et al. 2010). In planarians and other free-living flatworms (phylum Platyhelminthes), these adult multipotent somatic stem cells are denominated neoblasts, and are the cellular basis for their regenerative capabilities, including the ability for de novo formation of the germ line (Sato et al. 2006; Juliano et al. 2011; Rink 2013). Free-living flatworms have been shown to specifically express *piwi* homologs in their neoblasts (Reddien et al. 2005; Palakodeti et al. 2008), and it has been suggested that these *piwi* genes could play a role in the protection of the genome of neoblasts from MGEs (Rink 2013).

In contrast, *piwi* orthologs have been lost from the main group of parasitic flatworms (the Neodermata, including the flukes [Digenea] and tapeworms [Cestoda]) (Tsai et al. 2013; Skinner et al. 2014). In the digenean *Schistosoma mansoni*, a member of a divergent group of neodermatan-specific *argonaute *genes (*sm-ago2-1*) is highly expressed in neoblasts, and it has been proposed that this gene could perform similar functions to those of *piwi* in other organisms (Collins et al. 2013; Wang et al. 2013). We have recently characterized the neoblast-like stem cells of the asexually proliferating larva of the cestode *Echinococcus multilocularis *(Koziol et al. 2014). In cestodes, such cells are typically denominated “germinative cells” (Reuter and Kreshchenko 2004). In contrast to *S. mansoni*, only a fraction of the *E. multilocularis* germinative cells express *em-ago2-A-C* (orthologs of *sm-ago2-1*), and expression is also seen in postmitotic, differentiated cells, so it is unclear whether the functions of these *argonaute* genes are shared between cestodes and trematodes (Koziol et al. 2014). However, the burden of MGEs in *E. multilocularis* is very low (∼2% of the genome), as is for other related cestodes, indicating that a highly effective mechanism of MGE suppression may be at play (Tsai et al. 2013; Skinner et al. 2014).

In this work, we report the discovery of a novel group of TRIM elements (taeniid TRIMs [*ta-TRIM*s]) that is specific for *E. multilocularis* and other related taeniid cestodes. A fraction of these TRIMs have escaped silencing and are massively expressed in the *E. multilocularis* germinative cells, constituting their best molecular marker to date. These TRIMs have expanded during the evolution and divergence of taeniids, and may still be mobilizing in some species. In *E. multilocularis*, these elements may now be inactive for retrotransposition, but have left in their wake a substantial reshaping of the host’s transcriptome, as they are the source of TRIM transcripts and of fusion transcripts to coding genes and novel noncoding RNAs.

## Materials and Methods

### Expressed Sequence Tags and Genomic Assemblies

Genomic assemblies from *E. multilocularis*, *Echinococcus granulosus*, *Taenia solium* and *Hymenolepis microstoma* (Tsai et al. 2013) as well as *S. mansoni* (Berriman et al. 2009) were downloaded from the GeneDB database at the Wellcome Trust Sanger Institute (genedb.org). Draft assemblies of *Taenia asiatica*, *Taenia taeniaeformis*, *Mesocestoides corti,* and *Diphyllobothrium latum* were generated by the Parasite genomics group of the Wellcome Trust Sanger Institute in the context of the 50 helminth genomes initiative, and are available at ftp://ftp.sanger.ac.uk/pub/project/pathogens/HGI (last accessed July 13, 2015). The draft genome of the planarian *Schmidtea mediterranea* was obtained from SmedGD (smedgd.neuro.utah.edu) (Robb et al. 2008). Expressed sequence tags (ESTs) from *T. solium* were downloaded from GenBank, and ESTs from *Echinococcus* spp. were collected from GenBank, GeneDB, and the *Echinococcus* Full-Length cDNA project (http://fullmal.hgc.jp/index_em_ajax.html, last accessed July 13, 2015).

### Search for *ta-TRIM* ESTs and Genomic Loci

Initially, we performed BLASTN searches of *Echinococcus* spp. ESTs and genomes using as a query the previously described Cluster A of long noncoding RNAs (lncRNAs) of *E. granulosus *(Parkinson et al. 2012). Once the loci were recognized due to their characteristics as possible TRIMs, full-length *ta-TRIM* elements from *E. multilocularis* were used for BLASTN searches in the *E. multilocularis* genome, as well as in other cestodes and flatworms. For *E. multilocularis*, we also constructed hidden Markov models (HMM) from full length sequences using hmmer2.3 (www.hmmer.org), and used them to search for additional *ta-TRIM* elements. Because of the divergence between *ta-TRIM* sequences in different taeniid species, the manually identified *ta-TRIM* sequences from each species were used for new BLASTN searches. A list of full-length elements was extracted and manually curated for each species from these BLASTN results, which was used for analysis of TSD sites, LTR divergence, and comparison of synteny between species (supplementary data S14, Supplementary Material online). A separate list containing all fragments longer than 800 bp from each species was compiled for phylogenetic analyses.

In order to indentify *ta-TRIM*s that are transcribed, we identified ESTs of *E. multilocularis* with similarity to *ta-TRIMs* (as determined by BLASTN with an expect value threshold of e-10) and mapped them to the *E. multilocularis* genome by BLASTN, eliminating all hits with less than 99% identity to the genome or smaller than 50 bp, as well as those which mapped to more than one region in the genome with greater than 99% identity. The resulting loci, plus a duplicated genomic locus which was supported from 3′ rapid amplification of cDNA ends (RACE) experiments, were collected and manually analyzed to determine whether they were full-length *ta-TRIMS* or solo-LTRs, whether transcription was likely initiated from within an LTR, and whether there was downstream transcriptional read-through into neighboring intergenic regions or coding genes. An identical analysis was performed for *E. granulosus*, except that the identity threshold was decreased to 95%, due to the draft quality of the assembly.

### Identification of the *ta-TRIM* PBS

A list of *E. multilocularis* tRNA genes was generated from the genomic assembly with tRNAscan-SE (Lowe and Eddy 1997). The 3′ region of these tRNAs was compared with the region immediately downstream of the 5′-LTR. Only a family of ^Leu^tRNA genes was identified as having complementarity for eight or more bases in this region.

### Search for Autonomous LTR Retrotransposons

We searched for autonomous LTR retrotransposons in *E. multilocularis* by TBLASTN using as queries the sequences of Pol proteins of LTR retrotransposons from *Drosophila melanogaster* and *S. mansoni* (Laha et al. 2001; DeMarco et al. 2004; Laha et al. 2004). Although we could not find any intact Pol proteins, among the sequences with the longest Pol fragments we identified partial copies of a novel LTR-retrotransposon (*lennie* elements). The LTRs, PBS, and PPT were manually identified from these sequences.

### High Throughput RNA Sequencing Analysis

In order to determine the transcriptional activity of *ta-TRIM*s, RNA sequencing (RNA-Seq) data sets from Tsai et al. (2013) were mapped to the reference genomes using Tophat version 2.0.6 (Trapnell et al. 2012). Parameters used were: -r 300–mate-std-dev 100 -i 10 -I 40000 -g 40 -a 4, set using prior knowledge about the genome and RNA-Seq libraries. Reads per element was calculated using featureCount of the Rsubreads package (Liao et al. 2014), after the removal of duplicate reads, multimapping reads, and reads with a mapping quality of less than 30 using custom scripts and SAMtools v.0.1.19+ (Li et al. 2009). Heatmaps were constructed with the “heatmap.2” R package. The “hclust” function was used for hierarchical clustering and the “dist” function was used for distance matrix calculations using default parameters.

### Phylogenetic Analysis

An alignment of full-length *ta-TRIM* elements and fragments longer than 800 bases from all species was performed with ClustalW (Thompson et al. 1994), and the region corresponding to the 5′-LTR was removed (as it is not independent from the 3′-LTR sequence). Maximum-likelihood phylogenetic analysis was performed using MEGA 5.0 (Tamura et al. 2011), under a Kimura 2-parameter model with gamma distributed sites (K2P+G, gamma parameter = 1), which was the model that gave the best fit to the data using the “find best DNA/protein models (ML)” feature.

### Comparison of *ta-TRIM* Loci between Taeniid Species

When comparing *ta-TRIM* loci for evidence of integration, each locus with a full-length *ta-TRIM* of *E. multilocularis* was blasted together with 2 kb of upstream and downstream flanking sequence to the *E. granulosus* genome. BLAST (Basic Local Alignment Search Tool) hits with less than 80% identity upstream or downstream of the *ta-TRIM* were discarded, and the retrieved sequences were aligned with ClustalW and manually inspected. The same procedure was performed when comparing *T. solium* and *T. asiatica*.

### Search for Gene Conversion

Alignments of *ta-TRIM*s for each taeniid species were analyzed using the program GENECONV (Sawyer 1999), which looks for statistically significant tracts of identity between two sequences, given their overall divergence. Global *P* values (i.e., corrected for multiple comparisons as described by Sawyer 1999) were considered significant when the value was below 0.05.

### Estimates of Substitution Rates from Introns and Divergence between LTRs

First, we estimated the neutral rate of substitution based on the alignment of intronic sequences from *Echinococcus vogeli* and *Echinococcus oligarthrus*. Introns from the *elp* and *pold* genes from *E. vogeli* and *E. oligarthrus* (Knapp et al. 2011) were retrieved from GenBank and aligned using ClustalW. The first 10 and the last 30 bases of each alignment were discarded (to remove functional splice sites; Hoffman and Birney 2007) and the substitutions per site (transitions and transversions only) under a K2P+G model were estimated using MEGA 5.0 (Tamura et al. 2011). The intron neutral substitution rate was estimated from the average of K2P+G estimates of both genes using the formula r = s/2t, where r is the substitution rate, s the estimated substitutions per site, and t the time of divergence (set as 3 Myr for *E. vogeli* and *E. oligarthrus*; Knapp et al. 2011). For *E. multilocularis*, *E. granulosus*, and *T. solium*, divergence values were also estimated for other well-characterized genes (*emmpk2*, *emsmadC*, *emmpk1*, *emraf*, *emegfr*, and *emir1* from *E. multilocularis *[Spiliotis et al. 2003, 2005, 2006; Gelmedin et al. 2008; Zavala-Gongora et al. 2008; Hemer et al. 2014], *egpum1* from *E. granulosus *[Koziol et al. 2008], and their orthologs retrieved from the other species). When comparing *T. solium* and *E. multilocularis*, only the shortest introns showing trustworthy alignments were included, and therefore the divergence values between both species are conservative.

For determining the divergence between LTRs of individual *ta-TRIM*s, the 5′-LTR and 3′-LTR were aligned from individual full-length *ta-TRIM* elements from each species. The actual per site divergence, as well as the total estimated substitutions per site (transitions and transversions only) under a K2P+G model, was calculated using MEGA 5.0 (Tamura et al. 2011).

### 3′-RACE and Reverse Transcription—Polymerase Chain Reaction

Reverse transcription was performed with 700 ng of total RNA from in vitro cultured *E. multilocularis* larvae (Spiliotis and Brehm 2009) using Prime-Script RT (Takara) as instructed by the manufacturer, with the primer AAGCAGTGGTATCAACGCAGAGTAC-T_30_-VN. Reverse transcription—polymerase chain reactions (RT-PCRs) were performed with 2 μl of cDNA per reaction using KOD polymerase (Millipore). For 3′-RACE of *ta-TRIM*s, a seminested RT-PCR approach was performed, using degenerate forward nested primers for the U5 region of several *ta-TRIM*s (TGTGTCTTCTTTCGTNTTCAGGGAG and TCAGGGAGTCYYGGGAYGCTACA for the first and second PCR reactions, respectively) and the reverse primer AAGCAGTGGTATCAACGCAGAGTAC. For confirming the transcriptional fusions between solo-LTRs and downstream coding genes, the following primer combinations were used: locus 8, TTCGTCTTCTTTCGTCTTCAGAGAG and GCATCCTTGATCGAAGTTTGGG (fusion to EmuJ_000118100); locus 21, CTTTTGTACTTTGAGTTAGCCCCTTGTAC and CCATGGCGAAATCGACCAC (fusion to EmuJ_000465100); locus 60, CCTTGTACCTAGCTAAGAGGGCTGAC and CGACGTAGGCACTCAAGCAAG (fusion to EmuJ_001025350); locus 2 (the only one for which RT-PCR was unsuccessful, due to unspecific amplification from another *ta-TRIM* element), CCGAGTATTGTGTCTTCTTTCGTCTTC and CGGAATGACATTTGGCAAAGTC (fusion to EmuJ_000054900). All products of the expected size were gel-purified, cloned into pJET1.2 (Thermo-Scientific), and several clones were sequenced for each product.

### Whole-Mount In Situ Hybridization

Fluorescent whole-mount in situ hybridization (WMISH) of in vitro cultured *E. multilocularis* larvae was performed with a digoxigenin-labeled antisense probe, corresponding to the region interior to the LTRs of the *ta-TRIM* locus 39 of *E. multilocularis* (supplementary data S8, Supplementary Material online), as described (Koziol et al. 2014). Primers TTGGTGGCAGCGGAAAGC and CCTCTTTTGAGTGTTATCCCCAGC were used to amplify the probe region from genomic DNA of *E. multilocularis*, and the product was cloned into pJET1.2 (Thermo-Scientific). Three independent experiments with different laboratory isolates were performed. These isolates were GH09 and Ingrid/10, obtained from accidental infections of Old World Monkeys in a breeding exclosure (Tappe et al. 2007), and MS10/10, obtained from an infected dog. All isolates had been kept in the laboratory by serial peritoneal injection into *Meriones unguiculatus* for 4 years or less. Control WMISH experiments using the corresponding sense probe were always negative (data not shown). In vitro 5-Ethynyl-2′-deoxyuridine labeling (EdU; 50 μM for 5 h) and detection were performed as previously described (Koziol et al. 2014).

## Results

### Discovery of TRIM Elements in Taeniid Cestodes

**Fig. 1.—:**
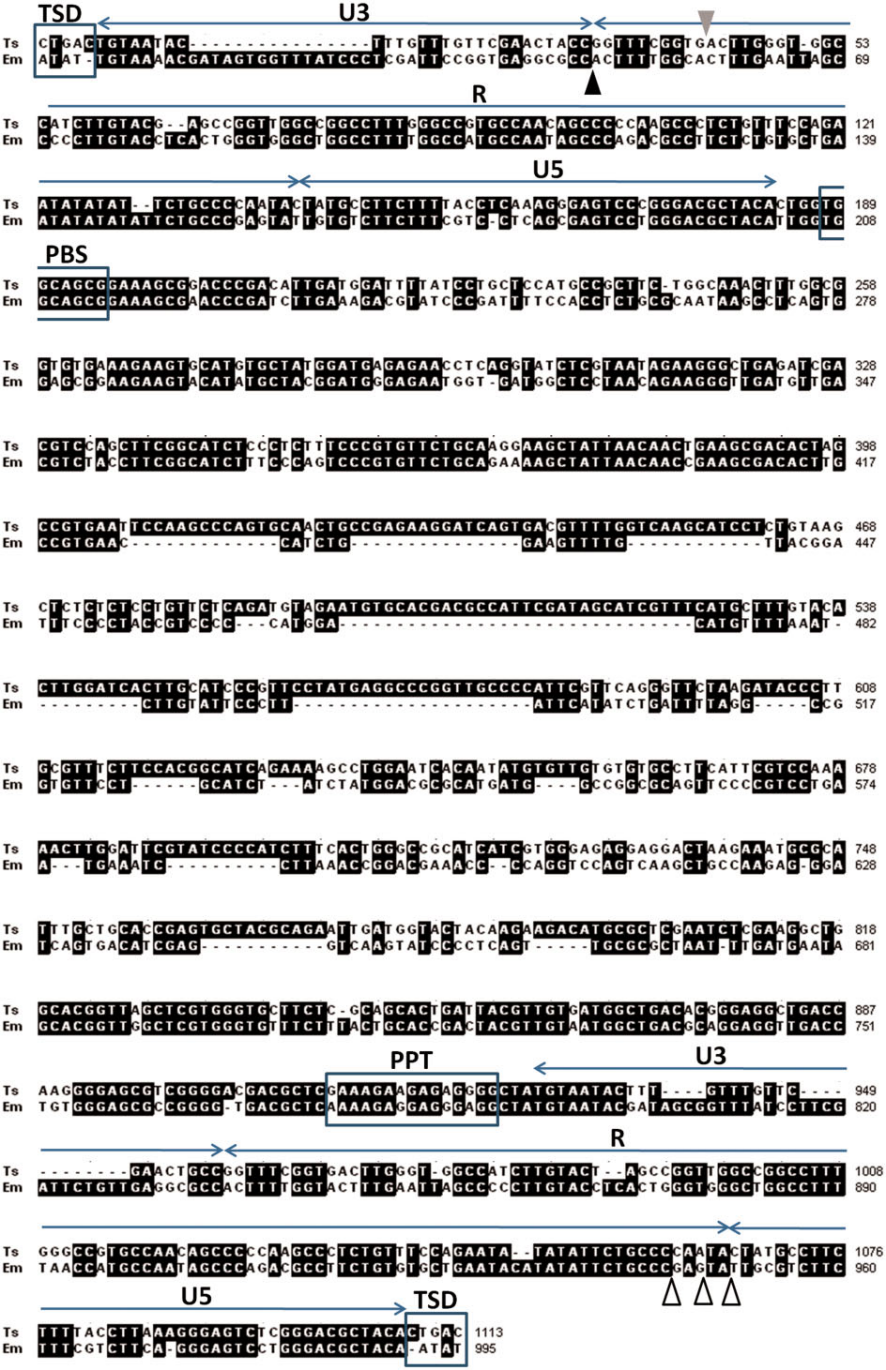
Alignment of *ta-TRIM*s from *E. multilocularis* (Em) and *T. solium* (Ts). The limits of the U3, R, and U5 regions of the LTRs are indicated above for Em. The arrowheads in the 5′-LTR show the beginning of transcription in Em elements (black), and Ts (gray), as determined from full length ESTs. White arrowheads in the 3′-LTR show the 3′-end of *ta-TRIM* transcripts in Em, as determined from full length ESTs and 3′-RACE experiments.

In *Echinococcus* spp., the metacestode larvae develop as fluid-filled cysts in which numerous protoscoleces, the infective form for the definitive host, develop from the cyst wall by asexual budding. Previously, an lncRNA of approximately 900 bases with similarity to the *E. granulosus* repeat element EgRep (Marin et al. 1993) was found to be highly expressed in the cyst wall and protoscoleces of *E. granulosus*, and similar lncRNAs were reported to be found among ESTs of *E. multilocularis* (Parkinson et al. 2012). By mapping ESTs of *E. multilocularis* and *E. granulosus* to their recently published genomic sequences, we have found that these lncRNAs are transcribed from many loci that have all of the characteristics of short nonautonomous retrotransposons (TRIMs), and which we have denominated *ta-TRIMs*. These characteristics include: 1) Two LTRs of approximately 198 bp, starting with 5′-TG-3′ and finishing with 5′-CA-3′ (i.e., with 5′-CA-3′ at both 3′-ends); 2) a PBS with nine bases of complementarity to the 3′-end of an *Echinococcus* ^Leu^tRNA, positioned at four bases from the end of the 5′-LTR; and 3) a PPT of 15 bases, located three bases upstream of the 3′-LTR (figs. 1 and 3). Between both LTRs, there are approximately 590 bp that lack any open reading frames longer than 120 codons, and show no similarity by BLASTX to any known proteins. This region may contain the packaging signal (PSI), a region of secondary structure which is not conserved at the level of primary sequence, but which is of importance for the packaging of retroviral RNAs with Gag proteins (Wicker et al. 2007). Flanking the LTRs, short (4–5 bp) target duplication sites can be found in many of the elements, as is characteristic of retrotransposons and retroviruses after integration. All of these characteristics strongly indicate that *ta-TRIM*s are nonautonomous retrotransposons that can be mobilized from an autonomous element *in trans*.

From full-length ESTs of both species, and from RNA-Seq data and 3′-RACE experiments in *E. multilocularis*, the transcription initiation site and the polyadenylation site could be clearly identified within the 5′-LTR and the 3′-LTR, respectively (fig. 1). Polyadenylation of different TRIM loci occurred within a 6-bp window in the 3′-LTR, upstream of which no canonical polyadenylation signal (AATAAA) could be found. Instead, there is an A/T rich region, containing in most cases an alternative polyadenylation signal previously proposed for *Echinococcus* spp., AATATA (Konrad et al. 2003; Koziol et al. 2009) located 12–15 bp upstream of the cleavage and polyadenylation site. Therefore, the LTRs could be unambiguously divided into U3, R, and U5 regions (fig. 1).

We searched for *ta-TRIM*s in the draft genomes of other taeniid cestodes (*T. solium*, *T. asiatica,* and *T. taeniaeformis*), as well as nontaeniid cestodes and other flatworms (*H. microstoma*, *M. corti*, *D. latum*, *S. mansoni,* and *Schm. mediterranea*). We could clearly find related *ta-TRIM* sequences in all *Taenia* spp., which were approximately 50% identical after alignment to the *Echinococcus ta-TRIM*s (supplementary data S1, Supplementary Material online). This comprised the conserved LTRs and also parts of the region between LTRs, including the crucial PBS and PPT. Furthermore, TSD sequences could usually be found flanking the LTRs of the full-length *ta-TRIM*s in these species (supplementary data S1–S4, Supplementary Material online). In contrast, no similar sequences could be detected in other flatworms, or in GenBank. Because the analyzed species of *Taenia* and *Echinococcus* cover all of the major lineages of taeniid cestodes (Knapp et al. 2011; Nakao et al. 2013), this indicates that *ta-TRIM*s originated at the base of the taeniid tree and are specific for this family.

We identified the *ta-TRIM* elements of *E. multilocularis* by BLASTN searches and by HMM and divided them into complete/near-complete elements (“full-length elements,” containing two recognizable LTRs, a PBS, and/or a PPT), or into partial elements. At least 142 full-length elements can be found in *E. multilocularis*, but many have substitutions in presumably important positions such as the 5′-CA-3′ motif at the LTRs, or within the PBS and PPT, resulting in only 19 perfect elements (i.e., elements that could be mobilized in principle as they have all the necessary *cis* sequences) (supplementary data S2, Supplementary Material online). In addition, a total of 1,939 loci in the genome show significant similarity by BLASTN and HMM searches to the LTR regions in the *E. multilocularis* genome (with hits located less than 1 kb apart counted as single hits). Pairwise divergence between full-length copies was between 4% and more than 60%, not taking into account linked copies (less than 30 kb distance) with less than 1% divergence. These numbers may change as newer versions of the *E. multilocularis* assembly are released, but are likely to be close to the real numbers given the high quality of the current version (Tsai et al. 2013). The *ta-TRIM*s of *E. multilocularis* were found dispersed in all chromosomes (supplementary data S2 and S5, Supplementary Material online), with no detectable compositional bias in the surrounding regions. No chromosomal regions showed an overrepresentation of *ta-TRIM*s except for a few examples of tandem repeats. Remarkably, these closely located *ta-TRIMs* have highly similar sequences (and cluster together in phylogenetic analyses), suggesting that these tandem elements were generated by unequal crossing over, and not because of a bias of the integration process (data not shown). Among the partial *ta-TRIM* sequences, many consisted of “solo-LTRs,” that is, isolated LTR elements. Solo-LTRs are a common derivative of LTR-retrotransposons, and are thought to be originated from unequal crossing-over between LTRs of a single element (Vitte and Panaud 2003). Consistent with this origin, many of the solo-LTRs are flanked by TSDs (see below).

Similar results were found in the *E. granulosus *and *T. solium* draft genomic assemblies (supplementary data S3 and S4, Supplementary Material online). In particular, although lacking TSDs, it seems that the EgRep element previously described for *E. granulosus* by Marin et al. (1993) consists of a solo-LTR from a *ta-TRIM* element, embedded within a larger, sparsely repeated sequence lacking any other distinctive elements (data not shown). In *E. granulosus*, there are at least 1,183 loci with similarity to *ta-TRIM* elements. In the case of *T. solium*, the total number of copies is difficult to assess given the fragmentary nature of the genomic assembly, but at least 827 loci show significant similarity to *ta-TRIMs*, including 24 full-length elements, of which 5 were perfect (using the same definitions as for *E. multilocularis*). Only a superficial analysis of the number of *ta-TRIM*s was performed for the unpublished provisional drafts of *T. asiatica* and *T. taeniaeformis*. Results in *T. asiatica* were similar to *T. solium*, and at least three perfect *ta-TRIM* elements could be found. In contrast, in *T. taeniaeformis*, although some almost complete *ta-TRIMs* could be found, they were divergent and had substitutions in key positions, suggesting that they are pseudoelements.

### Evolution and Retrotransposition Events of *ta-TRIMs*

**Fig. 2.—:**
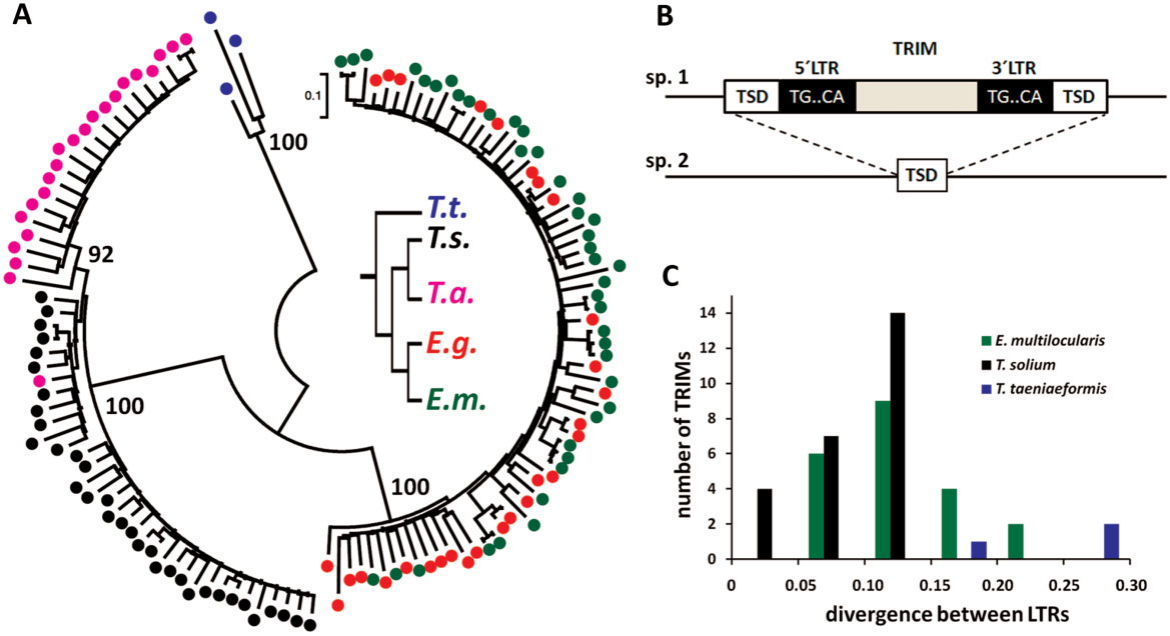
Evolution and insertions of *ta-TRIM*s. (*A*) Phylogenetic tree of *ta-TRIM*s from taeniid species, inferred by maximum-likelihood analysis (Kimura 2-parameter model with gamma distributed sites). Bootstrap values (1,000 replicates) are indicated next to selected nodes. The inset shows the tree of taeniid species (drawn from the data of Nakao et al. 2013), using the same color code as for the species of the *ta-TRIM* elements. *Eg*, *E. granulosus*; *Em*, *E. multilocularis*; *Ta*, *T. asiatica*; *Ts*, *T. solium*; *Tt, T. taeniaeformis.* (*B*) Diagram explaining the identification of insertion sites between closely related species (sp.1 and sp.2). (*C*) Histogram showing the divergence between 5′- and 3′-LTRs for *ta-TRIMs* of three taeniid species (see the text for details).

Strikingly, *ta-TRIM*s showed high similarity between *E. granulosus* and *E. multilocularis*, as well as between *T. solium* and *T. asiatica* (>90% for the most similar copies), but had much lower similarity when comparing other species pairs (approximately 50%). Phylogenetic analysis of *ta-TRIM*s from all the analyzed species shows that the elements of *Echinococcus* spp. form a well-supported clade, as do those of *T. asiatica* plus *T. solium*, and those of *T. taeniaeformis* (fig. 2*A*). Furthermore, most *T. asiatica* elements form a monophyletic clade. This topology suggests that massive independent expansions of *ta-TRIM* elements occurred in each of the main taeniid lineages, as well as in *T. asiatica*. Alternatively, the reciprocal monophyly between the elements of each species could be the consequence of extensive gene conversion between all of the copies of each genome. We searched for evidence of ongoing gene conversion between copies of *ta-TRIM*s in each species using the program GENECONV (Sawyer 1989, 1999). No indications were found in *E. multilocularis*, *E. granulosus*, *T. asiatica* or *T. taeniaeformis*, but in *T. solium* four examples were found that showed statistically significant evidence of gene conversion (global *P* values < 0.05). The low levels of gene conversion suggest that the first hypothesis is correct. However, we cannot discard the possibility that gene conversion was more extensive in the past, during the early expansion of *ta-TRIM*s.

**Fig. 3.—:**
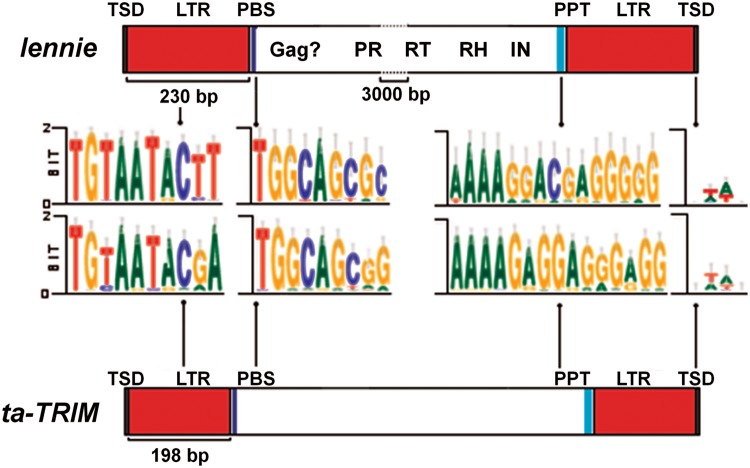
Comparison of *ta-TRIM*s and *lennie* elements. Diagrams to scale of idealized *ta-TRIM* and *lennie* elements from *E. multilocularis*, and direct comparison of sequence logos of selected features from both elements (from left to right, the 5′ of the LTR, the PBS, the PPT, and the TSD). Logos were created from sequence alignments of *ta-TRIM*s or *lennie* elements with WebLogo 3.4 (Crooks et al. 2004). In addition to PR, RT, RH, and IN domains, there is an upstream ORF in *lennie* that could code for Gag but does not show similarity to any sequences outside flatworms (data not shown).

We searched for evidence of the occurrence of *ta-TRIM* integration events after the splitting of *E. multilocularis* and *E. granulosus*, a relatively recent event that was estimated to have occurred between 2.5 and 9.2 Ma (Knapp et al. 2011). For this, we compared the flanking sequences of full-length elements from *E. multilocularis* (at least 2 kb upstream and downstream of each *ta-TRIM*) with the homologous regions of *E. granulosus* (fig. 2*B*). In three of seven comparisons, strong evidence of integration after the divergence of both species could be found, as the *ta-TRIM* is precisely lacking in the equivalent region in *E. granulosus*, and at that position a sequence can be found that is identical or has only one mismatch to the flanking TSD of the *E. multilocularis ta-TRIM* (fig. 2*B* and supplementary data S6, Supplementary Material online). With the same strategy, we searched for integration events after the splitting of *T. solium* and *T. asiatica*, and from five regions that could be compared between these assemblies, three examples showed strong evidence of integration after the splitting of these species (supplementary data S6, Supplementary Material online). Using less restrictive criteria (searching only 300 bp surrounding each *ta-TRIM* and disregarding whether the TSD is present or not) we could find 12 examples suggesting insertion of *ta-TRIM*s in *E. multilocularis* after the divergence of *E. granulosus*, and 2 analogous examples suggesting insertions that are specific for *E. granulosus*.

As a result of the particular mechanism of reverse transcription of LTR retrotransposons, both LTRs of each element are initially identical at the time of insertion (Telesnitsky and Goff 1997). Therefore, nucleotide identity between the 5′- and 3′-LTR of each *ta-TRIM* can be used to estimate the time of integration (Kijima and Innan 2010), if the rate of neutral nucleotide substitution is known (calibrated from the fossil record, or from biogeographic vicariance estimates). An important assumption for this method is that gene conversion must not occur between the LTRs (Kijima and Innan 2010). The size of the LTRs of *ta-TRIM*s is around or below the limit for efficient gene conversion in other systems (around 200 bp in mice, and approximately 300–500 bp in humans [Chen et al. 2007]). Furthermore, it has been shown in other models that gene conversion is very low when LTRs are very close (<4-kb distance; Kijima and Innan 2010). Therefore, this assumption seems to be reasonable in our case. Any gene conversion events between LTRs of different *ta-TRIM*s would be expected to increase their observed divergence values, increasing their estimated age of insertion. Thus, any estimates obtained would be a conservative maximum age.

We thus analyzed the divergence between 5′- and 3′-LTRs for full-length *ta-TRIM*s with intact LTRs, for one species of each main taeniid lineage (*E. multilocularis*, *T. solium,* and *T. taeniaeformis*; fig. 2*C*; supplementary data S7, Supplementary Material online). In *E. multilocularis*, divergence values between LTRs were between 6.1% and 22.9% (*n* = 21), whereas in *T. solium* they were between 2.2% and 14.9% (*n* = 25), and in *T. taeniaeformis* between 19% and 27% (*n* = 3). The distribution of the divergence values of LTRs was significantly different between all species pairs (Mann–Whitney *U*-test, Bonferroni correction, *P* < 0.02). Assuming equal substitution rates for all lineages, this implies that the waves of retrotransposition occurred at different time points for each lineage, and the relatively large divergence in *E. multilocularis* and *T. taeniaeformis* LTRs further suggests that in these species *ta-TRIM*s may be no longer active.

There is no fossil record for taeniids, but the neotropical sister species *E. vogeli* and *E. oligarthrus* were proposed to have split during the great American biotic interchange, 3 Ma (Knapp et al. 2011). Unfortunately, there is only very limited sequence information for these taxa. Given the low number of synonymous substitution sites available for analysis, and as we were interested in calibrating noncoding sequences (which can have different rates of neutral substitution to coding sequences; Subramanian and Kumar 2003; Hoffman and Birney 2007), we obtained instead an estimate for the divergence of neutral intronic sequences (Hoffman and Birney 2007) in two available genes. The estimated neutral substitution rate for introns in these species was 7.1 × 10^−^^9^ substitutions per site per year, which is well within the margin of rates described for neutral substitutions in other metazoans (Bowen and McDonald 2001; Gillooly et al. 2005; Hoffman and Birney 2007). By applying the substitution rate found for introns on the substitution values of LTRs (corrected using the K2P+G model), we estimated that the most recent *ta-TRIM* insertion occurred 0.84 × 10^6^, 4.76 × 10^6^, and 13.3 × 10^6^ years ago for *T. solium*, *E. multilocularis*, and *T. taeniaeformis*, respectively. Furthermore, by directly comparing the divergence values of LTRs with the divergence values found for introns between *E. multilocularis* and *T. solium* (30.3 ± 3.1%, *n* = 7 genes), it is apparent that most insertions must have occurred after the divergence of both lineages. In contrast, divergence in introns between *E. multilocularis* and *E. granulosus* (3.9 ± 0.76%, *n* = 8 genes) is of the same magnitude as between the most similar LTRs in *E. multilocularis*, suggesting that the last insertions in *E. multilocularis* occurred approximately at the same time as these species diverged.

In summary, we found evidence for retrotransposition of *ta-TRIM* elements after the divergence of the main lineages of Taeniidae, as well as after the splitting of *E. multilocularis* and *E. granulosus*, and after the splitting of *T. solium* and *T. asiatica*. These elements may still be active (or may have been active until very recently) in *T. solium*, whereas in *E. multilocularis* and *T. taeniaeformis*, it seems that they do not mobilize any longer.

### A Candidate Autonomous Element for the Mobilization of *ta-TRIMs in Trans*

So far, no specific retrotransposons have been proposed to be responsible for the mobilization of TRIMs in any species (Schulman 2012), probably because the similarity between them may be very limited. Initially, we unsuccessfully looked for candidate retrotransposons in *E. multilocularis* by searching for a short distance (<20 kb) between BLASTN searches for *ta-TRIM*s and TBLASTN searches for RTs (data not shown). As an alternative, we characterized some of the most complete LTR-retrotransposons in the *E. multilocularis* genome, and compared their sequence with the *E. multilocularis ta-TRIM*s. We found one family of LTR-retrotransposons (which we have dubbed *lennie*) that has characteristics suggesting that it may have fulfilled this role (fig. 3). In particular, the first eight bases of the LTR which are crucial for interaction with IN (Zhou et al. 2001) are identical between *ta-TRIM*s and *lennie*. Furthermore, as the cognate tRNAs are specifically packaged into VLPs during the retrotransposition cycle (Boeke and Stoye 1997; Mak and Kleiman 1997), any autonomous elements mobilizing *ta-TRIM*s should have the same PBS. *lennie* has a PBS of eight bases that is complementary to the same ^Leu^tRNA as *ta-TRIM*, and is positioned at the same distance from the 5′-LTR. Finally, the length of the TSD generated is characteristic for each IN group (Wu et al. 2005; Ballandras-Colas et al. 2013). Most TSDs of *ta-TRIM*s and solo-LTRs in *E. multilocularis* are four bases long (52/57 analyzed cases, with five cases that are five bases long), which is coincident with the length of the TSDs for *lennie* elements and their derived solo-LTRs (63/65 analyzed cases with TSDs of four bases, with two cases of five bases; the proportion of TSDs with four and five bases is not significantly different between *ta-TRIM*s and *lennies*, Fisher’s exact test *P* > 0.1). Although we could observe some expression of *lennie* elements from ESTs and RNA-Seq (data not shown), we were unable to detect intact copies of *lennie* in the *E. multilocularis* genome (all had at least one frameshift within *pol*). This suggests that although *lennie* elements are transcriptionally active, they are probably unable to complete the retrotransposition cycle. If *lennie* was the element mobilizing *ta-TRIMs in trans*, then the lack of intact *lennie* elements could explain the absence of recent *ta-TRIM* retrotransposition events in *E. multilocularis*.

Due to the draft status of the genome assemblies of *Taenia* spp., we were unable to determine whether intact *lennie* elements are present in these species. However, in all the analyzed *Taenia* spp., the TSDs of *ta-TRIM*s were five bases long (*T. solium*, 8/8 events; *T. taeniaeformis*, 2/2 events; the proportions of four-base-long and five-base-long TSDs are significantly different between *T. solium* and *E. multilocularis* by Fisher’s exact test, *P* < 1 × 10^−^^6^). This suggests that the element mobilizing the *ta-TRIMs* may have been different in *Taenia* spp.

### Massive Expression of *ta-TRIMs* and Generation of Novel Transcripts from Solo-LTRs

Originally, the transcripts of *ta-TRIM*s were noticed in *E. granulosus* due to their massive expression (approximately 10% of all ESTs in oligo-capped libraries; Parkinson et al. 2012). Here, we analyzed a small collection of full length ESTs from *E. multilocularis* metacestodes, and found that also in this species, *ta-TRIMs* are very highly expressed (1.8% showed significant [<e-5] similarity to *ta-TRIM*s by BLASTN, *n* = 4,195 ESTs). In *T. solium*, a similarly high proportion of ESTs in GenBank showed similarity to *ta-TRIM*s, (1.4%, *n* = 74,730 ESTs).

**Fig. 4.—:**
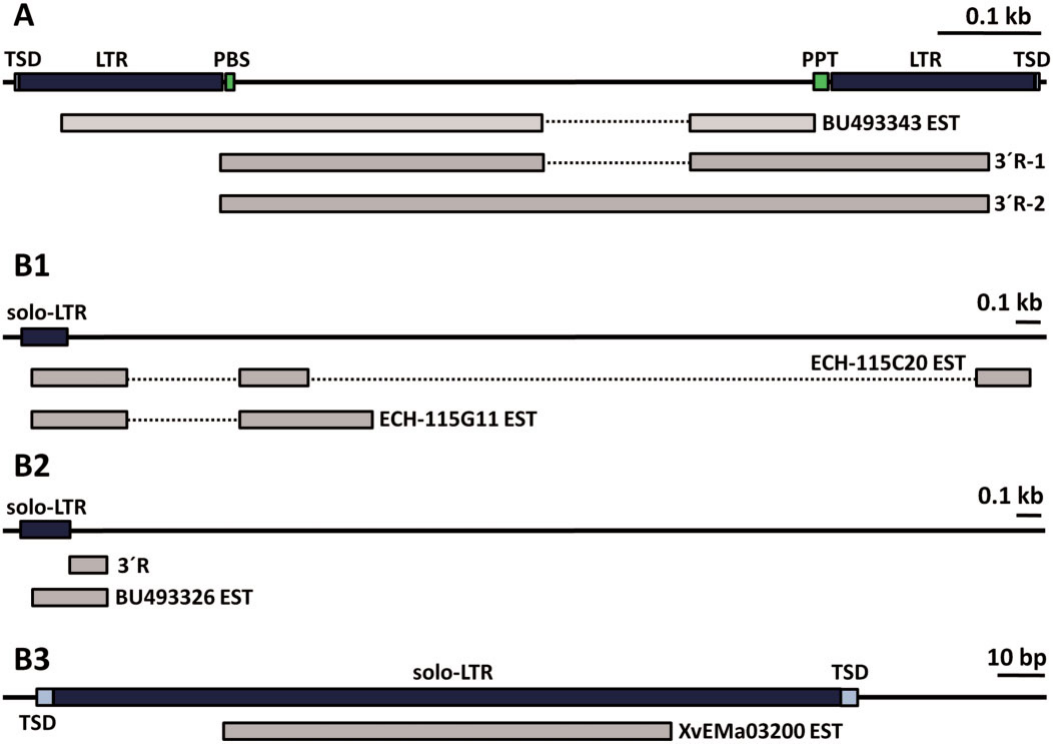
Transcription of *ta-TRIM* and derived elements in *E. multilocularis*. Continuous lines indicate the genomic loci, and EST and 3′-RACE (3′ R) data are shown below, with mapped regions drawn as gray rectangles, and intervening introns as dotted lines connecting them. (*A*) Example of a full length *ta-TRIM*. (*B*1–*B*3) Examples of solo-LTRs initiating the transcription of diverse noncoding RNAs (see the main text for details).

**Fig. 5.—:**
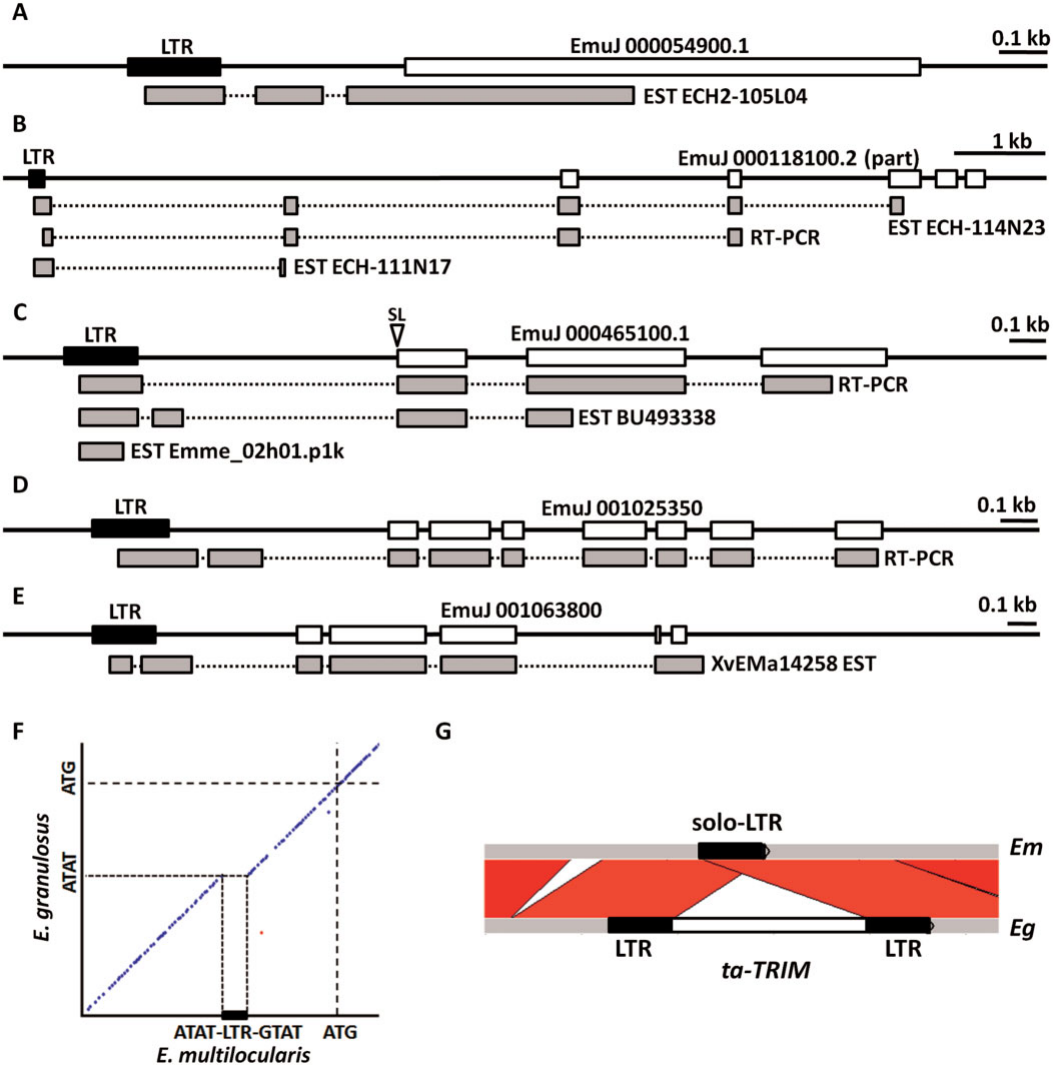
Transcriptional fusions of solo-LTRs and downstream coding genes. Drawings are as in figure 4. “RT-PCR” indicates sequenced RT-PCR products obtained in this work. The white arrowhead (SL) in panel (*C*) indicates the position of the *trans*-splicing acceptor site in the ortholog of *E. granulosus*. Not all alternative splicing isoforms that were found are shown. (*A*) Locus 2. (*B*) Locus 8. (*C*) Locus 21. (*D*) Locus 60. (*E*) Locus 64. (Loci numbers are as found in supplementary data S8, Supplementary Material online). (*F*) Dot plot (identity within 10-bp windows is shown as dots) of the 2-kb region upstream of the start codon (ATG) of EmuJ_000465100 (Locus 21: horizontal axis) and the ortholog region of *E. granulosus*. The solo LTR (rectangle) is precisely lacking in *E. granulosus*, and a similar sequence (5′-ATAT-3′) is seen at this position as in the TSDs of the solo-LTR of *E. multilocularis*. (*G*) Comparison of the upstream region of locus 64 in *E. multilocularis* (Em), where a solo-LTR is found, with the ortholog region in *E. granulosus *(Eg), containing a full length *ta-TRIM*. Diagonal bars indicate BLASTN hits. Drawing generated with WebACT (Abbott et al. 2005).

By using diverse EST libraries of *E. multilocularis* (see Materials and Methods) we searched for *ta-TRIM*-like transcripts by BLASTN and mapped them to the genome of *E. multilocularis* under stringent requirements (single mapping position in the genome with greater than 99% identity to the EST). In this way, we identified 73 different loci with similarity to *ta-TRIM*s and with strong evidence of transcriptional activity, which we then manually curated. For 63 loci, transcription was apparently originated from within an LTR (figs. 4 and 5; supplementary data S8, Supplementary Material online), strongly indicating that it was generated from an LTR promoter and not by read-through from upstream genes.

In total, 25 loci corresponded to full-length *ta-TRIM*s, which in most cases showed several deletions and substitutions of presumably important sites, but which nonetheless had two LTRs (fig. 4*A*). Alternative *cis*-splicing events were sometimes detected within the *ta-TRIM* for these loci, and were confirmed for one locus by 3′-RACE (fig. 4*A*). No transcriptional read-through was detected downstream of the U5 region of the 3′-LTR for full-length *ta-TRIM*s. The other 38 loci corresponded to transcription initiation from solo-LTRs, and these showed two main distinct behaviors. In some cases, solo-LTRs generated internal transcripts, from the U3/R limit to the R/U5 limit, indicating that in the absence of *ta-TRIM* sequences downstream to the LTR, short “abortive” transcripts are generated (fig. 4*B*, example 3). These are reminiscent of short transcripts originating from mutant retroviruses in which polyadenylation is no longer repressed in the 5′-LTR (Schrom et al. 2013). For 16 loci, only this kind of transcripts was found. Other solo-LTRs (22 loci) showed transcriptional read-through into the downstream neighboring regions. In most cases this resulted in the generation of lncRNAs, which were in many cases *cis*-spliced (fig. 4*B*, panels 1 and 2). In five loci, the solo-LTR promoter initiated transcripts that were fused to a downstream coding gene, generating an alternative isoform under the control of the solo-LTR (fig. 5). The predicted amino acid sequences of the downstream genes were always conserved (at least among all cestodes analyzed) strongly indicating that they correspond to protein-coding genes.

We further confirmed by RT-PCR these transcriptional fusions for three of four investigated loci (the fourth transcriptional fusion may also occur, but the RT-PCR unspecifically amplified a transcript from a different *ta-TRIM* locus). Control RT-PCRs in which the forward primer was located ≤200 bp upstream of the solo-LTRs, as well as in the U3 region within the LTR, gave no amplification, providing strong confirmatory evidence that the transcript is initiated within the solo-LTR (data not shown). For most loci, analysis of RNA-Seq data suggested that the new LTR promoter is a minor alternative one, but in the case of locus 8 (shown in fig. 5*B*) it seems to be the main or only promoter (data not shown).

Very interestingly, three of the loci producing fused transcripts between solo-LTRs and coding genes were found to differ between *E. multilocularis* and *E. granulosus*. In one case, a full-length *ta-TRIM* element was found in the corresponding *E. granulosus* region, which presumably would not generate fusion transcripts (locus 64, fig. 5*G*). No *ta-TRIM* or solo-LTR was found for the other two cases in *E. granulosus* (loci 8 and 21), and because the loci are collinear except at the precise position of the solo-LTR, this suggests that a *ta-TRIM* was inserted and reduced into a solo-LTR after the divergence of both species (fig. 5*F* and data not shown). The *E. granulosus* ortholog of locus 21 is a *trans*-spliced gene (evidence from two independent ESTs, BI244081.2 and CV681147.1, containing the *E. granulosus* spliced leader [Brehm et al. 2000] at the 5′-end). In *E. multilocularis* the LTR-initiated transcript is spliced *in cis* to an exactly corresponding splice acceptor site (fig. 5*C*), suggesting that it competes for this site with the spliced-leader.

We also analyzed ESTs from *E. granulosus*, which are however more difficult to map precisely given the draft status of the genomic assembly. Among these ESTs we could also find one example of a transcriptional fusion between an LTR and a downstream gene (supplementary data S9, Supplementary Material online).

In summary, some *ta-TRIM*s and derived solo-LTRs are massively transcribed in *E. granulosus*, *E. multilocularis,* and *T. solium*. In *E. multilocularis*, we provide strong evidence for the generation of novel lncRNA and alternative isoforms for coding genes from dispersed solo-LTRs. Some of these *ta-TRIM* elements are absent in the corresponding regions in *E. granulosus*, suggesting that these differences were generated during or after their speciation.

### RNA-Seq Analysis of *E. multilocularis* Indicates that Only a Fraction of the *ta-TRIMs* Is Active throughout the Life Cycle

We analyzed previously published high-throughput RNA-Seq data for *E. multilocularis* larval and adult stages (Tsai et al. 2013), in order to determine how many of the *ta-TRIM* elements are active, and how expression of *ta-TRIM*s changes through the life cycle. These data sets consist in single RNA-Seq experiments of pregravid and gravid adults, metacestode vesicles (without protoscoleces), dormant protoscoleces and activated protoscoleces (activated by pepsin and low pH treatment), and primary cell cultures that are undergoing metacestode regeneration (as a proxy for the early stages of metacestode development [Olson et al. 2012]). As a reference, an overview of the life cycle of *E. multilocularis* is shown in supplementary data S10, Supplementary Material online.

**Fig. 6.—:**
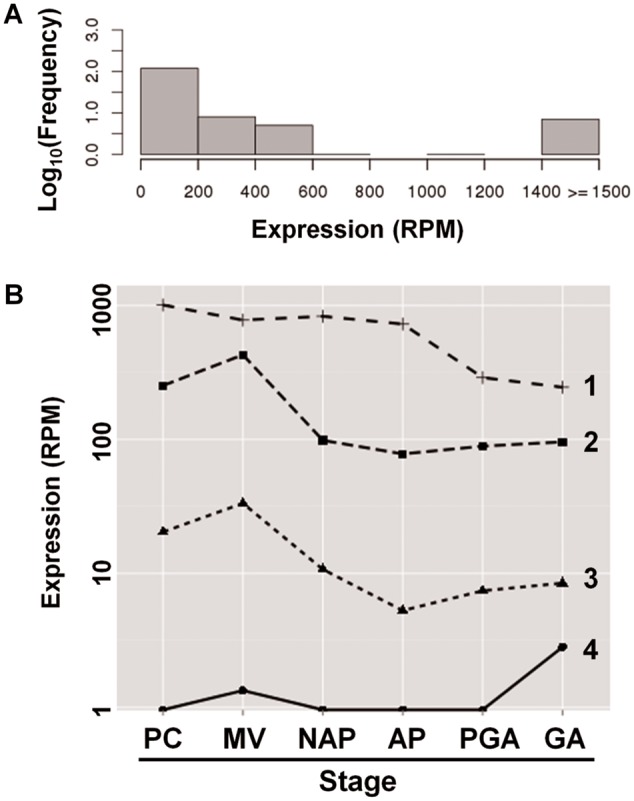
RNA-Seq analysis of *ta-TRIMS* in *E. multilocularis*. (*A*) Histogram showing the distribution of expression levels of individual full-length *ta-TRIM*s (average of reads per data set, normalized per 10^6^ uniquely mapping reads [RPM]). (*B*) Expression of representative individual full-length *ta-TRIM* elements (in Log scale) across data sets (PC, primary cells; MV, metacestode vesicles; NAP, nonactivated protoscoleces; AP, activated protoscoleces; PGA, pregravid adults; GA, gravid adults). For the selection of the representative elements, all *ta-TRIM*s were divided into four bins according to their RPM values (1–4), and the *ta-TRIM* with the median RPM value for each bin was selected and graphed.

Using highly conservative settings (see Materials and Methods), we determined the expression levels for each *ta-TRIM* copy. Remarkably, *ta-TRIM*s are very unequally expressed: A few appear transcriptionally active, whereas the majority is not transcribed (fig. 6*A* and supplementary data S11–S13, Supplementary Material online). Indeed, 11% of all *ta-TRIM*s generate over 90% of uniquely mapping reads. The different *ta-TRIM* copies seem to be either always expressed or always silent across life stages (i.e., there is a high level of correlation between the expression levels of individual *ta-TRIM* copies across the different libraries), suggesting that silencing of *ta-TRIM* elements can be stably inherited (fig. 6*B* and supplementary data S11–S13, Supplementary Material online). This is further supported by the fact that these data sets were obtained from different *E. multilocularis* isolates, suggesting that the expression levels of individual *ta-TRIM* copies are stably maintained in different lineages. These transcriptionally active *ta-TRIM*s are approximately evenly distributed across the genome (supplementary data S11, Supplementary Material online). There is a highly significant correlation of the expression levels of individual *ta-TRIM*s as determined from EST and from RNA-Seq data (Pearson correlation test, *P* < 0.00001), confirming that the variation observed among *ta-TRIM*s is not an artifact of the mapping algorithm or the selected parameters.

### Expression of *ta-TRIMs in E. multilocularis* Germinative Cells

**Fig. 7.—:**
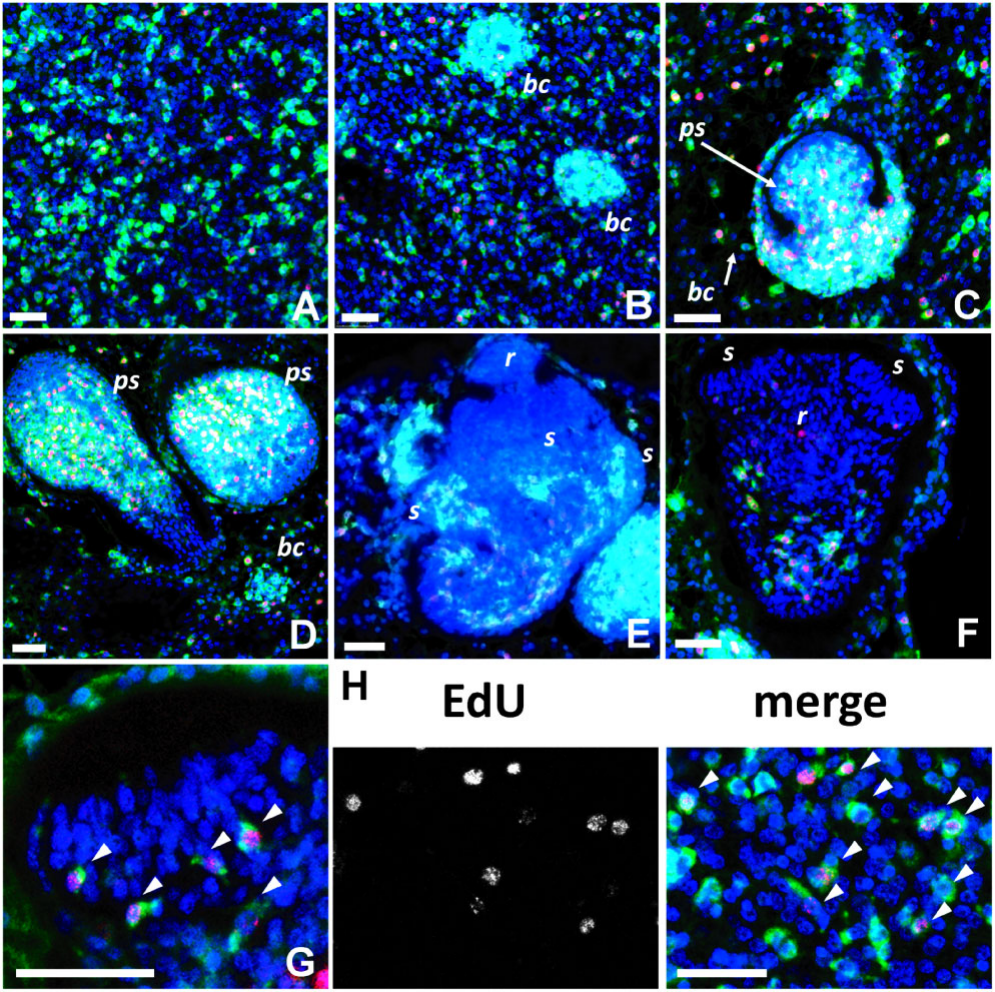
WMISH analysis of *ta-TRIM* expression during *E. multilocularis* larval development. In all panels, the *ta-TRIM* WMISH signal is shown in green, DAPI (all nuclei) in blue, and EdU detection in red (EdU was incorporated during a 5 h, 50 μM pulse, in vitro). Staging follows the system of Leducq and Gabrion (1992). (*A*) Germinal layer. (*B*) Early formation of brood capsule buds (bc) from the germinal layer. (*C*) Early formation of the protoscolex (ps; stage 1). (*D*) Early formation of the protoscolex (ps; stage 2). (*E*) Intermediate protoscolex development (stages 3 and 4). *r*, rostellum; *s*, sucker primordia. (*F*) Late protoscolex development (already invaginating, stage 6). *r*, rostellum (red signal in rostellum comes from auto-fluorescence of the hooks); *s*, suckers. (*G*) Detail of the sucker of the protoscolex shown in (*F*). Arrowheads point at EdU^+^ *ta-TRIM *^+ ^cells at the base of the developing suckers. (*H*) Detail of the germinal layer. Arrowheads point at EdU^+^ *ta-TRIM*^+ ^cells. Bars, 25 μm.

Finally, we performed WMISH with a *ta-TRIM* probe to analyze at the cellular level the expression of *ta-TRIM*s during the development of the metacestode larva of *E. multilocularis*. Within the metacestode cysts, the cells are organized as a thin layer (the germinal layer), which has dispersed germinative cells (stem cells). These germinative cells are the only proliferating cell type in the larva, whereas all differentiated cells are postmitotic (Koziol et al. 2014). The *ta-TRIM* probe showed a strong and specific signal in dispersed cells with the characteristic morphology of the germinative cells (small sized, pear-shaped to fusiform cells with a high nucleo-cytoplasmic ratio, and one or more large nucleoli [Reuter and Kreshchenko 2004; Koziol et al. 2014]) (fig. 7*A* and *H*). Furthermore, they were in average 23% of all cells in the germinal layer (*n* = 2 independent WMISH experiments with two different isolates, 1,130 total cells counted), which corresponds well to the proportion of germinative cells as estimated by morphology (21–25% of all cells; Koziol et al. 2014). Finally, the *ta-TRIM* signal is observed in 94.3% of all cells undergoing S-phase, as determined by their incorporation of the thymidine analog 5-ethynyl-2′-deoxyuridine (EdU) (*n* = 3 independent WMISH experiments with three different isolates, 427 total cells counted). All these results strongly indicate that *ta-TRIM*s are specifically expressed in the germinative cells, and only very low or null levels can be seen in other cells. Furthermore, *ta-TRIM*s appear to be expressed in almost all of the proliferating germinative cells, given the high colocalization of *ta-TRIM*s with EdU incorporation.

Throughout the development of protoscoleces from the germinal layer, the *ta-TRIM* + cells show the stereotypical distribution of the germinative cells (Koziol et al. 2014) and continue to incorporate EdU (fig. 7*B*–*G*). They massively accumulate during the early budding process that leads to the formation of brood capsules (thickenings of the germinal layer that invaginate into the cyst, where protoscolex development proceeds [Koziol et al. 2013]; fig. 7*B*), as well as during early and intermediate protoscolex formation, except at the apical most region where no proliferation is observed (fig. 7*C* and *D*). At the last stages of development, expression is restricted to ever decreasing cell numbers in the posterior body, whereas in the scolex (the head of the protoscolex with the attachment organs) expression is only observed in the proliferative region at the base of the suckers (Galindo et al. 2003; Koziol et al. 2014) (fig. 7*E*–*G*). In summary, during the development of the metacestode larva, the *ta-TRIM* WMISH signal is strongly and specifically observed in the germinative cells.

## Discussion

### Convergent Reductive Evolution of LTR-Retrotransposons into TRIMs

In this work, we describe a new family of TRIM elements that is found exclusively in taeniid cestodes, and has expanded during the divergence of this family. So far, TRIM-like elements have been described in plants (Witte et al. 2001; Kalendar et al. 2008), fungi (Dos Santos et al. 2012), and in two distant metazoan groups: Ants (Zhou and Cahan 2012) and Taeniid cestodes (this work). The TRIM elements of each of these taxonomic groups show no similarity to each other, and are therefore a likely case of convergent reductive evolution, in which different retrotransposons are stripped of all nonessential sequences as they parasitize autonomous elements for retrotransposition (Schulman 2012). As such, TRIM-like elements are probably widely present in the genomes of other groups, but their discovery is difficult as they possess no similarity to other TRIM-like elements, have only short LTRs, and lack coding sequences. In our case, the discovery was prompted by the high expression of these elements in cestodes, but silent or lowly expressed TRIMs could easily be overlooked in typical searches for LTR elements.

The distribution of individual TRIM families in animals seems to be more restricted than in plants, as similar TRIM elements are found throughout mono and dicotyledons (Witte et al. 2001; Kalendar et al. 2008). Interestingly, very similar TRIMs have been described in distant ant species and the tree of TRIM elements in ants shows no correspondence with the species tree, suggesting horizontal transfer events between species (Zhou and Cahan 2012). In sharp contrast, we find that phylogenetic clades of *ta-TRIM*s are specific to individual taeniid lineages (fig. 2*A*). This is strong evidence against horizontal transference of these elements, and suggests that independent expansions of *ta-TRIM*s occurred in each lineage. Further support for this comes from the fact that the estimated insertion dates are significantly different for *ta-TRIM*s of each species investigated (fig. 2*C*), and that specific insertions occurred after the divergence of closely related species (fig. 2*B*). However, we cannot rule out the possibility that the phylogenetic pattern is caused by extensive gene conversion between *ta-TRIM* elements in each genome. In general, ectopic gene conversion would not be expected to maintain sequence similarity for such a large number of elements widespread throughout the chromosomes (Chen et al. 2007). However, it has been shown that LTR-retrotransposons in *Saccharomyces cerevisiae* can undergo ectopic gene conversion between the genomic DNA copies and the cDNA intermediates (Melamed et al. 1992), and similar mechanisms have been proposed to mediate gene conversion between nonlinked loci in other organisms (Kass et al. 1995; Benovoy and Drouin 2009). Therefore, high levels of retrotransposon cDNA could result in genome-wide gene conversion events. Furthermore, we observed evidence for ongoing gene conversion in *T. solium*. At this point, it is not possible to decide between both explanations for the observed phylogenetic pattern, but large scale studies of synteny of *ta-TRIM*s in all the species may be able to distinguish between them, once less fragmentary genomic assemblies are available.

Except for a few cases in which the retrotransposon and the nonautonomous element showed extensive sequence similarity (e.g., the LARD *Dasheng* and the retrotransposon *RIRE-2* in rice) (Jiang et al. 2002), few pairs of nonautonomous-elements and their possible mobilizing retrotransposons *in trans *have been proposed. In the case of TRIMs, no candidate has been identified so far. It is possible that in TRIMs, extensive sequence reduction has limited similarity to a few key positions for interaction with RT and the primer tRNA (the PPT and PBS) and with INT (the ends of the LTRs), and that most of the specificity is achieved by the specific packaging of the TRIM RNA into the VLP particle (Friedl et al. 2010). Packaging is dependent on the PSI element, which is only conserved at the secondary structure level (Wicker et al. 2007) and therefore difficult to identify. Based on this, we propose that the element *lennie* may have been the factor mobilizing *ta-TRIM*s in *E. multilocularis* (fig. 3). The absence of identifiable intact *lennie* elements in the genome could explain the lack of recent retrotransposition events in *E. multilocularis*, as well as the absence of gene conversion events (as no cDNA copies would be produced). Furthermore, it would be expected that competition with highly expressed *ta-TRIM* elements could drive the extinction of an autonomous retrotransposon group such as *lennie*, as the rate of their own retrotransposition decreases and the existing copies accumulate deleterious substitutions (Schulman 2012). In *Taenia* spp., the different size of TSDs as compared with *E. multilocularis* gives evidence that a different element may be the mobilizing factor *in trans*, as TSD size is specific for each IN group (Zhou et al. 2001; Ballandras-Colas et al. 2013). This implicates a unique shift of “host” (mobilizing factor) during the evolution and divergence of *ta-TRIM*s in taeniids.

### Expression of *ta-TRIM* Elements and the Generation of New Transcripts

The EST evidence suggests that in all the analyzed taeniid species, *ta-TRIM*s are transcribed at very high levels (>1% of all polyadenylated RNAs as determined from EST data). Furthermore, the presence of solo-LTRs has a clear effect in the expression of downstream sequences in *E. multilocularis*, resulting in many new lncRNA, and in alternative promoters for coding genes (figs. 4 and 5). Therefore, although the elements may no longer be active for retrotransposition in *E. multilocularis*, they have extensively modified its transcriptome. The evidence we show here is very conservative: more examples of lncRNA originating from solo-LTRs can be found when using slightly lower stringency values for the mapping of ESTs (data not shown), and it is likely that many more examples will be found with larger data sets, given the number of positions (>1,000) with similarity to *ta-TRIM*s in the genome. Usage of retrotransposons as alternative promoters and as a source of new lncRNA has been described for many individual examples, and also at a large global scale for model organisms such as humans, mice, and *Drosophila* (Peaston et al. 2004; Cohen et al. 2009; Faulkner et al. 2009; Kelley and Rinn 2012; Lenhard et al. 2012; Batut et al. 2013). It has been proposed that this can result in evolutionary innovation in the expression patterns of the involved genes, and may lead to the coordinately regulated expression of various genes, as they acquire the expression pattern of the invading retrotransposons (Peaston et al. 2004; Batut et al. 2013). The finding of splicing between a solo-LTR and an ancestral *trans*-splicing acceptor site (fig. 5*C*) provides a novel and simple evolutionary mechanism by which LTR retrotransposons can be “exapted” as alternative promoters. In this model, the pre-existing *trans*-splicing acceptor site (as found in *E. granulosus*) is spliced to an appropriate splice-donor site originating from the new LTR. This donor site may be there by mere chance, as it does not need to be a particularly strong one (e.g., a simple 5′-GT-3′ motif). This is because splicing has a “donor-first” syntax, in which a *cis*-splicing site efficiently out-competes the spliced-leader for a downstream splicing acceptor site (Hastings 2005).

The generation of lncRNAs from solo-LTRs seems to be a simple consequence of downstream transcriptional read-through. However, lncRNAs have been recently shown to have many functions for the regulation of gene expression *in trans*, and the transcription of lncRNA itself may also alter gene expression *in cis* (Mercer et al. 2009; Kornienko et al. 2013). Therefore, some of these new lncRNAs may have been exapted for new functions in *E*. *multilocularis*.

The differences in *ta-TRIM* transcriptional fusions between *E. multilocularis* and *E. granulosus* suggest that they could lead to differences in gene regulation between both species. Furthermore, because of the stem cell-specific expression of *ta-TRIM*s (fig. 7), this could lead to novel stem-cell specific transcripts. Future comparative analyses of the *ta-TRIM*-derived transcriptome of both species may identify many more examples. It is possible that the differences in *ta-TRIM*-derived transcripts between *Echinococcus* spp. have contributed to some of the important differences in larval morphology and development found between these closely related species (Thompson 1986). These differences have long been considered puzzling, particularly as the gene complement of both species is almost identical and no notable differences in genome organization had been identified between them so far (Olson et al. 2012; Tsai et al. 2013). The genes showing transcriptional fusions to upstream solo-LTRs have a variety of roles in important biological pathways. These include the exosome subunit RRP43, which binds and selects specific mRNAs containing AU-rich elements for degradation by the exosome (Anderson et al. 2006); CHMP5, a component of the ESCRT-III complex involved in multivesicular bodies formation (Shim et al. 2006); as well as conserved hypothetical proteins with no predicted molecular function.

### *ta-TRIMs *as a Germinative Cell Marker in *E. multilocularis*

The WMISH experiments (fig. 7) provide very strong evidence of a germinative cell-specific expression of *ta-TRIM*s throughout the development of the metacestode larva from the cyst wall to the mature protoscolex. The germinative cells of *E. multilocularis* larvae are a morphologically homogeneous population of undifferentiated cells, which however show heterogeneity at the molecular level in the expression of conserved stem cell regulators such as *nanos* and *argonaute* genes (Koziol et al. 2014). This suggests that there may be in reality several subpopulations with different proliferation and/or self-renewal potencies. In contrast, it seems that *ta-TRIM*s are expressed in almost all of the morphologically defined germinative cells, and are therefore the best molecular marker so far for the total germinative cell population. The small number of EdU^+^ *ta-TRIM^−^* cells (approximately 5% of all EdU^+^ cells) could indicate the existence of a different small subpopulation of germinative cells, or could be the result of fluctuating silencing of these elements in the germinative cells. At this point, nothing is known about the mechanism by which cestodes silence MGEs in the absence of a canonical PIWI pathway (Skinner et al. 2014), but a comparison of the chromatin structure and histone modifications between expressed and silent *ta-TRIM* copies may provide a first experimental strategy toward its elucidation, particularly as the silencing of specific copies seems to be stable across different isolates and life stages. It is possible that taeniid cestodes further control the expansion of *ta-TRIM*s by simply repressing the autonomous element mobilizing *ta-TRIMs in trans*. This would explain the absence of strong deleterious effects in the face of strong *ta-TRIM* expression.

From an evolutionary point of view, expression of *ta-TRIM*s in somatic stem cells which will eventually form all of the tissues of the next life stage (including the germ line) would allow the expansion and transmission of *ta-TRIM*s in the genome of the next generation. At this point, we do not know whether *ta-TRIM*s are also expressed in the germ line in the gonads of the adult stage. However, the analysis of published RNA-Seq data (Tsai et al. 2013) shows similar expression levels of *ta-TRIM*s in the adult stage, suggesting that this is possible. It would be interesting to determine whether in planarians, which can also generate the germ line from somatic stem cells after embryonic development, specific retrotransposon families also show stem-cell specific expression.

The expression of retrotransposons in somatic stem cells of *E. multilocularis* is analogous to the highly specific expression of several endogenous retrovirus families during early mammalian development, before the specification of the germ line (Brulet et al. 1985; Evsikov et al. 2004; Peaston et al. 2004), as well as in the germ line itself (Dupressoir and Heidmann 1996): Only expression at these stages may result in the expansion of endogenous retroviruses in the genome of the following generation. Indeed, expressed endogenous retroviruses have been shown to be excellent markers for totipotency or pluripotency in the early mammalian embryo and in embryonic stem cells (Santoni et al. 2011; Macfarlan et al. 2012; Wang et al. 2014). It has been shown that many genes specifically expressed in pluripotent embryonic cells are transcribed from similar upstream LTR promoters (Macfarlan et al. 2012; Fort et al. 2014), and this has been proposed to result in the concerted expression of genes important for pluripotency. The widespread presence in *E. multilocularis* of transcription from solo-LTRs and their presumably stem-cell specific expression suggest that similar mechanisms may be at play in the stem cells of taeniid cestodes.

## Supplementary Material

Supplementary data S1–S14 are available at *Genome Biology and Evolution* online (http://www.gbe.oxfordjournals.org/).

Supplementary Data
