# Supplementary material for: A Novel Terminal-Repeat Retrotransposon in Miniature (TRIM) Is Massively Expressed in Echinococcus multilocularis Stem Cells
Source: Genome Biol Evol. 2015 Jul 1;7(8):2136–53. doi: 10.1093/gbe/evv126 (PMC4558846; doi:10.1093/gbe/evv126)
Supplement: Supplementary Data [file supp_7_8_2136__index.html]

A Novel Terminal-Repeat Retrotransposon in Miniature (TRIM) Is Massively Expressed in Echinococcus multilocularis Stem Cells — Supplementary Data 

# A Novel Terminal-Repeat Retrotransposon in Miniature (TRIM) Is Massively Expressed in *Echinococcus multilocularis* Stem Cells

## Supplementary Data

files

- Supplementary Data - zip file
