# Supplementary material for: A Novel Terminal-Repeat Retrotransposon in Miniature (TRIM) Is Massively Expressed in Echinococcus multilocularis Stem Cells
Source: Genome Biol Evol. 2015 Jul 1;7(8):2136–53. doi: 10.1093/gbe/evv126 (PMC4558846; doi:10.1093/gbe/evv126)

Supplementary Data 1. Alignment of examples of *ta-TRIM* elements from taeniids.

```
                    10        20        30        40        50
            ....|....|....|....|....|....|....|....|....|....|
E multi     TGTAATACGATAGTGGTTT--ATCCCTCCTCATTGGTGAGGCGCCACTTT 48
T solium    TGTAATAC-----------------TTTGTTTGTTCGAACTACCGGTTTC 33
T asiatica  TGTAATACT----------------TT-GTTTGTTTGAACTGCCGGTTTT 33
T taeniaef  TGTAATGCTTTGTTTACCTGAATACCTGACTTTTAGCGA----TCACTTT 46

                    60        70        80        90       100
            ....|....|....|....|....|....|....|....|....|....|
E multi     CGTACTTTGAATTAGTCCCCTCGTGCCTCGAGGAGTTGGCCGGCCATTCG 98
T solium    GGTGACTTGGGT-GGCCATCTTGTACG--AGCCGGTTGGCCGGCC-TTTG 79
T asiatica  GGTCACTTGGGT-GGCCATATTGTACTG-AGCCGGTTGATGGCCC-TTTG 80
T taeniaef  AG-GCTTTGTCTGGA---CTTTGTACTC------GTGGGCTGGCC-TTCA 85

                   110       120       130       140       150
            ....|....|....|....|....|....|....|....|....|....|
E multi     TGGCATGCCAATAGCCCTGACACCCT-CTCTGTGCTGAACATGTACTCTA 147
T solium    GGCCGTGCCAACAGCCCCCAAGCCCT-CTGTTTCCAGAATATATAT--TC 126
T asiatica  GGCCGTGCCAACAGCCCCCAAGCCCT-CTGTTTCCAGAATACATGCGTTC 129
T taeniaef  GATCATGCTCA-GGCTCAGTTACTTTGCCCTGTTTAGAATACAGATATT- 133

                   160       170       180       190       200
            ....|....|....|....|....|....|....|....|....|....|
E multi     CCCGA---GTATTGTGTCTTCTTTCATCGTCTCTTGGGGGTCTCGGAACG 194
T solium    TGCCCC-AATACTATGCCTTCTTTTACC--TCAAAGGGAGTCCCGGGACG 173
T asiatica  TACCCCCAGTGCTATGCCTTCTTTTACC--TTAAAGGGAGTCTCGGGACG 177
T taeniaef  --------ATCCACTGCCTTCTTCCACC--CTATGAAAAGTCGCAGGACG 173

                   210 PBS   220       230       240       250
            ....|....|....|....|....|....|....|....|....|....|
E multi     CTACATTGCTGGCAGCGGAAAGCAAAACCGA-----TCTTAAAGGACATG 239
T solium    CTACACTGCTGGCAGCGGAAAGCGGACCCGA-----CATTGATGG-ATTT 217
T asiatica  CTACATTGCTGGCAGCGGAAAGCAGACCCGA-----CTTC-ATGG-ATTT 220
T taeniaef  CTACACTGGTGGCAGCAGAACGCGGGCCGGACATTTTTTTAACAA-CCTC 222

                   260       270       280       290       300
            ....|....|....|....|....|....|....|....|....|....|
E multi     TATACTG--ATTTTCCGCTTC-TGCGTAATAAAAGTTA-GTGGAGAAGAA 285
T solium    TATCCTGCTCCATGCCGCTTC-TGGCAAACTTTGGCGGTGTGAAAGAAGT 266
T asiatica  TATCCTGCTCCATGTCGCTTC-TGGCAAATTTTGGCGGTGTGAAAGAAGT 269
T taeniaef  TATCCTATCAAGTACCGTGTG-TTGCTTGTACCAATGGTGTGCAGAGGGG 271

                   310       320       330       340       350
            ....|....|....|....|....|....|....|....|....|....|
E multi     GGAGTACATATGCTGCGGATGTGAGAATGGT--GATGATCCCTAATAGAA 333
T solium    GCATGTGCTATGG-----ATGAGAGAACCTCA-GGTATCTCGTAATAGAA 310
T asiatica  GCATGTGCTATGG-----ATGGGAGAACCTCT-GGTATCTCGTAACAGAA 313
T taeniaef  GAATTAGCAATGGTG---ACAGGAGAATCCCTGAATCTCTCACAATGAAA 318
```

```
             360       370       380       390       400
        ....|....|....|....|....|....|....|....|....|....|
E multi     GGGTTGTTGTTGACGTTCACCTTCGGCATCCCTCCCAGTCCCGTGTTCTG 383
T solium    GGGCTGAGATCGACGTCCAGCTTCGGCATCTCCCTCTTTCCCGTGTTCTG 360
T asiatica  GGACCAAGATCGACGTCCAGCTTCGGCACCTCCCTCGCCCTCGTGTTCGA 363
T taeniaef  GGGTCGAGACTGGCGTCTGCACTTGGC-TTCACCTTATTCCCGAGTTCTA 367

             410       420       430       440       450
        ....|....|....|....|....|....|....|....|....|....|
E multi     CAGAAAAGCTGGTAACAACTGAAGCGACACCAGCCGTGAAC--------- 424
T solium    CAAGGAAGCTATTAACAACTGAAGCGACACTAGCCGTGAATTCCAAGCCC 410
T asiatica  CAGGGAAGCTATTAACAACTGAGGCGACACTAGCCGTGAACTCCAATTCC 413
T taeniaef  CAGACAAGCTATTAACAACTGAAGCGACACCA-CTGTGGAC--------- 407

             460       470       480       490       500
        ....|....|....|....|....|....|....|....|....|....|
E multi     ----CATCTG-------------GAAGT--TTCG----------TTACGG 445
T solium    AGTGCAACTGCCGAGAAGGATCAGTGACGTTTTGGTCAAGCATCCTCTGT 460
T asiatica  AGTTGAACTGTCGAGAGAGACCTGCGAAGTTGTGGTCAAGCATCCTCCGT 463
T taeniaef  ----CATATG-------------GAAGAGATTCG----------TGTCGC 430

             510       520       530       540       550
        ....|....|....|....|....|....|....|....|....|....|
E multi     ACATTCCCTACTGTGTCCCA---TGGACC--------------------- 471
T solium    AAGCTCTCTCTCCTGTTCTCAGATGTAGAATGTGCACGACGCCATTCGAT 510
T asiatica  AAGCTCTATCCCCTGTTCTCAGATGCAGAATACGCAGGATGCCATTGGAT 513
T taeniaef  AATTTCTTCCACAAAATCCG---TGTTTA--------------------- 456

             560       570       580       590       600
        ....|....|....|....|....|....|....|....|....|....|
E multi     ----------TGCATTGA------------ATCCCCTCCTTCCTTAC--- 496
T solium    AGCATCGTTTCATGCTTTGTACACTTGGATCACTTGCATCCCGTTCCTAT 560
T asiatica  AGCATCGTTTTATGCTTTGCACGCTTGGATCGCATGCCTTTTGTTCCTAT 563
T taeniaef  ----------GGTGTTGGGAAT-----GACACCACGGTTCTCCTGAC--- 488

             610       620       630       640       650
        ....|....|....|....|....|....|....|....|....|....|
E multi     --------------ACA---AATCTACTTTTGGG------TCGGTGTTCC 523
T solium    GAGGCCCGGTTGCCCCATTCGTTCAGGGTTCTAAGATACCCTTGCGTTTC 610
T asiatica  GAGGCCCGACTGCCACATTTGCTCAGGTTTCTACGATGCCCTTGCGTTTC 613
T taeniaef  --------------ATACT-GGACAGTGGTCCGC------CCCGTTTCTC 517

             660       670       680       690       700
        ....|....|....|....|....|....|....|....|....|....|
E multi     TTCATCCATCTATGGCCACGCATGATGGTTGATG--CAATTCCCCGT--- 568
T solium    TTCCACGGCAT-CAGAAAAGCCTGGAATCACAATATGTGTTGTGTGT--G 657
T asiatica  TTCTGCGGTAT-CAATTAAGCCTGGAATCGCAATATGTGTTGTGTGT--G 660
T taeniaef  CTGTAATATATATGATCTAGACCAGAGTTCAACCGTCTATTCCCGGT--- 564
```

```
                 710       720       730       740       750
             ....|....|....|....|....|....|....|....|....|....|
E multi      -CCTGAATCAAATTCCTAAACTCCTTGAA------------ACTCCAGAC 605
T solium     CCTTCATTCGTC-CAAAAACTTGGATTCGTATCCCCATCTTTCACTGGGC 706
T asiatica   TCACGACCCATT-TGAAAACTTGGATTCGTACCTCCATCTTTTACTGGAC 709
T taeniaef   -AGCAATTGACGATCAACGGGTGTATGCA------------GCTCCAAGT 601

                 760       770       780       790       800
             ....|....|....|....|....|....|....|....|....|....|
E multi      CCAGT---C--AAGCCG--------TCATGAGGGATCAG----TGGCGTT 638
T solium     CGCATCATCGTGGGAG-AGGAGGACTAAGAAATGCGCATTTGCTGCACCG 755
T asiatica   CACATTGTCATGAGAG-AGGACGGCCAAGGAGCGCGCATTTGCTGCACTG 758
T taeniaef   CTGGTTGTC--AGACCGAACGTACCTCCCGAGCCAGTTGAAGCTGTCGAG 649

                 810       820       830       840       850
             ....|....|....|....|....|....|....|....|....|....|
E multi      GA----------GGCCAAGCATC--TCCTCAGT-TGTGCGCTA-AT--TT 672
T solium     AGTGCTACGCAGAATTGATGGTACTACAAGAAGACATGCGCTCGAATCTC 805
T asiatica   GGTGCTACGCGGAATTGATGGCACTACAGGAACACATGCGTTCGAATCTC 808
T taeniaef   GA----------GACCAAGTCCCCTTTCTCGGT-CCCATGCT--GT--CT 684

                 860       870       880       890       900
             ....|....|....|....|....|....|....|....|....|....|
E multi      GGTGACTGGCACTGTCGCATCGTGGGTGTTTCTTTACTGCGCCAACTACG 722
T solium     GAAGGCTGGCACGGTTAGCTCGTGGGTGCTTCTC-GCAGCACTGATTACG 854
T asiatica   GACGACTGGGTGGTTAGCTCGTGGGTGCTTCTC-GCAGCACTGATTACG 857
T taeniaef   ATCGACTGGCACGGTTGGTTCGTGGATGCTTTCTTGTAGTACCGAATATG 734

                 910       920       930       940       950
             ....|....|....|....|....|....|....|....|....|....|
E multi      TTGTAATGGCTGACGCGG-GAGGTTGACCTG-CGGAAACGCTGGGGACGA 770
T solium     TTGTGATGGCTGACACGG-GAGGCTGACCAA-GGGGAGCGTCGGGGACGA 902
T asiatica   TTGTGATGGCCAACACGG-GAGGCTTACCAA-GGTGAGCGTCGGAGACGA 905
T taeniaef   TTGTGATGGCTGGTGTGG-AAGGTGTACCAA-GGGGAGCGCCGAGGACGT 782

                 960  PPT  970       980       990      1000
             ....|....|....|....|....|....|....|....|....|....|
E multi      CGCTCAAAAGAGGAGGAGGCTATGTAATACGATG-ATGGTTTATCCCTC 819
T solium     CGCTCGAAAGAAGAGAGGCGCTATGTAATACTTT----GTTTGTTC---- 944
T asiatica   CGCTCGAAAGAAGAGAGGCGCTGTGTAATGCCTT----GAATGTTC---- 947
T taeniaef   CACTCAAAAGAAAAGAGGCACAGTGTAGTGCTTTGTATATGTGACCACAT 832

                 1010      1020      1030      1040      1050
             ....|....|....|....|....|....|....|....|....|....|
E multi      GGTATTGGTGAGGCGCCACTTTGGTACTTTGATTTAGCCCCTTTGTACCT 869
T solium     --------GAACTGCCGGTTTCGGTGACTTGGGT-GGCCATCTTGTACT- 984
T asiatica   --------GAACTACCGGTTTAGGTCACTTGGGT-GGCCATATTGTACTG 988
T taeniaef   GACGTTTGGCA---GTCACTTTAGAATTT--GTTTGGCCC--TTGTACTC 875
```

```
                    1060      1070      1080      1090      1100
                ....|....|....|....|....|....|....|....|....|....|
E multi       GTCTGGGTGGGCTGGCCTTTTGGGCCATGCCAATAGCC--CAGACACCTT 917
T solium      -AGCCGGTTGGCCGGCCTTT-GGGCCGTGCCAACAGCCCCCAAGCCCTCT 1032
T asiatica    -AGCCGGTTGATGGGCCTTT-GGGCCGTGCCAACAGCCCCCAAGCCCTCT 1036
T taeniaef    GT------GGGCTGGCCTTC-GGGCCATGCTCAGGTCCGTTTGTTTTTTT 918

                    1110      1120      1130      1140      1150
                ....|....|....|....|....|....|....|....|....|....|
E multi       CTCTGCACTGAATATGTATTCTGC-TCGAGTATT--GTTTCTTCTTTCTT 964
T solium      GTTTCCAGAATA--TATATTCTGCCCC-AATACTATGCCTTCT-TTTACC 1078
T asiatica    GTTTTCAGAATACATGCGTTCTACCCCCAGTGCTATGCCTTCT-TTTACC 1085
T taeniaef    CTCTTTGGAATATATCTATCCAGCCTCACTTACT--GCCTTCT-TTCACT 965

                    1160      1170
                ....|....|....|....|....|
E multi       CTTCAGGAAGTCTCGGGACGCTAC- 988
T solium      TTAAAGGGAGTCTCGGGACGCTACA 1103
T asiatica    TTAAAGGGAGTCTTGGGACGCTACA 1110
T taeniaef    CTATGGGTAGTCTCGTGGCGCTACA 990
```

The TRIM elements are taken from the following regions of the genomic assemblies:

E. multilocularis pathogen_EmW_Chr_02_292769_293756 (imperfect element, lacking 3´A in 3´LTR)

T. solium pathogen_TSM_contig_00196 115560 – 116662

T. asiatica TASK.contig.00727.42714 2702 – 1593

T. taeniaeformis TTAC.scaffold.04445.3608 184 – 1173 (imperfect element, substitution in PBS)

Supplementary Data 5. Distribution of *ta-TRIM*s in the genome of *E. multilocularis*. The figure displays graphically the distribution of *ta-TRIM*s within the chromosomes of *E. multilocularis*. The number of sequences showing similarity to the LTR region of *ta-TRIM*s of *E. multilocularis*, obtained through a combination of blast and HMM analyses, was counted and graphed for non-sliding windows of 100 kb in each chromosome. Total lengths (X-axis) for chromosomes 1 to 9 are 20116480, 17597483, 14622996, 13762453, 11859843, 7212196, 7437390, 6203120 and 4267854 bp, respectively.

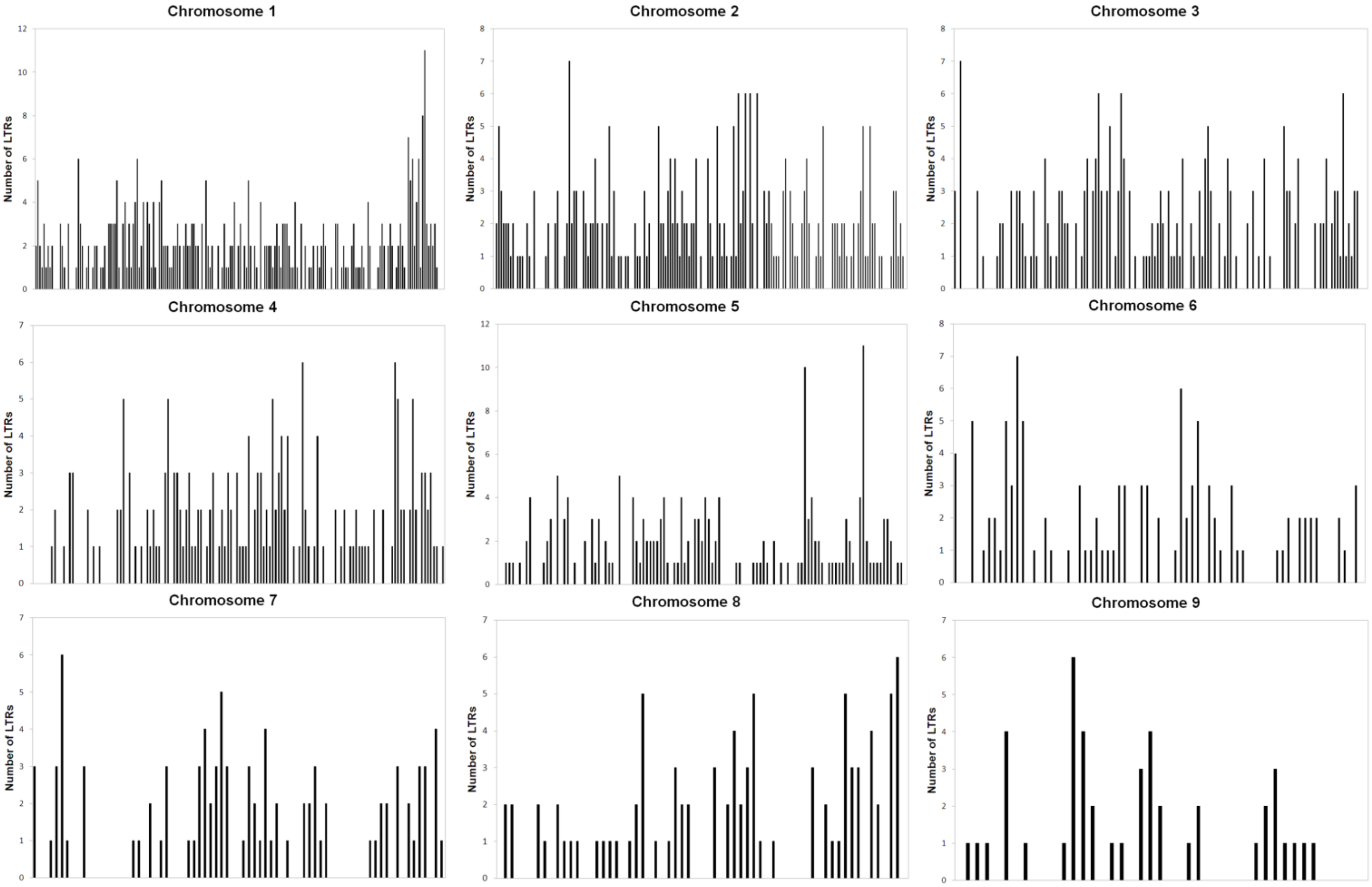

Supplementary Data 6. Examples of integrations of *ta-TRIM*s after the divergence of *Echinococcus* spp., and of *Taenia* spp. Alignment of a *ta-TRIM* element and its surrounding region from *E. multilocularis* with the ortholog region of *E. granulosus*, and of a *ta-TRIM* element and its surrounding region from *T. solium* with the ortholog region of *T. asiatica.*

Supplementary data 6A.

```
                          10        20        30        40        50        60        70        80
                  ....|....|....|....|....|....|....|....|....|....|....|....|....|....|....|....|
Em pathogen_EMU   GCATAAGTGTGCCCATTCTCGTCCTTTGTTACGTGGCTCTGCTCTTAGGTCATTGTTCCCAGGATTTGCTATGGACACGT
Eg pathogen_EgG   GCATAAGTGTGTTCATTCTCGTCCTGTGTTACATGGCTCTGCTCTTAGGTCATTGTTCCCAGGATTTGTTATGGACACGT

                          90       100       110       120       130       140       150       160
                  ....|....|....|....|....|....|....|....|....|....|....|....|....|....|....|....|
Em pathogen_EMU   GTGATAACCAGCTCATTGTCCCGTCTTCAATTCCAGTTCCCATACCCGACGAAACTCAAGGTCCACTTGGGTTGCCACGA
Eg pathogen_EgG   GTGATGACCAGCTCATTGTCCCGTCCTCAATTCCAGTTCCCATACCCGACGAAACTCAAGGTCCGCTTGGGTTGCCATGA

                         170       180       190       200       210       220       230       240
                  ....|....|....|....|....|....|....|....|....|....|....|....|....|....|....|....|
Em pathogen_EMU   GGAATCAACCACATTGCAGTCATATGTCTCCTCAGTTGCACAAAAATTCGGTGACTGGCACGGTTGGTTTGTGGGTGTTT
Eg pathogen_EgG   GGGATCAGCCACATTGCAGTCATATGTCTCCTCAGTTGCACAAAAATTCGGTGACTGGCACGGTTGGTTTGTGGGTGTTT

                         250       260       270       280       290       300       310       320
                  ....|....|....|....|....|....|....|....|....|....|....|....|....|....|....|....|
Em pathogen_EMU   TCTTTACAGTACCGATTACCTTGTGATGGCCTACGTAGGAGATCGGCCAGGGGGAGCACTGGGGATAACGCTCAAGAGGG
Eg pathogen_EgG   TCTTTACAGTACCGATTACCTTGTGATGGCCTACGTAGGAGATCGGCCAGGGGGAGCGCTGGGGATAACGCTCAAGAGAG

                         330       340       350       360       370       380       390       400
                  ....|....|....|....|....|....|....|....|....|....|....|....|....|....|....|....|
Em pathogen_EMU   GAGAGAAGCTGTGGAATACGTTAG-TTTTTAACCCTCAATTTTGGTAAGCGAAACGCTTTGGGTCACTGCCATTGTGCCT
Eg pathogen_EgG   GAGAGAAGCTGTGGAATACGTTAGCTTTTTAACCCTCGATTTTGGTAAGCGAAACGCTTTGGGTCGCTGCCATTGTGCCT

                         410       420       430       440       450       460       470       480
                  ....|....|....|....|....|....|....|....|....|....|....|....|....|....|....|....|
Em pathogen_EMU   TTTCAAGGTGGGCTAATTTCCAGGCCATGCTGGTAGGCCTATGCCCTACTGTATGTAGAATATTTACATTCTACCCTAGT
Eg pathogen_EgG   TTTCAAGGTGGGCTGATTTCCAGGCCATGCCGGTAGCCCCATGCCCTACTGTATGTAGAATATTTACATTCTACCCTAGT

                         490       500       510       520       530       540       550       560
                  ....|....|....|....|....|....|....|....|....|....|....|....|....|....|....|....|
Em pathogen_EMU   GTTATAGCTTCTTCGGTCTTTAGGAAAGCCTCAGGGCGCTACAATTATAACCACTCGTCATACTAAATTTAGTAATGTCG
Eg pathogen_EgG   GTTATAGCTTCTTCGGTCTTCAGGAAAGCCTCAGGGCGCTACAATTATAGCCACTCGTCATGCTAAATTTAGTAATGTTG

                         570       580       590       600       610       620       630       640
                  ....|....|....|....|....|....|....|....|....|....|....|....|....|....|....|....|
Em pathogen_EMU   TACTTCCAATTGATTGGGCTTGTTGACGCATTCTATTAAGGCTCTGCGTTTAAATCCGTATAGAACAACCACACCACCAA
Eg pathogen_EgG   TACTTCCAATTGATTGGGCTTGTTGACGCATTCTATTAAGGCTCCGCGTTTAAATCCGTATAGGACAACCACCCCACCAG

                         650       660       670       680       690       700       710       720
                  ....|....|....|....|....|....|....|....|....|....|....|....|....|....|....|....|
Em pathogen_EMU   AAGTGAACGCCTTAAAAACAGGTTTAGTTTTTGTAAAAGTCTTTCAGCGCATAATTTCTAAAAAGGCATTGCAACTTACG
Eg pathogen_EgG   AAGTGAACGCCTTAAAAATAGGTTTAGTTTTTGTAAAAGTCTTTCAGCGCATAATTTCTAAAAAGGCATTGCAACTTACG

                         730       740       750       760       770       780       790       800
                  ....|....|....|....|....|....|....|....|....|....|....|....|....|....|....|....|
Em pathogen_EMU   AATGACAATAAGAATCCTTTTTAGGCTTCCGAGTACTGCAAATATCCTTTTTGTTTTGCAAAATGAAATTCCGTTCATCT
Eg pathogen_EgG   AATGACAATAAGAATCCTTTTTAGGCTTCCGAGTACCGCGAATATCCTTTTTGTTTTGCCAAATGAAATTCCGTTCATCT

                         810       820       830       840       850       860       870       880
                  ....|....|....|....|....|....|....|....|....|....|....|....|....|....|....|....|
Em pathogen_EMU   CTTGGCTTCGTTGATATACTATTAACAATAAAATCGCTTGGAAAAGCTAGCAAATTTCACTAGTTAATTCAGCTGAAGGG
Eg pathogen_EgG   CTTGGCTTCGTTGATATACTATTAACAATAAAATCGCTTGGAAAAGCTAGCAAATTTCACTAGTTAATTCAGCGGAAGGG

                         890       900       910       920       930       940       950       960
                  ....|....|....|....|....|....|....|....|....|....|....|....|....|....|....|....|
Em pathogen_EMU   GATGCAATTACCCATCGTTGATTTGTTTGTTGGCATAGGAGAATAAGTAATGTTTTTTGGTACCTTCGCGTCACGAATTC
Eg pathogen_EgG   GATGCAATTACCCATCGTTGATTTGTTTGTTGGCATAGGAGAATAAGTGGTGTTTTTTTGTACCTTCGCATCACGATTTC

                         970       980       990      1000      1010      1020      1030      1040
                  ....|....|....|....|....|....|....|....|....|....|....|....|....|....|....|....|
Em pathogen_EMU   CAACGCAATTGAAATGCTCACCGTCTATTTGGGAAGGCTTTTAAGGTCACACAAAAAGTGACCCGAGGTCTTACCCATCC
Eg pathogen_EgG   CAACGCAATTGAAATGCTCATCGTCTATTTGGGAAGCCTTTTAAGGTCACACAAAAAGTAACTCGAGGTCTTACCCATCC

                        1050      1060      1070      1080      1090      1100      1110      1120
                  ....|....|....|....|....|....|....|....|....|....|....|....|....|....|....|....|
Em pathogen_EMU   CGTTCCCATCGGGTCAGATTCCTTGCTAAAGGCATCACAGGTCCATGACAAAGATGTTTGCTCTGCATTTAGAGCCTGTA
Eg pathogen_EgG   CGTTCCCATATGGTCAGATTCCTTGCTAAAGGCATCACAGGTCCATGACAAAGATGTTTGCTCTGCATTCAGAGCCTGTA
```

```
                              1130      1140      1150      1160      1170      1180      1190      1200
                      ....|....|....|....|....|....|....|....|....|....|....|....|....|....|....|....|
Em pathogen_EMU       GAGTAAACCCACATGTTTCAACTTCGGCTTCTCTTTCATCTACTGCCACAGTGTCTTCAACGTCTTTCTTAACAGCTATA
Eg pathogen_EgG       GATTAAACCCACATGTTTCAACTTCGGCTTCTTTTTCATCTACTGCCACAGTATCTTCAACGTCTTTCTTAACAGCTATA

                              1210      1220      1230      1240      1250      1260      1270      1280
                      ....|....|....|....|....|....|....|....|....|....|....|....|....|....|....|....|
Em pathogen_EMU       GGCAGAGCATCATCCGATACAGAAGATGTTGTGCTAACTGACTCCGATGGTATGTTTAATCTCGGAATGGGAATCAGACT
Eg pathogen_EgG       GGCGGAGCATCATCCGATACAGAAGACGTTGTGCTAACTGACTCCGATGGTATGTTTAACCTCGGAATGGGAATAAGACT

                              1290      1300      1310      1320      1330      1340      1350      1360
                      ....|....|....|....|....|....|....|....|....|....|....|....|....|....|....|....|
Em pathogen_EMU       TGGCTATAGGGTTAATTAGTGATATTTTTTATCACTCACATTCGCACTCATTGGCCGAGTCAAACCTCTTTCATTGAACT
Eg pathogen_EgG       TGGCTGTAGGGTTAATTGGTGATATTTTTTATCACTCACATTCGCACTCATTGGCCGAGTCAAACTTCTTTCATTAAACT

                              1370      1380      1390      1400      1410      1420      1430      1440
                      ....|....|....|....|....|....|....|....|....|....|....|....|....|....|....|....|
Em pathogen_EMU       GGCTAAAATTGTCTTTGAAAGTCCTCCTAGAACTTAGAAGAAGAATCAAATTAAAACTTGAATTTATTGAAATTTGATAT
Eg pathogen_EgG       GGCTATAATTGTCTTTGAAAGTCCTCCTAGAACTTAGAAGA---ATCAAATTTAAACTTGAATGTATTGAAATTTGATAT

                              1450      1460      1470      1480      1490      1500      1510      1520
                      ....|....|....|....|....|....|....|....|....|....|....|....|....|....|....|....|
Em pathogen_EMU       GTTTATGATTCTGTTTAACCATCTATTTCAACCTATAGTCTTTCCACTGGAATTACATAAACAACTACCGTAAGTTAATA
Eg pathogen_EgG       GTTTATGATTCTGTTTAACCATTTATTTCAACCTAAAGCCTTTCCACTGGAATTACATAAACAACTACCGTAAGTTAATA

                              1530      1540      1550      1560      1570      1580      1590      1600
                      ....|....|....|....|....|....|....|....|....|....|....|....|....|....|....|....|
Em pathogen_EMU       CATATAATTTTACGTTAACTACTAGTTAAGGCTTGATTCATATTAGTTAATTTGGAATAAAGTATCTAATTAAAGTCACA
Eg pathogen_EgG       TATATAATTTCACGTTAACTACTAGTTAAGGCTTGATTCATATTAGTTAATTTGGAATAAAGTATCTAATTAAAGTCATA

                              1610      1620      1630      1640      1650      1660      1670      1680
                      ....|....|....|....|....|....|....|....|....|....|....|....|....|....|....|....|
Em pathogen_EMU       CCACTTAGCTGAAACGGATATCCCCATTGATGCGTTGTTCGGTAACTCAAACTATTTCCAAAAGAACATACCTAAGCGGT
Eg pathogen_EgG       CCATTTAGCTGAAACGGATATCCCCATTGATGCGTTGTTCGGTAACTCAAACTATTTCCAAAAGAACATACCTAAGCGGT

                              1690      1700      1710      1720      1730      1740      1750      1760
                      ....|....|....|....|....|....|....|....|....|....|....|....|....|....|....|....|
Em pathogen_EMU       CGTGAAAACACATTGGTGAATGAAAACAAACTACAATGGCTTACCAATGATGTGCCAAGAATTACGGTGATTGTGCTTGT
Eg pathogen_EgG       CATGAAAACACATTGGTGAATGAAAACAAACCACAATGGCTTGCCAAGAATGTGCCAAGAATTACGGTGATTGTGCTTGT

                              1770      1780      1790      1800      1810      1820      1830      1840
                      ....|....|....|....|....|....|....|....|....|....|....|....|....|....|....|....|
Em pathogen_EMU       GTCCACTTGGTGACACTGGTAAACATGTGAATGCTATCTCACACATTCCTTTCCTCGAAAGAGCCAATAGAAACGCCCAT
Eg pathogen_EgG       GTCCACTTGGTGACACTGGGAAACATGTGAATGCTATCTCACACATTCCTTTGCTCGAAAGAGCCAATAGAAACGCGCAT

                              1850      1860      1870      1880      1890      1900      1910      1920
                      ....|....|....|....|....|....|....|....|....|....|....|....|....|....|....|....|
Em pathogen_EMU       TTAAATGTTGTCCTGTGTGAAGACAAATAATGAACGATCCTAACAACGCCTAACAACGTTCATACTCCTCATCAACGAAT
Eg pathogen_EgG       TTAAATGTTGTCCTGTGTGAAGACAAATAATGAACGATCCTAACAACGCCTAACAACGTTCATACTCCTCATCAACGAAT

                              1930      1940      1950      1960      1970      1980      1990      2000
                      ....|....|....|....|....|....|....|....|....|....|....|....|....|....|....|....|
Em pathogen_EMU       TAAAATGTATTCCTAACTCAGTTCCGCTGTAAAGCCGGGTTTTGACCTTAGTTTGATCAGCAAATCTCATTGCGGCAATT
Eg pathogen_EgG       TAAAATGTGTTCCCAACTCAGTTCCACTGTAAAGCCGGGTTCGGACCTTAGTTTGGTCAGCAAATCTCATTGAAGCAATT

                              2010      2020      2030      2040      2050      2060      2070      2080
                      ....|....|....|....|....|....|....|....|....|....|....|....|....|....|....|....|
Em pathogen_EMU       TTAGCCCTATCTGTAACTCACTAGGCCTTTTGAACAAAAAAGGAATGGGACTATGAGTACCTTGTCGAAATCCGACTGTT
Eg pathogen_EgG       TTAGCCCTATCTGTAACTCACTAGGCCTTTTGAACAAAAAAGGAATGGGACT-TGAGTACCTTGTCGAAATCCGACTGTT

                              2090      2100      2110      2120      2130      2140      2150      2160
                      ....|....|....|....|....|....|....|....|....|....|....|....|....|....|....|....|
Em pathogen_EMU       TCTACCACTTTAGGGAACTTGCGGCTAGATTTTGTACC------------TTCGCAAATTCAAATAAGCCAGGCTAGTGA
Eg pathogen_EgG       TCTACCACTTTAGGGAACTTGCGGCTAGACTTCGTACCAGAGCCAGTACCTTCGCGAACTCAAACAAGCCGAGCTAGTGA

                              2170      2180      2190      2200      2210      2220      2230      2240
                      ....|....|....|....|....|....|....|....|....|....|....|....|....|....|....|....|
Em pathogen_EMU       GTCTAACTGCCACCGAAGCGCCTCAACCCAGAGGCACCTACCATATGATATCAGTAAGCACCTTAAAGCGCCTTCAGTAG
Eg pathogen_EgG       GTCTAACTGCCACCGAAGCGCCTTAACCCAAAGGCACCTACCATACGATATCAGTAAGCACCTTAAAGCGCCTTCAGTAG
```

```
                         2250      2260      2270      2280      2290      2300      2310      2320
                 ....|....|....|....|....|....|....|....|....|....|....|....|....|....|....|....|
Em pathogen_EMU  CCCCACTGACCAATCCAAACAATGACCCAACAACCATGTCATTAGGCCACAAAATACTCGTTCAAAATGGCCCCTCTGAA
Eg pathogen_EgG  CCCCACTGACCAATCCAAACAATGACCCAACAACCATGTCATTAGGCCACAAAATACTCGTTCACAATGGCTTCTCTGAA

                         2330      2340      2350      2360      2370      2380      2390      2400
                 ....|....|....|....|....|....|....|....|....|....|....|....|....|....|....|....|
Em pathogen_EMU  CTCAATACTCAGTTAGACTCATGTAAATGAGCGAAAGTGCCCGGCCAGAGGGCAGTTGGTACTGAGAATCGAGAACGAAA
Eg pathogen_EgG  CTCAATACTCAGTTAGACTCATGTAAATGAGCGAAAGTGCCCGGCCAGAGGGCAGTCGGTAATGAAAATCGAGAACGAAA

                         2410      2420      2430      2440      2450      2460      2470      2480
                 ....|....|....|....|....|....|....|....|....|....|....|....|....|....|....|....|
Em pathogen_EMU  ATAACCCACGGGAGGAAGGTGGATTCAGTGCAGCAGAAACCAACTACACGTCCCTTAAGAATTGGTCAGAACGAGATCCA
Eg pathogen_EgG  ATAACCCATGGGAGGAAGGTGGATTCAGTGAAGCAGAAACCAACTACATGTCCCTTAAGAATTGGTTAGAACGAGATCCA

                         2490      2500      2510      2520      2530      2540      2550      2560
                 ....|....|....|....|....|....|....|....|....|....|....|....|....|....|....|....|
Em pathogen_EMU  ACTTGTACTCTACCCTGCATTCGAGTTCCCAAAAACCCGCAAGCTTGAAAACTGTAATTAAAATGACCAAACAGTCGACT
Eg pathogen_EgG  ACTTGTACTTTACCCTGCATTCGAGTTCCTAAAAACCTACAAGCTCGAAAACTGTAATTAAAATGACCAAACAGCCGACT

                         2570      2580      2590      2600      2610      2620      2630      2640
                 ....|....|....|....|....|....|....|....|....|....|....|....|....|....|....|....|
Em pathogen_EMU  TAGGTGGGCGTAGTTACATTGCTACCGCCGCAAACACCCAGTAAGGTTTGAAATAAAGTAGAACATCGTAATAACTCGTA
Eg pathogen_EgG  TAGGTGGGCGTAGTTACATCGCTACCGCCGCAAATACCCGGTAAGGTTTGAAATAAAGTAGGACATAGTAATAACTCGTA

                         2650      2660      2670      2680      2690      2700      2710      2720
                 ....|....|....|....|....|....|....|....|....|....|....|....|....|....|....|....|
Em pathogen_EMU  CCCTATCAAAGAACTATGCACAATCCTGTTAATTTGAAGCCCCAATATAGGTAAAACTGTTCTAAATGTGAAGTACGTAG
Eg pathogen_EgG  CCCTGTCAAGGAACTATGCACAATCCTGTTTATTTGAGGCCCCAATACAGGTAAAACTGTTCTAAATGTGAAGTACGTAG

                         2730      2740      2750      2760      2770      2780      2790      2800
                 ....|....|....|....|....|....|....|....|....|....|....|....|....|....|....|....|
Em pathogen_EMU  AGCAACGAAGTACTAATAAATATCTGCTTATCTATTTTATCGAGTGATTTTAGGCTGTACATACAGGAGTTCAAAAGAAA
Eg pathogen_EgG  AGCAAAGAAGTAATAATAAATATCTGCTTATCTATTTTATCGAGTGATTTTAGGCTGTACATACAGGAGTTTAAAAGAAA

                         2810      2820      2830      2840      2850      2860      2870      2880
                 ....|....|....|....|....|....|....|....|....|....|....|....|....|....|....|....|
Em pathogen_EMU  AGGAATATCATAGCTTTGAGATAATCTCCTCCGGACTACTTCCAGCTTTATGAAGTTGTCGAACCTGGTCTGAATAACGT
Eg pathogen_EgG  AGGAACATCATAGCTTTGAGATAATCTCCTCCGGACTACTTCCAGCTTTATGAAGTTGTCGAACCTGGTCTGAATAACGT

                         2890      2900      2910      2920      2930      2940      2950      2960
                 ....|....|....|....|....|....|....|....|....|....|....|....|....|....|....|....|
Em pathogen_EMU  ATTTCAAAAAGGGATTCTGTAGTAAGCGGGGGTTCATTGATGACCGCAGCCTGCCATGAAACATAAGGCTTCAACTCACA
Eg pathogen_EgG  ATTTCAAAAAGGGATTCTGTAGTAAGCGGGGGTTCATTGATGACCGCAGCCTGCCATGAAACATAAGGCTTCAACTCACA

                         2970      2980      2990      3000      3010      3020      3030      3040
                 ....|....|....|....|....|....|....|....|....|....|....|....|....|....|....|....|
Em pathogen_EMU  TCTGTCCAGATTCTCTGGAACGACAAACTCCACACGCATTTCTGGCATCTCCTGAATCTCACCATTGACAACACATGACG
Eg pathogen_EgG  TCTATCCAGATTCTCTGGAACGACAAACTCCACACGCATTTCTGGCATCTCCTGAACCTCACCATTGACAACACATGATG

                         3050      3060      3070      3080      3090      3100      3110      3120
                 ....|....|....|....|....|....|....|....|....|....|....|....|....|....|....|....|
Em pathogen_EMU  TTGGGGGTTCCCTGAAAATAGTGACTGTGATTCTACAGGACAAACCTTGGAATCCAAGGATACCTAGCCCTTCGACGACT
Eg pathogen_EgG  TTGGGGGTTCCCTGAAAATAGTGACTGTGATTCTACAGGACAAACCTTGGAATCCAAGGATACCTAGCCCTTCGACGACT

                         3130      3140      3150      3160      3170      3180      3190      3200
                 ....|....|....|....|....|....|....|....|....|....|....|....|....|....|....|....|
Em pathogen_EMU  TTTGCAAACGTCGAGCAGCCATTTATATCGTTGAACGTATGCAATTTTTTGTCTGGATAAAACCAAGGATGTAGAAATAC
Eg pathogen_EgG  TTTGCAAACGTCGAGCAGCCATTTATATCGTTGAACGTATGCAATTTTTTGTCTGGATAAAACCGAGGATGTAGAAATAC

                         3210      3220      3230      3240      3250      3260      3270      3280
                 ....|....|....|....|....|....|....|....|....|....|....|....|....|....|....|....|
Em pathogen_EMU  CCCTGCACAAATGACCAACCAGGCACAACTTAATTGCCATACCAACGAAGGCTATTTAACAACCTTTTTATATAACGCAC
Eg pathogen_EgG  CCCTGCACAAATGACCAACCAGGCACAACTTAATTACCATCCCAACGAAGGCTCTTTACCAACCTTTTTATATAACGCGC

                         3290      3300      3310      3320      3330      3340      3350      3360
                 ....|....|....|....|....|....|....|....|....|....|....|....|....|....|....|....|
Em pathogen_EMU  CTTCACCTTTGCAATATGGTACACTTGTTTTCTCAGAAATTATTATTACCGTATAAAATCTTTTTTGTTTCTTTTCCATT
Eg pathogen_EgG  CTTTACCTTTGCAATATGGTACACTTGTTTTCTCAGAAATTATTTTTACCGTATACAATCTTTTTTGTTT-TTTTCCATT

                         3370      3380      3390      3400      3410      3420      3430      3440
                 ....|....|....|....|....|....|....|....|....|....|....|....|....|....|....|....|
Em pathogen_EMU  ATACTTTATGCAAGGTAGAATGTTGTTTTAAACTAGAGTTAGCACTCTAATTGCTATTTGGTAATGTGCGGTCTAGTTTA
Eg pathogen_EgG  ATATTTTATACAAGGTAGAATGTTGTTTTGAACTAGAGTTAGCACTCTAATTGCTATTTGGTAATATGCAGTCTAGTTTA
```

```
                     3450      3460      3470      3480      3490      3500      3510      3520
                 ....|....|....|....|....|....|....|....|....|....|....|....|....|....|....|....|
Em pathogen_EMU  GGAACTTGACCATCTAAGTTCTAAATGGCCTATTACTCGCTCCTGTTTGGAAAGCTATGTCAATGCATCCAATTGGCATA
Eg pathogen_EgG  GAAACTTGACCATCTAAGTCCTAAATGGCCTATTACTCGCTCCTGTTTGGAAAGCTATGTCAGCGCATCCAATTAGCATA

                     3530      3540      3550      3560      3570      3580      3590      3600
                 ....|....|....|....|....|....|....|....|....|....|....|....|....|....|....|....|
Em pathogen_EMU  AAAATGCTAAATCTTGCTTATACAAAATAAATATCTCCTGAAGGATTCGTTGCGTAATCTTTGAAAAACAGTACATCCAA
Eg pathogen_EgG  AAAATGCTAAATCCTGCTTAAACAAAATAAATATCTCCTGAAGGATTCGTTGCATAATCTTCAAAAAACAGTACATCCAA

                     3610      3620      3630      3640      3650      3660      3670      3680
                 ....|....|....|....|....|....|....|....|....|....|....|....|....|....|....|....|
Em pathogen_EMU  ATCCTTTCTAGTGGTTTTTAGTTTAGCCAAAATGACTCACTTTTAAGAAGATAAATCATCTAATTTGTATCAATTTTATA
Eg pathogen_EgG  ATCCTTTCTAGTGGTTTTTAGTTTAGCCAAAATGACTCACTTTTACGAAGATAACTCATCTAATTTGTAGCAATTTTATG

                     3690      3700      3710      3720      3730      3740      3750      3760
                 ....|....|....|....|....|....|....|....|....|....|....|....|....|....|....|....|
Em pathogen_EMU  AATTATGGGAGACGGTGCCCCCTAAATCATCATTAATAATGTCTTAGCAAACTACCGACGAAGCCTTCAAAATGGTCAGA
Eg pathogen_EgG  AACTACGGAAGACGGTGCCCTCTAAATCATCATTAATAATGTCTTAGCAAACTATTGACAAAGCCTTCAAAATGGTCAGA

                     3770      3780      3790      3800      3810      3820      3830      3840
                 ....|....|....|....|....|....|....|....|....|....|....|....|....|....|....|....|
Em pathogen_EMU  AATGGTATTTAACTAAATCACTTCTGTTGTTCTAGAAAATTTAACTATTTTACGATAATCTCTTTTGACAATAAGTCTTA
Eg pathogen_EgG  AATGGTATTTAACTAAATCACTACTGTTGTTCTAGAAAATTTAACTATTTTACGATAATCACTTTTGACAATCTGTCTTA

                     3850      3860      3870      3880      3890      3900      3910      3920
                 ....|....|....|....|....|....|....|....|....|....|....|....|....|....|....|....|
Em pathogen_EMU  CATCTGTGCAC-GTTTGAAATTTCAACCACCCAATTACTATTTTTGCTGACCCGTAAATATTATGTAATACGATAGTGGT
Eg pathogen_EgG  CATCTCTGCACCGTTTGAAATTTCAATCACATAATTACTATTTTTGCTGACCCGTAACTATTA-----------------

                     3930      3940      3950      3960      3970      3980      3990      4000
                 ....|....|....|....|....|....|....|....|....|....|....|....|....|....|....|....|
Em pathogen_EMU  TTATCCCTCGATTCTGGTGAGGCGCCACTTTTGTACTTTGGATTAGCCCCCTTGGGCCTCGCTGGGTGGTCTGGCCTTTT
Eg pathogen_EgG  --------------------------------------------------------------------------------

                     4010      4020      4030      4040      4050      4060      4070      4080
                 ....|....|....|....|....|....|....|....|....|....|....|....|....|....|....|....|
Em pathogen_EMU  GGGCCATGCCAATAGCCCAGACGCCTTCTCCGTGCTGAGTATATATTCTGCCCGAGTATTGTGTCTTCTTTCGTCTTCAG
Eg pathogen_EgG  --------------------------------------------------------------------------------

                     4090      4100      4110      4120      4130      4140      4150      4160
                 ....|....|....|....|....|....|....|....|....|....|....|....|....|....|....|....|
Em pathogen_EMU  AGAGGCTCGGGGCGCTACATTGGTGGCAGCGGAAAGCGAGCCCAATCTTAAAAGGCATGTATCCCGACTTTCCACGTCTG
Eg pathogen_EgG  --------------------------------------------------------------------------------

                     4170      4180      4190      4200      4210      4220      4230      4240
                 ....|....|....|....|....|....|....|....|....|....|....|....|....|....|....|....|
Em pathogen_EMU  CGTGATAAGCCTTACTGGCACGGAAGAACATATGCTACGGATGGGAGATTGGTGATGATCCCTAATAGAAGAGTTGATGT
Eg pathogen_EgG  --------------------------------------------------------------------------------

                     4250      4260      4270      4280      4290      4300      4310      4320
                 ....|....|....|....|....|....|....|....|....|....|....|....|....|....|....|....|
Em pathogen_EMU  TGGTGTCCACCTTCGGCATCTCTCCCAGTCGCGTGTTCTGCAGAAAAGCTATTAACAGCCGAAGCGACACTAGCCGTGAA
Eg pathogen_EgG  --------------------------------------------------------------------------------

                     4330      4340      4350      4360      4370      4380      4390      4400
                 ....|....|....|....|....|....|....|....|....|....|....|....|....|....|....|....|
Em pathogen_EMU  CCATCTGGAAGTTTCGTTGCGGGTTTCCCCTACCGTCCTCCATGGACCTGTTTTAAATCTTGTATTCCCTTATTCAAATC
Eg pathogen_EgG  --------------------------------------------------------------------------------

                     4410      4420      4430      4440      4450      4460      4470      4480
                 ....|....|....|....|....|....|....|....|....|....|....|....|....|....|....|....|
Em pathogen_EMU  TGCTTTTAGGCCAGTGTTCCTGCATCTATCTATGGACACGCATGATGGCCGGCGCAGTTCCTCGTCCTAAATGAAATCCC
Eg pathogen_EgG  --------------------------------------------------------------------------------

                     4490      4500      4510      4520      4530      4540      4550      4560
                 ....|....|....|....|....|....|....|....|....|....|....|....|....|....|....|....|
Em pathogen_EMU  TAAACCGGACGAAACCCCAGACCCAGTCAAGCTGCCAAGAGGGATCAATGACGTTGAGGTCAAGTATCCTCTCAGTTGCG
Eg pathogen_EgG  --------------------------------------------------------------------------------

                     4570      4580      4590      4600      4610      4620      4630      4640
                 ....|....|....|....|....|....|....|....|....|....|....|....|....|....|....|....|
Em pathogen_EMU  CGCTAATTTGGTGACTGGCACGGTTGGCTCGTGGGTGTTTCTTTACTGCACCGACTACGTTGTAACGGCTGACGCGGGAG
Eg pathogen_EgG  --------------------------------------------------------------------------------
```

```
                         4650      4660      4670      4680      4690      4700      4710      4720
                    ....|....|....|....|....|....|....|....|....|....|....|....|....|....|....|....|
Em pathogen_EMU     GTTGACCTGTGGGAGCGCCGGGGACGACGCTTAAAAGAGGAGAGAGGCTATGTAATAGGATAGTGGTTTATCCATCGATT
Eg pathogen_EgG     --------------------------------------------------------------------------------

                         4730      4740      4750      4760      4770      4780      4790      4800
                    ....|....|....|....|....|....|....|....|....|....|....|....|....|....|....|....|
Em pathogen_EMU     CTGGTGAGGCGCCACTTTTGTACTTTTGTACCTCGCTGGGTGGGCTGGCATTTTGGGCCATCCTAATAGCCCAGACGCCT
Eg pathogen_EgG     --------------------------------------------------------------------------------

                         4810      4820      4830      4840      4850      4860      4870      4880
                    ....|....|....|....|....|....|....|....|....|....|....|....|....|....|....|....|
Em pathogen_EMU     TCTCTGTGCCGAATATATATATATATATATATATATTCTGCCCGAGTATTGTGTCTTCTTTTGTCTTCAGGGAGTCCCGG
Eg pathogen_EgG     --------------------------------------------------------------------------------

                         4890      4900      4910      4920      4930      4940      4950      4960
                    ....|....|....|....|....|....|....|....|....|....|....|....|....|....|....|....|
Em pathogen_EMU     GACGCTACAATTATCTGTAATTCCGTAGGAATATCTCCTTCTTTTTGAGGAAGTAGTTGGTCT-TTTCTTTTATAAATTG
Eg pathogen_EgG     -------------TCTGTAGTTCCGTAGGAATATCTCTTTCTTTT-GGGGAAGTAGTTAATCTGTTTTTTTTATAAATTG

                         4970      4980      4990      5000      5010      5020      5030      5040
                    ....|....|....|....|....|....|....|....|....|....|....|....|....|....|....|....|
Em pathogen_EMU     ATATTCTTTTACTATTTTTGAAGCGTTTGAATCAAGCTAACGCATGATAAAATTCCAGAACATTCACAAGAATCCTGAAG
Eg pathogen_EgG     ATATTCTTTTACTGGTTTTGAAGCGTTTGAATCAAGCTAACGCACAATAAAATGCCGGAACATTCACAAGAATCCTAAAG

                         5050      5060      5070      5080      5090      5100      5110      5120
                    ....|....|....|....|....|....|....|....|....|....|....|....|....|....|....|....|
Em pathogen_EMU     TGCATTAGCTCTACAGCCTGTTAGTTTAGCT-CTTTTAACAGTTCGCTTTAAATAATATTGTTTCAGATAAATATAGAGT
Eg pathogen_EgG     GGCATTAACTCTACAGCCTGTTAGTTTAGCTGCTTTTAACAGTTCGCTTTAAATAATATTGTTTCAGATAAATATAGAGT

                         5130      5140      5150      5160      5170      5180      5190      5200
                    ....|....|....|....|....|....|....|....|....|....|....|....|....|....|....|....|
Em pathogen_EMU     ATATTGTGGTATTTTTACTTAACTCTCACCTGTTTTCCTGTAACGCCGGAATAAATCGTCTTCGGTTGTTTTATGGTCCT
Eg pathogen_EgG     ATATTGTGATATTATTACTTAGCTCTCACCTGTTTTACTGTAAAACCGGAATAAATCGTCTTCAGTTGTTTTATGGTCCT

                         5210      5220      5230      5240      5250      5260      5270      5280
                    ....|....|....|....|....|....|....|....|....|....|....|....|....|....|....|....|
Em pathogen_EMU     TTAACTATTGCCGATCATCTTTTATTCACTACACATTCCACCTTTATTGTATGACCATAACAATTAACTTATGTATGGTT
Eg pathogen_EgG     TTAGCTATTGCCTATTATCTTTTATTCACTACACATTCCACTTTTATTGTATGACCATAACAATAAACTTATGTACGGTC

                         5290      5300      5310      5320      5330      5340      5350      5360
                    ....|....|....|....|....|....|....|....|....|....|....|....|....|....|....|....|
Em pathogen_EMU     TTGAGGAAACAGTCGAATTTTTTGACAGTCCCTGCAATCTGTTCCCCTAAAATATGATCTTGAACAATCAACAAATTAGG
Eg pathogen_EgG     TCGAGGAAACAGTCGAATTTTTTGACAGTCCCTGCAATCTGTTTCCCTAAAATATGATCTTGAACAATCAACAAGTTAGG

                         5370      5380      5390      5400      5410      5420      5430      5440
                    ....|....|....|....|....|....|....|....|....|....|....|....|....|....|....|....|
Em pathogen_EMU     AGTGGTTCAAATTCCGAAGAACAAATTTTGGTTTAAGAATACTTTGAGTCTGTTGGTTATTGGTTTAGGTGATCAGTATT
Eg pathogen_EgG     AGTGGTTCAAATTTAGAAGAACAAATTTTGGTTTAAGAATACTTTGAGTCTGTTGGTTATTGGTTTAGGTGATCAGTATT

                         5450      5460      5470      5480      5490      5500      5510      5520
                    ....|....|....|....|....|....|....|....|....|....|....|....|....|....|....|....|
Em pathogen_EMU     TGATGGATTCTATTTTTATGATTGTATAAAGCCAGTGCAAGGGATTTCGAAATGATACTGGTGGAATGCCCTCATTTACT
Eg pathogen_EgG     TGAAGGATTCTATTTTTATGATTGTATAAAGCCAGTGCAAGGGATTTCGAAATGATACCAATGGAATGCCCTCATTGACT

                         5530      5540      5550      5560      5570      5580      5590      5600
                    ....|....|....|....|....|....|....|....|....|....|....|....|....|....|....|....|
Em pathogen_EMU     CATTTGTGGGGATTTTCCTCAACTATATGGACCAGCAACCTAAAAATGTTTAGTACAAGACTCATGGTTGTTAAACGAAG
Eg pathogen_EgG     CATTTGTAGGGATTTTCCTCAACTATATGGACCAGCAACCTAAAAATGTTTAGTACAAGACCCATGGTTGTTAAACAAAG

                         5610      5620      5630      5640      5650      5660      5670      5680
                    ....|....|....|....|....|....|....|....|....|....|....|....|....|....|....|....|
Em pathogen_EMU     AGACTAATTTGCGATTTCTAATAGTTACCCTCACTTCGACTGAAGGAGGGT-----------------GTAGTGTAGCCA
Eg pathogen_EgG     AGACTAATTTGCGATTTCTAATAGTTACCCTCACTTCGACTGAAGGAGGGGCCATGGAAATCAAGTCAGTAGTGTAGCCA

                         5690      5700      5710      5720      5730      5740      5750      5760
                    ....|....|....|....|....|....|....|....|....|....|....|....|....|....|....|....|
Em pathogen_EMU     GCTCACACCCATTTCCTAACTTTGATATATTTGGCAATATTAAAAATATAGCATACCACTTCTCTTAAGAGCACAAAAGT
Eg pathogen_EgG     GCTTACACCCATTTCCTAACTTTGGTATATTTGGCAATATTAAGAATATAGCATACCACTTCTCTTAAGAGCACAAAAGT

                         5770      5780      5790      5800      5810      5820      5830      5840
                    ....|....|....|....|....|....|....|....|....|....|....|....|....|....|....|....|
Em pathogen_EMU     TGATTTGTGATTCTAAAGCAGGAAATAAGTAGACCTCTCAACGATTAATCATGTCGCAATTCCTCGACGCAC----CGTA
Eg pathogen_EgG     TGATTTGTGATTCTAAAGCAGGAAATAAGTAGACCTCTCAACGATTAATCATGTCGCAATTCCTCGACGCACTAACCGTA
```

```
                   5850      5860      5870      5880      5890      5900      5910      5920
               ....|....|....|....|....|....|....|....|....|....|....|....|....|....|....|....|
Em pathogen_EMU  TAGATAGAAAATAGAATTTATGACCATAGTCTTGCAATGTGTACAGTCGAACCACTTATTACATTTAGAGCTATTTATTA
Eg pathogen_EgG  TAGATAGAAAATCGAATTTATGACCATAGTCTTGCAATGTGTACAGTCGAACCACTTATTACATTTAGAGCTATTTATTA

                   5930      5940      5950      5960      5970      5980      5990      6000
               ....|....|....|....|....|....|....|....|....|....|....|....|....|....|....|....|
Em pathogen_EMU  TCCTGCTTCCTCGAGTGGTATAAATAAGGCTGAAGTGGGAGAGTTCAATCAGAATGCAATCTTCCTACTTCCGCATTCTA
Eg pathogen_EgG  TCCTTCTTCCTCGAGTGGTATAAATAAGGCTGAAGTGGGAGAGTTCAATCAGAATGCAATTTTCCTACTTCCGCATTCTA

                   6010      6020      6030      6040      6050      6060      6070      6080
               ....|....|....|....|....|....|....|....|....|....|....|....|....|....|....|....|
Em pathogen_EMU  CTCATCTTCCTCCTCACTCTAGTCTCTGTTGGAGCCAAAAGAAAGTGGAAGACGCTAATGAATGAGGATGATAGTGACAG
Eg pathogen_EgG  CTCATCTTTCTCCTCACTCTAGCCTCTGTTGGGGCCAAAAGAAAGAGAAAGACGATAATGAATGAGGATGATAGTGACAG

                   6090      6100      6110      6120      6130      6140      6150      6160
               ....|....|....|....|....|....|....|....|....|....|....|....|....|....|....|....|
Em pathogen_EMU  TGAGGTGGGTGAATGGAGAGCAGAAGGTAGGAGGCTCACAAGACTTGGATGCAGTTTTCGAAACACCTTTGCAAGGCACT
Eg pathogen_EgG  TGAGGTGGGTGAATGGAGAGCAGAAGGTCGGAGGCTCACAAGACCTGGATGCAGTTTCCGGAACACCATTGCAAGGCACT

                   6170      6180      6190      6200      6210      6220      6230      6240
               ....|....|....|....|....|....|....|....|....|....|....|....|....|....|....|....|
Em pathogen_EMU  GCACATGCATGCGGAGCGAATAGCCAAGGGTGAAGATGTAACTCGATCTTATATGCCCATTATCATCCACAGACAGGGTT
Eg pathogen_EgG  GCACATGCATGCGGAGAGAATAGCCAAGGGTGAAGATGTAACTCGATCTTATATGCCCATTATCATCCACAGACAGGGTT

                   6250      6260      6270      6280      6290      6300      6310      6320
               ....|....|....|....|....|....|....|....|....|....|....|....|....|....|....|....|
Em pathogen_EMU  AAGTAGTACTGCATGCTAAGTGTCACCTGTAATAAGTTAATGGGACTCTTCGCTTTTCTATTGTTTTGCTTTACATAACT
Eg pathogen_EgG  AAGTAGTACTGCATGCTAAGTGTCACCTGTAATAGGTTAATGGGACTCTTCGCTTTTCTATTGTTTTGCTTTACATAACT

                   6330      6340      6350      6360      6370      6380      6390      6400
               ....|....|....|....|....|....|....|....|....|....|....|....|....|....|....|....|
Em pathogen_EMU  TTTGTCCCCTGTGTACCTGTTTTACCGCACTGTTAAAGTACTTCCAACTAAACTTCTTGGTACATATTAAAGTGCTGGCG
Eg pathogen_EgG  TTTGTCCCCTGTGTACCTGTTTTACTACACTGTTAAAGTACTTCCAACTAAACTTCTTGGTACATATTAAAGTGTTGGCG

                   6410      6420      6430      6440      6450      6460      6470      6480
               ....|....|....|....|....|....|....|....|....|....|....|....|....|....|....|....|
Em pathogen_EMU  CCTGTTTCAAGTTACTGTGAAGTATGTAATAATTTATTTTAAGATTCCGATATTATAAAGAACTTGTTGTCCACTGCCTT
Eg pathogen_EgG  CCTGTTTCAAGTTACTGTGAAGTATGTAATAATTTATTTTACGATTCCGATATTATGAAGAACTTGTTGTCCACTGCCTT

                   6490      6500      6510      6520      6530      6540      6550      6560
               ....|....|....|....|....|....|....|....|....|....|....|....|....|....|....|....|
Em pathogen_EMU  ACTTCCTAAGAAAAGGTGGATGCGGGTTACTGAGCAAGGGTTGATAAACCCAAGAGACTACTATGATTGACACTGGTTAA
Eg pathogen_EgG  GCTTCCTAAGGAAAGGTGGATGCGGGTTACTGAGCAAGGGTTGAGAAACCCAAAAGACTACTATGGTTGACACTGGTTAA

                   6570      6580      6590      6600      6610      6620      6630      6640
               ....|....|....|....|....|....|....|....|....|....|....|....|....|....|....|....|
Em pathogen_EMU  ACAGGTATCTCCGCAAGTTCGATGTAATTCATTTTGCCTTGAAAAA-TAACATGGAGTATGTAAAAACTCATTGAATAAA
Eg pathogen_EgG  ACAGGTATCTCCGCAAGTTCGATGTAACTTATTTTGCCTTGAAAAAATAACATGGAGCATGTAAAAACTCATTAAATAAA

                   6650      6660      6670      6680      6690      6700      6710      6720
               ....|....|....|....|....|....|....|....|....|....|....|....|....|....|....|....|
Em pathogen_EMU  TAATATTCCTTGTTGAATTTTAAATAAAAAAATCAGATAAAGAGTTTACATGAGTGCATATTTGTTAATCACTGATGCAT
Eg pathogen_EgG  TAATATTCCTTGTTGAATTTTAAATAAAAAAATCAGATAAAGAGTTTACACGAGTGCATATTTGTTAATCACTAATGCAT

                   6730      6740      6750      6760      6770      6780      6790      6800
               ....|....|....|....|....|....|....|....|....|....|....|....|....|....|....|....|
Em pathogen_EMU  GAAGCATTTTTCACTAAAAAGATTTACATTAAGTTAGTTCAGGCTTCATTTGGTTTCATTTTGGTCGAATTCCGGGATTT
Eg pathogen_EgG  GAAGCATTTTTCACTAAAAAGATTTACATTAAGTTAGTTCAGGATTCATTTGGTTCCATTTTGGTCGAATTCCGGGATTT

                   6810      6820      6830      6840      6850      6860      6870      6880
               ....|....|....|....|....|....|....|....|....|....|....|....|....|....|....|....|
Em pathogen_EMU  GCAATATTAGAAGCACTCCAAGCTACTCCGTAGCTAAAGATGGGATTCTCAAAGTAGGGTGTTTTCATCCACGTGACCTC
Eg pathogen_EgG  GCAATATTAGAAGCACTCCAAACTACTCCGTAGCTGAAGATGGGATTCTCAAAGTAGGGTGTTTTCATCCACGTGACCTC

                   6890      6900      6910      6920      6930      6940      6950      6960
               ....|....|....|....|....|....|....|....|....|....|....|....|....|....|....|....|
Em pathogen_EMU  CTAGTTGTAGCAAGAGTCAAAAATATTCATTGTTGGTTTTCCAATTTTGATGGATTCTGCAGTAAGTAGCTTTAGTCGAA
Eg pathogen_EgG  CTAGTTGTAGCAAGATTCAAAAATATTCATTGTTGGTTTTCTAATTTTGATGGATTCTGCAGTAAGTGGCTATGGTCGAA

                   6970      6980      6990      7000      7010      7020      7030
               ....|....|....|....|....|....|....|....|....|....|....|....|....|....|....|....
Em pathogen_EMU  ACTGTAAACGCATCAAAAATGACATAATAATCTGCTTGAATCCGTCTTGTAGTCATTACAATTTACTAAGGCTACAAAA
Eg pathogen_EgG  ACTGTAAACGCATCAAAAATGACATCATAATCTGCTTGAATCCGTCTTGTAGCCATTACAATTTACTAAGG--------
```

Positions in assembly:

Em pathogen_EMU_scaffold_007780 5571000 to 5578000
Eg pathogen_EgG_scaffold_0002 5821533 to 5827567

Red: TSD
Blue: TRIM

Supplementary data 6B

```
                             10        20        30        40        50        60        70        80
                    ....|....|....|....|....|....|....|....|....|....|....|....|....|....|....|....|
Ts pathogen_TSM     TATGACATCCACTTCTTTCCATTTTCATCCACACATTCTCTTACCCTTTCTGCAGTTCACCTCTACTTTTGTTTACTTAC
Ta TASK.contig.     --TGACATCCATTTCTTTCCATTTTCATCTACACATTCTCTTACCCTTTCTACAGTTCATCTCTACTTTTGTTTATTTAC

                             90       100       110       120       130       140       150       160
                    ....|....|....|....|....|....|....|....|....|....|....|....|....|....|....|....|
Ts pathogen_TSM     CTTTTCTCACTTCTAAAATTTTATGTTCTAGGGCAACTTACCTTTTTCCTTCCAAATGGCGTGCTTTTTGGCGCCCCGTG
Ta TASK.contig.     TTTTTCTCATTTCTAAAATTTCATGTTTCAGGGCCACTTACCTTTTTCCTTCCAAATGGCGTGCTTTTTGGCGTCCCGTG

                            170       180       190       200       210       220       230       240
                    ....|....|....|....|....|....|....|....|....|....|....|....|....|....|....|....|
Ts pathogen_TSM     TCCTGTGAGTTCGTCCTTTACAAACGAAGCATTTAAATTAAGCAATTGAATGAACACCTTCTTTGTTTACTTCACCAAGT
Ta TASK.contig.     TCTTGTGAGTTCGTCCTTTACAAACGAAACAATTAAATTAAACAATTGAATGAACACCTTCTTTGTTTACTTCACTAAGT

                            250       260       270       280       290       300       310       320
                    ....|....|....|....|....|....|....|....|....|....|....|....|....|....|....|....|
Ts pathogen_TSM     CATGTGTGAAGCTTTAAGAAGCCTACCTCAGGTACAGGAAACTTCATCTTTTCTCCCAGTTTCTACAAAGTGCCAAACAA
Ta TASK.contig.     CATGTGTGCAGCTTTAAGAAGCCTACCTCAGGTACAGGGTACTTCATCTTTTCTCCCAGTTTCTAAAAAGTGCCAAACAA

                            330       340       350       360       370       380       390       400
                    ....|....|....|....|....|....|....|....|....|....|....|....|....|....|....|....|
Ts pathogen_TSM     ACATTATGGTCTGAATTGATTTAGAGTTT-GGTTATAAAATCATCTCTCCTTAAAAGCCTGTACTTTTGTAGATTGTTAA
Ta TASK.contig.     ACATTATGGTCTGAATTGATTTAGAGTTTTGATTATAAAATCATCTCTCCTTAAAAGCCTGTACTTTTATAGATTGTTAA

                            410       420       430       440       450       460       470       480
                    ....|....|....|....|....|....|....|....|....|....|....|....|....|....|....|....|
Ts pathogen_TSM     AGCGGTTTGGGATCGCAACTTCTGCTGGCTATTTGTTTGTAACTGTGTGAGGAACCACACATTTCGGTCATGAAACCAAT
Ta TASK.contig.     GGCGATTTGGGATGACAACTTTTGTTAACTGTTTGTTTGGAATTGTATTAGGAACCACATATATCGATCATGAAACCAAT

                            490       500       510       520       530       540       550       560
                    ....|....|....|....|....|....|....|....|....|....|....|....|....|....|....|....|
Ts pathogen_TSM     GCTATCTTCCGTAACTGCTAATTCTTTGTCTGCTATGTCGTTGTTTTAACATAAACTTGAATCACTGTTCTAATAAACAC
Ta TASK.contig.     GCTAGCTTCTGTAACGGTTAATTCTCCGTCTGCCATATCGTTGTCTTAAAATAAATTTGAACCACTTTTCGAGTAAACAA

                            570       580       590       600       610       620       630       640
                    ....|....|....|....|....|....|....|....|....|....|....|....|....|....|....|....|
Ts pathogen_TSM     CACTTTAGCTGTCGTCATCGGTCAAGGCAATTTTGGTAAGCACAACATGACAATAAATTAAACAGCTTTGGCAATTAATT
Ta TASK.contig.     CACTTTGGCTGTCGTCATCAGTCAAGGCGATTTCGGTAAGCACAACTTGACAATAAATTAAACAGCTTTGACAATTAATC

                            650       660       670       680       690       700       710       720
                    ....|....|....|....|....|....|....|....|....|....|....|....|....|....|....|....|
Ts pathogen_TSM     TAGCACATAAAAATGTGGTTTTTTGAACACATTCTTCCGTAGTGAAATGCTCTTACTAGGGGTCATTTTGCCTATTTTTG
Ta TASK.contig.     TAGCACACAAAAATGTGCTTTTTAGAACACATTCTTCCATAGTAAATTGCCCTTATTTAGGACCTATTTGCCTATTTTTG

                            730       740       750       760       770       780       790       800
                    ....|....|....|....|....|....|....|....|....|....|....|....|....|....|....|....|
Ts pathogen_TSM     ACGGCCACAGTTGTAACCTAACTCCTCGTAACAGTAAGTCATTTTTAATTCGATTTTTGTTCAAATAATGGGCGAGAACA
Ta TASK.contig.     ACGGCCAAAGTTGTAACCTAACTCCTTGTAACAGTAAGTCATTTTCAATTTGATATTTGTTCAAATAATGGGCGAGAACA

                            810       820       830       840       850       860       870       880
                    ....|....|....|....|....|....|....|....|....|....|....|....|....|....|....|....|
Ts pathogen_TSM     ACGACAGCCCTTCCATGGAATAGTCAACTACGCTACTGCATTTATCTCAGGCAAAAAGGAAGGGAGAGTCGTTTATAGTA
Ta TASK.contig.     ACGACAGCCCTTCCATGAAATAGTAGACTGTGCTACTACATTTATCTCAAGCAAAAAGGAAGGAAGAATCGTTTGTAGTA

                            890       900       910       920       930       940       950       960
                    ....|....|....|....|....|....|....|....|....|....|....|....|....|....|....|....|
Ts pathogen_TSM     GTGTTATGAGCCACCCTAAATTACAATTTAAAAATTTCCCAAACCTACTTT-AAAATATCCTCATGCCACTTAAGTTGTT
Ta TASK.contig.     GTGTTATGAGCTACCCTAAATTACAATTTAAAAATTCTCCAAACCTACTTTTAAAATGTCCTCATGCCACTTAAGTTATA

                            970       980       990      1000      1010      1020      1030      1040
                    ....|....|....|....|....|....|....|....|....|....|....|....|....|....|....|....|
Ts pathogen_TSM     CTTTC-GTGCAAAACTTACCCATGGAAGGAGCATCCATAGCTTATCTTTTTCTACCACTCCAGTGGCAAAGTTGGCCAGG
Ta TASK.contig.     CTTTTTGTGCAAAACTTGCCCATGGAAGGAGCATCCATAGCTTATCTTTTTCTACCACTCCAGTGGCAAAGTTGGCCAGG

                           1050      1060      1070      1080      1090      1100      1110      1120
                    ....|....|....|....|....|....|....|....|....|....|....|....|....|....|....|....|
Ts pathogen_TSM     GGTTTTACCACCGAGTTACCTTCCCTTCGAAAAGGCCTTTTTAATCCGTAGATAGGCACGGCAGTTCGGGGTAAAATCTG
Ta TASK.contig.     GGTTTCACCACCGAGTTGCCTTCCCTTCGAAAAGGCCTTTTTAATCCGTAGATAGGCACGGCAGTTCGGGGTAAAACCTG
```

```
                  1130      1140      1150      1160      1170      1180      1190      1200
                  ....|....|....|....|....|....|....|....|....|....|....|....|....|....|....|....|
Ts pathogen_TSM   CAACAAATTTACCAAAAACTTGATCACACCGTC----------------GTTAGGGCTTTTGCTGGTATTAAAAATTTCC
Ta TASK.contig.   CAACAAAGTTACCAAAAACTTGATCACACCAACAACACCAGAGCAAGATGTTAGGGCTTTTGCTGGTATTAAAAACTGTC

                  1210      1220      1230      1240      1250      1260      1270      1280
                  ....|....|....|....|....|....|....|....|....|....|....|....|....|....|....|....|
Ts pathogen_TSM   TTTAATGTAGGGACCTCTGGACAGTCATTAACAAGTTGGTTATTTGTTAAATTCTCGGTTTTTGGTAAGGCGGACCACTG
Ta TASK.contig.   CCTAAAGTAGGGCCCTCTAGACAGTCATTAACAAGTTGGTTACTTGTTTAATTCTCGGTTTTTGGTAAGGCGGAACACTG

                  1290      1300      1310      1320      1330      1340      1350      1360
                  ....|....|....|....|....|....|....|....|....|....|....|....|....|....|....|....|
Ts pathogen_TSM   TAGCTCTCCGCCATTGAACCTTTGCTCATGGGTCAGGCGTCTGAATCATGCCAGTAGCCTGAAGCCCTATTGTCCGAGTA
Ta TASK.contig.   TAGCTCTCCGCCATTGGACCTTTGCTCATGAAGCAGGCGTTCGTATCATGCCAGTAGTCTGAAGTCCTATTGCCCGAGTA

                  1370      1380      1390      1400      1410      1420      1430      1440
                  ....|....|....|....|....|....|....|....|....|....|....|....|....|....|....|....|
Ts pathogen_TSM   CTTGCGGCCTTTTTCGCCTTCAAGGAAGTCTTGAAGCGTCACAAGTCTGCACTTTTGTCTCGGTTTCTGCCAATTATGTG
Ta TASK.contig.   CTTGCGGCTTTTTTCGCCTGCATGGAAGTCTTGAAGCGTCACAAGTCTGCACCTCGGTCACAATTTCTGCCAATCATGTA

                  1450      1460      1470      1480      1490      1500      1510      1520
                  ....|....|....|....|....|....|....|....|....|....|....|....|....|....|....|....|
Ts pathogen_TSM   CTTCTTTGAGAAATGTAAAATGATTTTTCTGCATGTCCACCGAGTACATTGCATTCACCAAATTAGCCAGTTGTCACGGT
Ta TASK.contig.   CTTTTTTGAGAAATGTAAAATGATTTTTCTGCATGTCCACCGAGTACATTGCATTCACCAAATTAGCCAGTTGTCACGGA

                  1530      1540      1550      1560      1570      1580      1590      1600
                  ....|....|....|....|....|....|....|....|....|....|....|....|....|....|....|....|
Ts pathogen_TSM   -TTTGTTGGTGAAATAAGAACATAGTATTTACCGAGCAA------------TTTGGAAAGAAAGAAATATCGTGTCGAAG
Ta TASK.contig.   GTTTGTTGGTGAAATAAGAACATAGTAGTTACCGAGCAAGCTTGAATGCATTTTGGAAAGGAAGGAATATCGTGTCGAAG

                  1610      1620      1630      1640      1650      1660      1670      1680
                  ....|....|....|....|....|....|....|....|....|....|....|....|....|....|....|....|
Ts pathogen_TSM   GATAAGTTTTGAAATGAATTTTTTTGTGTGGCCAAGTCGAGCCTGCTGGAAGACACGCATGTAGACCGATTGGAGAATGC
Ta TASK.contig.   GATAAGTTTTTAAATGAATTTTTTTGTGTGGCCAAGTCGAGCCTGCTGGAAGACATGCATGTGGACCGATTGGAGAATGC

                  1690      1700      1710      1720      1730      1740      1750      1760
                  ....|....|....|....|....|....|....|....|....|....|....|....|....|....|....|....|
Ts pathogen_TSM   GGGCGGGTGCAGCACGAGTGCACGCACATATCCCCTGTGTTCCCCCTCAAGACGGTCAGTGAAGGGACCCAGGTCAGGCT
Ta TASK.contig.   GGGCGGGTGCAGCACGAG----CGCACACATCTCCCGTGTACCCCCTCAAGAAGGCCAGTGAAGGGACCCAGGTCAGGCT

                  1770      1780      1790      1800      1810      1820      1830      1840
                  ....|....|....|....|....|....|....|....|....|....|....|....|....|....|....|....|
Ts pathogen_TSM   GGACAACCACACCCAAGTGGCTGAGTCACAGTGTCTGTACCAGAGGTCAACAATCTGACCGATTCCAGACCAAGCGTGAC
Ta TASK.contig.   GGACAACCACACCCAAGCGGCTGAGTCACAGTGTCTGTACTAGAGGTCAGCAATCTGGCCGATTCCAGACCAAGCATGAG

                  1850      1860      1870      1880      1890      1900      1910      1920
                  ....|....|....|....|....|....|....|....|....|....|....|....|....|....|....|....|
Ts pathogen_TSM   AACATCGTGTGGCACAAGGTCATGCACATCGACGCTACAACAAATGGGCTCTCAGGTTCACTCCCTATA-AATTTTGTCT
Ta TASK.contig.   AACATCGTGTGGCACAATGCCATGCACATCGACGCTACAACAAATGGGCTCTCAGGTTCACTCCCTTTCTAATTTTGTCT

                  1930      1940      1950      1960      1970      1980      1990      2000
                  ....|....|....|....|....|....|....|....|....|....|....|....|....|....|....|....|
Ts pathogen_TSM   TTCCTTTTCCCTAGTTGCATCACGATTAGACAGGATTTTGCCTCCCAATTTAATCAAAATGAGTTAGTTTTTCTTTTCCA
Ta TASK.contig.   TTCCTTTTCCCTAGTTGCATCACTATTCGACAGAATTTTGCCTCCCTATTTTATCAAAATGAGTTAGTTTTTCTTTTTCA

                  2010      2020      2030      2040      2050      2060      2070      2080
                  ....|....|....|....|....|....|....|....|....|....|....|....|....|....|....|....|
Ts pathogen_TSM   TAGAGGCATCATGATATTTTACGTAGCGCCTGTCTAAATATTTTGACATTCACGAAATAGGTGATATTTTCAGGTCTTAA
Ta TASK.contig.   TAGAGGCATCATAATGTTTTACGTAGCGCCTGCCTAAATATTTTGACATTCACGAAATAGGTGATATTTTCAGGTCCTAA

                  2090      2100      2110      2120      2130      2140      2150      2160
                  ....|....|....|....|....|....|....|....|....|....|....|....|....|....|....|....|
Ts pathogen_TSM   AAACTTCCACTTCTACACTGAAAGAAGTGAACTTTTTTGGTTCAAATTTTTACACTCAAAACATGAAACTTGCGATTTGT
Ta TASK.contig.   AAACTTTCACTTCTACTCTGAAAGAAGTGAACTTTTTGGTTCAAATTTTTGCACTCAAAACATGCAACTTGCGATTTCT

                  2170      2180      2190      2200      2210      2220      2230      2240
                  ....|....|....|....|....|....|....|....|....|....|....|....|....|....|....|....|
Ts pathogen_TSM   TGTCAGAAAAGAGGTTGCCAAAAATAATTCCCAGATGGATGATGTCTGGTTTGACTACGTGTTTGGCTGGTTGGTACCAC
Ta TASK.contig.   TGTCAGAAAAGAGGTTGCCAAAAATAATCTCCAGATGGATGATGTCTGGTTTGACTACGTGTGTGGCTGGTTGGTATC--

                  2250      2260      2270      2280      2290      2300      2310      2320
                  ....|....|....|....|....|....|....|....|....|....|....|....|....|....|....|....|
Ts pathogen_TSM   CCTCGGTAGCTTGGCTTTTAGGTGTTTTGCCTCCAGACTAGGCGTGCTTCACGCCGCCTTCAGTGATTCTACATTAGCGA
Ta TASK.contig.   -------------------------------------------------------GCCTTCAGTGATTCTACATTAGCGG
```

```
                           2330      2340      2350      2360      2370      2380      2390      2400
                       ....|....|....|....|....|....|....|....|....|....|....|....|....|....|....|....|
Ts pathogen_TSM        CAAACATGGCTTTCGTCAGAGTAAGAAGACTGTGACTTATTAAAGAAGGGCTATCGCATCCGCGTCGACCCTCGAGTACC
Ta TASK.contig.        TAAACATGGCTTTCGTCGGAGGAAGAAGGCTGTGACCTATTAAAGAAGGGCTATCGCATCCGTGTCGACCCTTGAGTACC

                           2410      2420      2430      2440      2450      2460      2470      2480
                       ....|....|....|....|....|....|....|....|....|....|....|....|....|....|....|....|
Ts pathogen_TSM        ACGCCAATTCAGTCCCTCGCTCCTCTGTTCCTTAGTAAGTTTTGATGACTTCATGAAGTCTATGGCAGGT----------
Ta TASK.contig.        ACGCCAATTCAGTTCCTCACTTCTCTGTTCCTTAGTAAGCTTTGATGAATTAAATGAGGTCTATGGTAGGTCCAAGAGGAA

                           2490      2500      2510      2520      2530      2540      2550      2560
                       ....|....|....|....|....|....|....|....|....|....|....|....|....|....|....|....|
Ts pathogen_TSM        --------------GTAGTGATTTACCATTTTTCAAGATCACTTGGTCCAAGTAACCTTATGGTAGCTACCAATTCCGCC
Ta TASK.contig.        TTGCGATTTTCAGTGTAGTAATTTACCATTTTTTAAGATCACTTAGTCCCCGTAACCTCATGGCAGCTACTAATTCCGCC

                           2570      2580      2590      2600      2610      2620      2630      2640
                       ....|....|....|....|....|....|....|....|....|....|....|....|....|....|....|....|
Ts pathogen_TSM        ATGCCAGTAGAAAATGTTGCTAACGGCACTTTGGTCTGCTGCAACACCAAGGGAGTGCTCTGAGAGCCGCAGTCCAATCT
Ta TASK.contig.        ACGTCAGTAGAAAATGTTGCTAACGGTACTTTGGCCTGCTGCGACACCAAGGGAGTGCTCTGAGAGCCGCAGTCCAATCT

                           2650      2660      2670      2680      2690      2700      2710      2720
                       ....|....|....|....|....|....|....|....|....|....|....|....|....|....|....|....|
Ts pathogen_TSM        CGAAGACTTGATTATACATATTTCGGTAGAACTCAGTTCATTCTCATAGAGGCTGCAGAATTTAGGCGTGCATCGTCAAA
Ta TASK.contig.        CGAAGACTTGATTATACATATTTCAGTAGAACTTAGTTCATTCTTATACAGACCACAGAAGTTAGGAGTACATCGTCAAA

                           2730      2740      2750      2760      2770      2780      2790      2800
                       ....|....|....|....|....|....|....|....|....|....|....|....|....|....|....|....|
Ts pathogen_TSM        GGCTTCCACGAACCTAGTGCCTTCCTGTTCTACTTCTGAGCTAGGCAGCTTAGATACCTGGAGCGATTATCGAATGTCCG
Ta TASK.contig.        GGCTTCCACGAACCTAGTGCCTTCCTGTTCTACTTCTGAGCTAGGCATCTTAGACGCCTGAAGCGATTATCGAATGTCTG

                           2810      2820      2830      2840      2850      2860      2870      2880
                       ....|....|....|....|....|....|....|....|....|....|....|....|....|....|....|....|
Ts pathogen_TSM        TAAAGACCATAAAATTATCATTTTTAAAGGCGGTGTCAGAAGTATGGACAATCTTTGTAATACTTTGTTTGTTCGAACTG
Ta TASK.contig.        TAAAGACCATAAAATTGTCATTTTTAAAGGCGGTTTTAAACATATGGACAATCTT-------------------------

                           2890      2900      2910      2920      2930      2940      2950      2960
                       ....|....|....|....|....|....|....|....|....|....|....|....|....|....|....|....|
Ts pathogen_TSM        CCGATTTCGGTCACTTGGGTGGCCATATGGAACTAGCCGGTTGGCCGGCCTCCGGGCCGTGCCAACAGCCCCCAAGCCCT
Ta TASK.contig.        --------------------------------------------------------------------------------

                           2970      2980      2990      3000      3010      3020      3030      3040
                       ....|....|....|....|....|....|....|....|....|....|....|....|....|....|....|....|
Ts pathogen_TSM        CCGCCTCCAGAACATATATTCTGCCCTAATACTATGCCTTCTTTTTCCTTAAAGAGAGCCTCGGGACGCTACATTGGTGG
Ta TASK.contig.        --------------------------------------------------------------------------------

                           3050      3060      3070      3080      3090      3100      3110      3120
                       ....|....|....|....|....|....|....|....|....|....|....|....|....|....|....|....|
Ts pathogen_TSM        CAGCGGAAAGCGGACTCGACTTTGATGGATTTTATCCCGCTCCATGCCGCTTCTGTCAAACTTTGGCGGTGTGAAAGAAG
Ta TASK.contig.        --------------------------------------------------------------------------------

                           3130      3140      3150      3160      3170      3180      3190      3200
                       ....|....|....|....|....|....|....|....|....|....|....|....|....|....|....|....|
Ts pathogen_TSM        TGCATGTGCTACGGATGGGAGAATCCCTTACATCCCATTACAGAAGGGTTGAGATCGACATTCATCTTCGGCACCTCCCT
Ta TASK.contig.        --------------------------------------------------------------------------------

                           3210      3220      3230      3240      3250      3260      3270      3280
                       ....|....|....|....|....|....|....|....|....|....|....|....|....|....|....|....|
Ts pathogen_TSM        CGCCCTCGTGTTCGACAGGGAAGCTATTAACAACTGAAGCGACACCAGCCGTGAACCTTAAGTCCAGTGCAACTGCCAAG
Ta TASK.contig.        --------------------------------------------------------------------------------

                           3290      3300      3310      3320      3330      3340      3350      3360
                       ....|....|....|....|....|....|....|....|....|....|....|....|....|....|....|....|
Ts pathogen_TSM        GAGGATCAGCGACGTTGTGATCAAACATCCTCCGTAAGCTCTCTCTCCTGTTCTCAGATGTAGAATGTGCAGGACGCCAT
Ta TASK.contig.        --------------------------------------------------------------------------------

                           3370      3380      3390      3400      3410      3420      3430      3440
                       ....|....|....|....|....|....|....|....|....|....|....|....|....|....|....|....|
Ts pathogen_TSM        TCGATGGCATCGTTTTATGCTTTGTACACTTGGATCACGTGCATCCCGTTCCTATGAGGCCCGATTGCCCCATTCGCTCA
Ta TASK.contig.        --------------------------------------------------------------------------------

                           3450      3460      3470      3480      3490      3500      3510      3520
                       ....|....|....|....|....|....|....|....|....|....|....|....|....|....|....|....|
Ts pathogen_TSM        GGGTTCTAAGATACCCTCGCGTTTCTTCCACGGCATCAGAAAAGCCTGGAATCACAGCATGTGTTGTGTGTGTCTCTCAT
Ta TASK.contig.        --------------------------------------------------------------------------------
```

```
                         3530      3540      3550      3560      3570      3580      3590      3600
                ....|....|....|....|....|....|....|....|....|....|....|....|....|....|....|....|
Ts pathogen_TSM CCATTCAAAAACTTGGATTCGTATCCCCATCTTTCATTGGGCCACATTATCGTGGGAGAGGAGGACCAAGATATGCGCAT
Ta TASK.contig.  --------------------------------------------------------------------------------

                         3610      3620      3630      3640      3650      3660      3670      3680
                ....|....|....|....|....|....|....|....|....|....|....|....|....|....|....|....|
Ts pathogen_TSM CTGCTGCACCGAGTGCTACGCAGAGTTGACGGCACTACAAGAAGACATGCGCTCGAATCTCGATGGCTTGCACGGATAGC
Ta TASK.contig.  --------------------------------------------------------------------------------

                         3690      3700      3710      3720      3730      3740      3750      3760
                ....|....|....|....|....|....|....|....|....|....|....|....|....|....|....|....|
Ts pathogen_TSM TCGTGGGTGCTTCTCACAGCACTGATTACGTTGTGATGGCTGACACGTGAGGCTGACCAAGGGGAACGTCGGAGACGACC
Ta TASK.contig.  --------------------------------------------------------------------------------

                         3770      3780      3790      3800      3810      3820      3830      3840
                ....|....|....|....|....|....|....|....|....|....|....|....|....|....|....|....|
Ts pathogen_TSM CTCGAAAGAAGGGAGGGGCTGTGTAATACTTTGTCTGTTCGAACTGCCGGTTTCGGTCATTTGGGTGGTCATATTGTACT
Ta TASK.contig.  --------------------------------------------------------------------------------

                         3850      3860      3870      3880      3890      3900      3910      3920
                ....|....|....|....|....|....|....|....|....|....|....|....|....|....|....|....|
Ts pathogen_TSM AGCCGGTTGGCCGGCCTCCGGGCCGCGCCAACAGCCCCCAAGCCCTCTGTTTGCAGAATACATATTCTACCCCAGTACTA
Ta TASK.contig.  --------------------------------------------------------------------------------

                         3930      3940      3950      3960      3970      3980      3990      4000
                ....|....|....|....|....|....|....|....|....|....|....|....|....|....|....|....|
Ts pathogen_TSM TGCCTTCTTTTCCCTTAAAGGGAGCCTCGGGACGCTACAATCTTATTAGCAGGAACTTTGACATGGTTTCTAATATTCAG
Ta TASK.contig.  --------------------------------------------ATCAGCAGGAACTTTAACATGCTTTTTAATCTCCAG

                         4010      4020      4030      4040      4050      4060      4070      4080
                ....|....|....|....|....|....|....|....|....|....|....|....|....|....|....|....|
Ts pathogen_TSM AGAAATCGTGCACGACTTTTTAAACAATGCTATTAATAC-TTTTAAATTAAATTTTCCTGACTTACCGTATTACCTTCCT
Ta TASK.contig.  AAAAATCATGCACAAGTTTTTAAACAATGCTATTATTACCTTTTAAATGAAATTTTCCTGACTTACCGTATTAACTTCCT

                         4090      4100      4110      4120      4130      4140      4150      4160
                ....|....|....|....|....|....|....|....|....|....|....|....|....|....|....|....|
Ts pathogen_TSM CCTCGTAGTCGCCGTGCTAAAAGCAGAGAAAGTTAGGTGAACAAATAGAACTCATTGTGCAGTTTGTGTTGGAAAGACAG
Ta TASK.contig.  CCTCGTAGTCGTCGTGCTAAAAGCAGAGAAAGTTGGGTGAACAAATAGAACTCATTGTGCAGTTTGTGTTGAAAAGACAG

                         4170      4180      4190      4200      4210      4220      4230      4240
                ....|....|....|....|....|....|....|....|....|....|....|....|....|....|....|....|
Ts pathogen_TSM CTACCTTTTGTGATTTGACAAGTTGCCAAGACTCTCAAGATCGAAAACTCACTTCGCCTTATCCTACGAGGGAGGATATT
Ta TASK.contig.  CTACCTTTTGTGATCCTACAAGTTGCTAAAACTGTCAAGATCAAAAGCTCATTTCTCCTCATCCTACGAGGGAGGATGTT

                         4250      4260      4270      4280      4290      4300      4310      4320
                ....|....|....|....|....|....|....|....|....|....|....|....|....|....|....|....|
Ts pathogen_TSM ATTATTTAACGAAGAACAAAAACGTCTTCATTAAATGTTAAAAGGAAATGGACACCAAATAAAACGTAGAGAAAGAAGCA
Ta TASK.contig.  ATTGTTTAACGAAGAACAAAAACGTCTTCATCAAATGTTAAGAGAAAATGAACGCCAAATAAAACCTAGAGAAAGAAGTA

                         4330      4340      4350      4360      4370      4380      4390      4400
                ....|....|....|....|....|....|....|....|....|....|....|....|....|....|....|....|
Ts pathogen_TSM TATTTTGTGACTGTGTTTGTCTACGCCCTTGTTTATTCAACCCCATAGCAACGGCAGGGTCGTTGGATGAAGGATCAATT
Ta TASK.contig.  CATTTTATGACTTTGATTGTCTACGCCCTTGTTTATTCAACGCCATAGCAATGGTAGGGTAGTTGGATGAGAGATCAATT

                         4410      4420      4430      4440      4450      4460      4470      4480
                ....|....|....|....|....|....|....|....|....|....|....|....|....|....|....|....|
Ts pathogen_TSM CTTGACGTAGCGGCTGCTACCGAGCCGCTACTTAGTGTTCCCCTCTTACCGTCGGTTGTTGCACTTCACATGTTTGTGCC
Ta TASK.contig.  CTTGACGTGGCAGCTGCTACCGAGCCGCTACTTAGTGTTCCCTTTTAACCGCCGGTTGTTGCACTTCACATGTTTGTGCC

                         4490      4500      4510      4520      4530      4540      4550      4560
                ....|....|....|....|....|....|....|....|....|....|....|....|....|....|....|....|
Ts pathogen_TSM ATTACCATAGAGCCCCCCCCCGCATTCCACCAACAATGCCGCATGTGCTTTCAAAGGCCCCTATTTTGCTCTGGTAGCTT
Ta TASK.contig.  ATTAACACAGAGCCTCCT---GCATACCACTAACAATGCCGCATGTGCTTTCAAAGGCCCGTATTTTGCTCTGCTAGCTT

                         4570      4580      4590      4600      4610      4620      4630      4640
                ....|....|....|....|....|....|....|....|....|....|....|....|....|....|....|....|
Ts pathogen_TSM TACCCTAGGATCGTGCCTTATGATTAATTGGGTACCAACATTTGTCAGTTGTGGGTAATTTAGAGATCTTTGAGATAATC
Ta TASK.contig.  TACCATAGGATTATGCCTTATGGTTAATTGGGTACCAACATTTGTCATTTATGGGTAATTTAGAGATCTTTGAGATAATC

                         4650      4660      4670      4680      4690      4700      4710      4720
                ....|....|....|....|....|....|....|....|....|....|....|....|....|....|....|....|
Ts pathogen_TSM CTCCCGTAGCTATTGCCCCAGAATAAATGCTTCTGAGCGTTGTCAAATAGTTCTATCAAGCCTTGTCAGATGGATGGATT
Ta TASK.contig.  CCCCCATAGCTATTGCCTCAGAATGAATACTTCGGAGCGTTGTCAAATAATTCTATCAAGCCTTGTCAAATGGATGGATT
```

```
                            4730      4740      4750      4760      4770      4780      4790      4800
                    ....|....|....|....|....|....|....|....|....|....|....|....|....|....|....|....|
Ts pathogen_TSM     CGAGGTGAACAGATTAAAGGTGGTAGAGTCCAAATGAGTAGTTCAGGGAGGATTACATAAATAAGCGATGGACAGGCCCT
Ta TASK.contig.     TGGAATAAATAGATTGAAGTTGGTAGAATCTAAATGACTAGGTAAGGGAGGATTACATAACTAAGCGATGGCAACCCCT

                            4810      4820      4830      4840      4850      4860      4870      4880
                    ....|....|....|....|....|....|....|....|....|....|....|....|....|....|....|....|
Ts pathogen_TSM     TGGTTAACATAGGGATGGACAGGTCCAGGAAAGGGTGTGTACACCAAGGACAAATAACTCGGCATTATACGGACACAGTA
Ta TASK.contig.     TGGTTAAAATTGGGGCTGGCGGGTCCAGGAAAGGGCGTGTACACCAAGAACAAATAACTCTGCAATATACGGACACAGTA

                            4890      4900      4910      4920      4930      4940      4950      4960
                    ....|....|....|....|....|....|....|....|....|....|....|....|....|....|....|....|
Ts pathogen_TSM     CATCAGTAGATTGATGGATTCCCAGTAGATTGATGGACAGATTTAAGATAAGTAGACGGAAGGAGAAAGAGCAGTGAAAT
Ta TASK.contig.     CATCAGTAGATTGACAGATTCCCAGTAGATTGATGGACAGATTTAAGATGAGTAGACGGAAGGAGAAAGAGCAGTGAAAT

                            4970      4980      4990      5000      5010      5020      5030      5040
                    ....|....|....|....|....|....|....|....|....|....|....|....|....|....|....|....|
Ts pathogen_TSM     GATGTCCTATGCCACTATATTGCAGGAGTTAGTGCAACTTATTGTCAAAAACATTAGCAAATAAGGTTATTGGGTTGTTC
Ta TASK.contig.     GATGGCCTAAACCACTATATTACAGGAGTTAGCGCAACTTATTATCAA--ACATTAGCAAATAACGTTATTGGGTTGTTC

                            5050      5060      5070      5080      5090      5100      5110      5120
                    ....|....|....|....|....|....|....|....|....|....|....|....|....|....|....|....|
Ts pathogen_TSM     AAATGTGTTTCAACTGCTGTGTATTTAATACTCTAACTGGTGGCCCAATCTCTGGTCCAGTCTGGAGCCTTCCAGGTCAC
Ta TASK.contig.     AAATGTGCTTCAGCTGCTGTGTATTTAATGCTCGACCTGGTGGCCCAATCTCTGGTCCAGTCTGGAGCCTTCGGGGTCAC

                            5130      5140      5150      5160      5170      5180      5190      5200
                    ....|....|....|....|....|....|....|....|....|....|....|....|....|....|....|....|
Ts pathogen_TSM     TGAGATACGAGCGATAGCAATCCGCTCTTTCTTCTTTGTCGAGTGCTGCGGTTAAAATGATTTCTCCTTTCTAGCAGCCC
Ta TASK.contig.     TGAGGTACAAGCGATAGCAATCCGCT----CTTCTTTATCGGGTACTGCCGTTGAAATGATTTTTCCTTTCTAGCAGCCT

                            5210      5220      5230      5240      5250      5260      5270      5280
                    ....|....|....|....|....|....|....|....|....|....|....|....|....|....|....|....|
Ts pathogen_TSM     GTTTAAAGTGGTTCTAATAAGCAGAAGACCGAATGATAAATTGCAAG-------------------TTTCGGTAGGCAGA
Ta TASK.contig.     GTTAAAGGTAGTTCTAACAAGCAGAAGACCGTATAATGAATTGCAAGGGGTTTCATTTGGTTCAAGTTTCAGTAGGCAGA

                            5290      5300      5310      5320      5330      5340      5350      5360
                    ....|....|....|....|....|....|....|....|....|....|....|....|....|....|....|....|
Ts pathogen_TSM     CGGCAATAGAGTGAGAGAGGAAGGGAGAAGAATTTCGGACAATTTTTGATTAAAAAGTGCCAAAACTTGAACAAATGCCA
Ta TASK.contig.     CAGCAATAGAACGAGAGAGGAAGGTAGAAGAATTTCGAACAATTTTTGATTAAAAAGTGCCAAAACTTAAACAAATGCCA

                            5370      5380      5390      5400      5410      5420      5430      5440
                    ....|....|....|....|....|....|....|....|....|....|....|....|....|....|....|....|
Ts pathogen_TSM     TTCTTTTAGGCGAAGTGAAGTAATGAAATGTCAGAATGACTTACAATACCACTTATCAGTGGAAAGAATTTGCAAGGCAA
Ta TASK.contig.     TTCCTTTAGGCGAAGTGAAGTAATGAAATGTCAGAATGACTTACAATACCACTTATCAATGGAAAGAATTTGCAAGGCAA

                            5450      5460      5470      5480      5490      5500      5510      5520
                    ....|....|....|....|....|....|....|....|....|....|....|....|....|....|....|....|
Ts pathogen_TSM     TGCGAACTGTTAGGCAACTAAAAGAGTGAATCTGGAAGGTGAAGGAGAAACGGTACGCAACGATTTTGTGTCAGTAAGTA
Ta TASK.contig.     TGCAAAATGTTTGGCAACTAAAAGAGTGAATCTGGAAGGTGAAGGAGAAACGATACGCAGCGATTTTGTGTCAGTAAGTA

                            5530      5540      5550      5560      5570      5580      5590      5600
                    ....|....|....|....|....|....|....|....|....|....|....|....|....|....|....|....|
Ts pathogen_TSM     ATTAAGTATCAGTCAGTAATTGTGGCTTGGCAAAATGCGATTCCTCTTTTGATTGTAAATTGCACGCCACGTCTCTTCTC
Ta TASK.contig.     ATTA----TCAGTCAGTAACTGTGGCTTCGTAAAATGTGATTCCTCTGTTGATTGTAAATTGCACACCATGTCTATTCAC

                            5610      5620      5630      5640      5650      5660      5670      5680
                    ....|....|....|....|....|....|....|....|....|....|....|....|....|....|....|....|
Ts pathogen_TSM     TCCACTTGGATTGGATATTAGGCCTTTCCCACATCCTTCATTTCACTTTCCAAGGCAGCATTGGGAGTTAGGACTTAGTC
Ta TASK.contig.     TCCACTTGGGTTGGATTATAGGCCTTTCCCACATCCTTCATTTCACTTGCCAAGACAGCATCATGAGTTAGGAATTAGTC

                            5690      5700      5710      5720      5730      5740      5750      5760
                    ....|....|....|....|....|....|....|....|....|....|....|....|....|....|....|....|
Ts pathogen_TSM     ACACCCGATCGTGTTATACTGTGGGGCCTATCCAGCTATTCTTGTATTGAATCCTCGTATAATTTTACCACAGGAGTTTC
Ta TASK.contig.     ACACCCAATCGTGCTATACTGCGGGGTCTATCCAGCTATTCCCCTTTTAAATCTACGTATAATTTTACCACAGGAATTGG

                            5770      5780      5790      5800      5810      5820      5830      5840
                    ....|....|....|....|....|....|....|....|....|....|....|....|....|....|....|....|
Ts pathogen_TSM     CATTTCTGGTAGAGGAGGGGAGTGGCTTGAAATATTCAAAAAGCCTTTGCTCTATGCCGACCGTTCAACCAGCTGCAGCC
Ta TASK.contig.     CATTTCTGGAAGAGGAGGGGAGTGACTTGAAGTATTCAAAAAGCCTTTGCTCTATGCCGACCGTTCAACCAGCTGCAGTC

                            5850      5860      5870      5880      5890      5900      5910      5920
                    ....|....|....|....|....|....|....|....|....|....|....|....|....|....|....|....|
Ts pathogen_TSM     AAAGCCAAGGAGTGGCAGGAGTGAACCAAGAGGCAGACTCAAAACACCAAATGGGTGGGGACTAAAGCGATTGCAGAGTG
Ta TASK.contig.     AAGACCAAGGAGTGGTAGGAGTGAATCCAGAAGCAGACGCACAACACCAAAAGGGTGGAGACTAAAGCGATTGCAGAGAG
```

```
                     5930      5940      5950      5960      5970      5980      5990      6000
                 ....|....|....|....|....|....|....|....|....|....|....|....|....|....|....|....|
Ts pathogen_TSM  TTTGGCCATGATTTTTGACCCTACTCACGTCTCTAACATGCTAGTCCCCGAGTTGGACGTGGTTCAGGCGTCTCAGCAGC
Ta TASK.contig.  TATGGCTATGATTCTTGACCCTACTCACGTCTCTAACATGCTAGTCTCCGAATTGGACGTGGTTCAGGCCTCTCAGCAGC

                     6010      6020      6030      6040      6050      6060      6070      6080
                 ....|....|....|....|....|....|....|....|....|....|....|....|....|....|....|....|
Ts pathogen_TSM  TGCCAGCAAATGCGCATGAAGGAGTGCATTT-CACTCAA----------------------CGGTTTGGCTTCCTTTGTG
Ta TASK.contig.  TGCCAGCAAGTGCTCATGAAGGAGTGCTTTTTCACTCAATAGGCCACATTCTATTCTGAAGCGGTTTGGCTTCCTTTCTT

                     6090      6100      6110      6120      6130      6140      6150      6160
                 ....|....|....|....|....|....|....|....|....|....|....|....|....|....|....|....|
Ts pathogen_TSM  ATTTCCTGTTTTACCGGACAAAGCACCCAACTAATATCGAATGACACGGAGGGGCCTTAGGAGGTAGTAAGAAGAAAGAA
Ta TASK.contig.  GTTTCCCGTTTCACCGGACAAAGCATCCAACTAATATCGAATGACACGGAGGTGCCTTAGGAGGTAGTAAGAAGAAAGAA

                     6170      6180      6190      6200      6210      6220      6230      6240
                 ....|....|....|....|....|....|....|....|....|....|....|....|....|....|....|....|
Ts pathogen_TSM  CCCTTTTAGTGGAGTGATTGTCCGACAGAA--TTGGTGTGATTTCGGTGTGGTTATAATGATTGGTGGTAAGCAGTCCTC
Ta TASK.contig.  CGACTTTAATGAAGTGAGTGTCCGACAAAAAATTGGTGTGATTTCGGTGTGGTTATAATGATCGGTGGTAAGTAGTCCTC

                     6250      6260      6270      6280      6290      6300      6310      6320
                 ....|....|....|....|....|....|....|....|....|....|....|....|....|....|....|....|
Ts pathogen_TSM  TCACCGCTCTCCCGGTGCTCCGTTTTATCTTCATCCACCTGCTTCTTACGCATGAGTGTGACCCCACCTCATCCGCTTTC
Ta TASK.contig.  TCACCGCTCTCCCGGTGCTCCGTTTTATCTTCATCCACTTCATTATTACACATGAGTGTGACCTCACCTCATCCGCTTTC

                     6330      6340      6350      6360      6370      6380      6390      6400
                 ....|....|....|....|....|....|....|....|....|....|....|....|....|....|....|....|
Ts pathogen_TSM  AAGTCTGTGCAAGTTTTTT-GTACAGTATGTTGCCTAGCTTTCGGCGGAATCACAGCATCAGGACTTACACCTCTGTGGT
Ta TASK.contig.  AAGTCTGCGCAAGTTTTTTTGTACAGTATGTTGCCTAGCTTTCGGTGAAACCACAGCATCAGGACTTACACCTCTGTGGT

                     6410      6420      6430      6440      6450      6460      6470      6480
                 ....|....|....|....|....|....|....|....|....|....|....|....|....|....|....|....|
Ts pathogen_TSM  CCAGAAAAGCGTGATGCACGGGATCCCTCTCCTCTCTCCCTCTTGACCCCATACTTGACGTAGAGGCTCAATGCATCGCT
Ta TASK.contig.  CCAGAAAAGTGTGATGCACGGAATCCCTCTCCTCTCTCCCTCTTCACTCCATACTTGGCGTAGAGGCTCAATGCATCGCT

                     6490      6500      6510      6520      6530      6540      6550      6560
                 ....|....|....|....|....|....|....|....|....|....|....|....|....|....|....|....|
Ts pathogen_TSM  TCGTAAAGCCAACAACCTCGGGCCCCTTTCGTTGCGTCTATCGCCATTTTTTACCATGAGGGTCAGGTTCAGTGCAGTC
Ta TASK.contig.  TCGTAAAGCCAACAACCTCGAGCCCCTTTCTTTGCGTCCATTGCCATTTTTTACCATGAGGAGTCAGGTTCGGTGCAGAC

                     6570      6580      6590      6600      6610      6620      6630      6640
                 ....|....|....|....|....|....|....|....|....|....|....|....|....|....|....|....|
Ts pathogen_TSM  TGGTAGAAAGTACAGACAAATGTGCCACATCGAGGGCAGACTAGCCCCCTCCCCCTACCTCTCTGTACTTTACTCTTTTA
Ta TASK.contig.  TGGTAGAAAGTACAGACAAATGTGTCACATGGAGGGCAGACTAGCCCCCTCGCCCTACCTATCTGTACTTTACTCTTTTA

                     6650      6660      6670      6680      6690      6700      6710      6720
                 ....|....|....|....|....|....|....|....|....|....|....|....|....|....|....|....|
Ts pathogen_TSM  ATGAACAGTTGCATGCATTCCCCAGCATCTAGTGGAGTCACGCGGACAGCTGCACCAGATCTGCGGCCTCCCCACGTGGT
Ta TASK.contig.  ATGAACAGTCGCATGCATTCCGCAGCATCGCGTGGAGTCACGCGGATAACTGCACCAGCTTTGCGGCCTTCCCACGTGGT

                     6730      6740      6750      6760      6770      6780      6790      6800
                 ....|....|....|....|....|....|....|....|....|....|....|....|....|....|....|....|
Ts pathogen_TSM  TCGGTGCAGCCATAGGTAAAATGAATTATTTTCTCCTTAAAATTTTCCCGTAAAACTTCCCATCCTCTTCACAGACATCT
Ta TASK.contig.  TCGGTGCAAATATAGGTAAAAGAAATTATTCTTTCCTTAAAATTTTCCCGTTAAATTTCCTATCCTCTTCAAAGATATCT

                     6810      6820      6830      6840      6850      6860      6870      6880
                 ....|....|....|....|....|....|....|....|....|....|....|....|....|....|....|....|
Ts pathogen_TSM  TCAAGCAGATCGCCTGGCGCCAGAAGACCTTCCTCTCCTAGCAACTTTTGATACAAGAACAAAGTGACAGCGCCAGACC
Ta TASK.contig.  TCAAGCAGATCGCCTGGACGTCGGAAGACCTTCCTCCCCTGGCAGCTTTTGATATAAGAACAAAGTGACAGCACCAGACC

                     6890      6900      6910      6920      6930      6940      6950      6960
                 ....|....|....|....|....|....|....|....|....|....|....|....|....|....|....|....|
Ts pathogen_TSM  AAGTTGTGAGGTTCTTGAACGTTGTTAATGGCCTCCAAGAAGAAATATGTACTTCCACTGCTCTACCGTTCGGCTATGTA
Ta TASK.contig.  AAGTTGTGAGATTCTTGAACGCTGTTAATGGCCTCCAAGAAGCAGTATGTACTGCCACTGCTCTACCGTTTGGCTATTTA

                     6970      6980      6990      7000      7010      7020      7030      7040
                 ....|....|....|....|....|....|....|....|....|....|....|....|....|....|....|....|
Ts pathogen_TSM  TCAAATGCGTGTCTGTGACATGTATGCGTGATGTCGGCACAGCACGAATGCGTGACATGTCACGATAACACCAACCACCT
Ta TASK.contig.  TCAAATGCGTGTCTGTGACACGTATGCGTGACGTCGGCACGACACGAATGCGTGACATTTCACGATACCACCAACCACCT

                     7050      7060      7070      7080      7090      7100
                 ....|....|....|....|....|....|....|....|....|....|....|....
Ts pathogen_TSM  CCACCAAGTCGCGATAAGGACCTCGTGATTGTGCGTACTTGAGAAATTAGCTTGACGGAACAGA
Ta TASK.contig.  CCACCAAGTCACGATAAGGACCTCGTGATTGTGTGTACTTGAGAAATTAGCTTAACGGAACAGA
```

Positions in assembly:

T solium pathogen_TSM_contig_00641 29000 to 36000
T asiatica TASK.contig.00624.50118 32169 to 38087

Red: TSD
Blue: TRIM

Supplementary Data 7. Estimated age of insertion of *ta-TRIM* elements. Histogram showing the age of insertion of *ta-TRIM* elements as estimated from the divergence between 5´ and 3´ LTRs (see the main text for details).

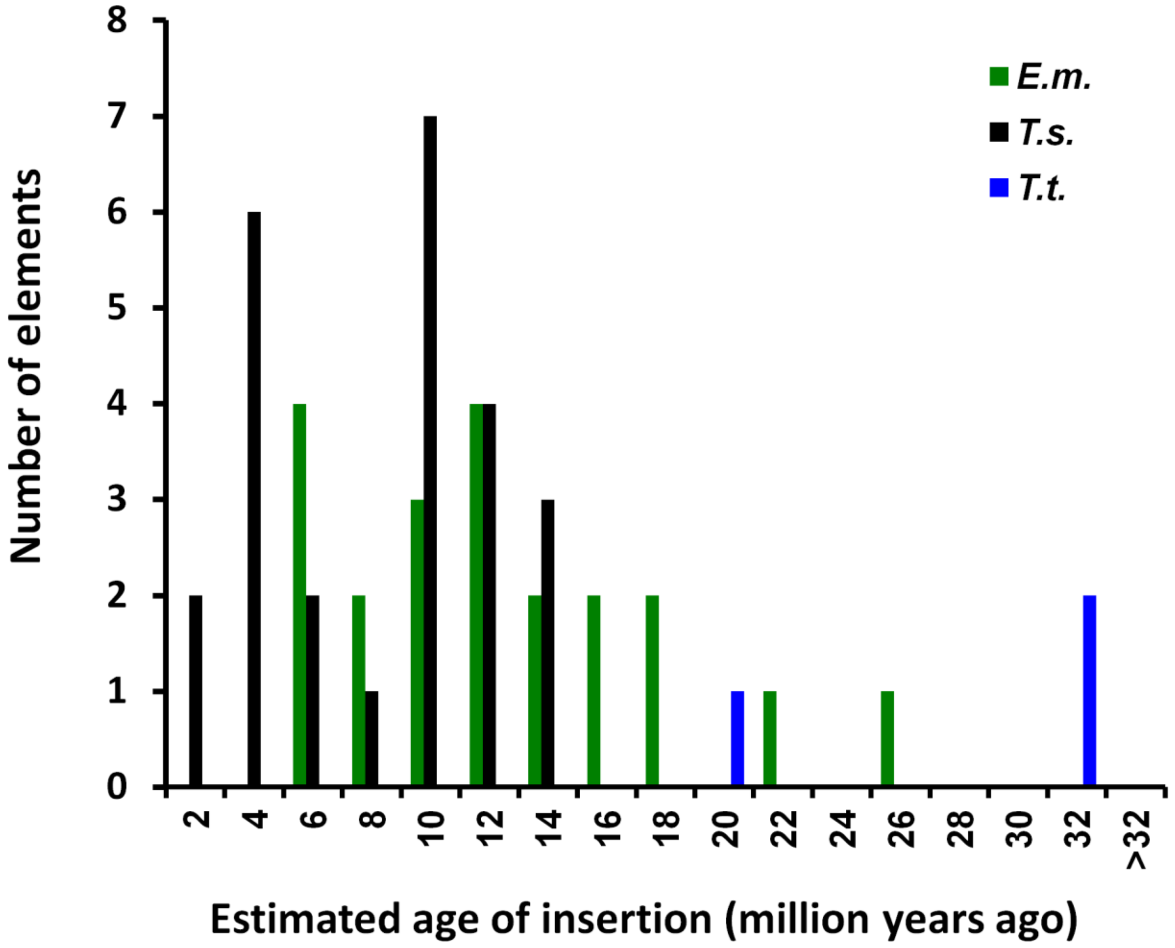

Supplementary Data 9. Transcriptional fusion of an LTR with a downstream gene in *E. granulosus*.

One EST (CN650872) indicates an alternative isoform for gene EgrG_00080500, which begins with transcription from a solo-LTR and is spliced with internal exons of that gene. The predicted main splicing isoform is show with white bars. The *E. multilocularis* ortholog (EmuJ_000805000) also has a solo-LTR upstream of the gene but no EST evidence was found for transcriptional fusions in that case.

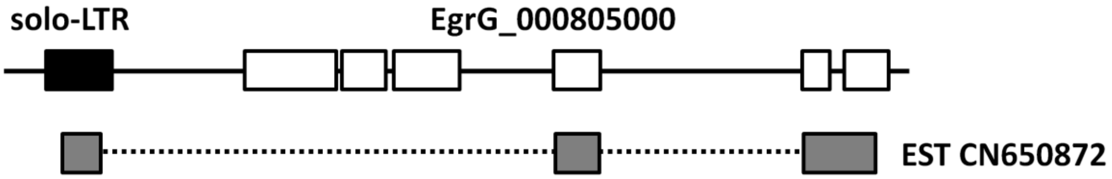

Supplementary Data 10. Summary of the life cycle of *Echinococcus multilocularis*.

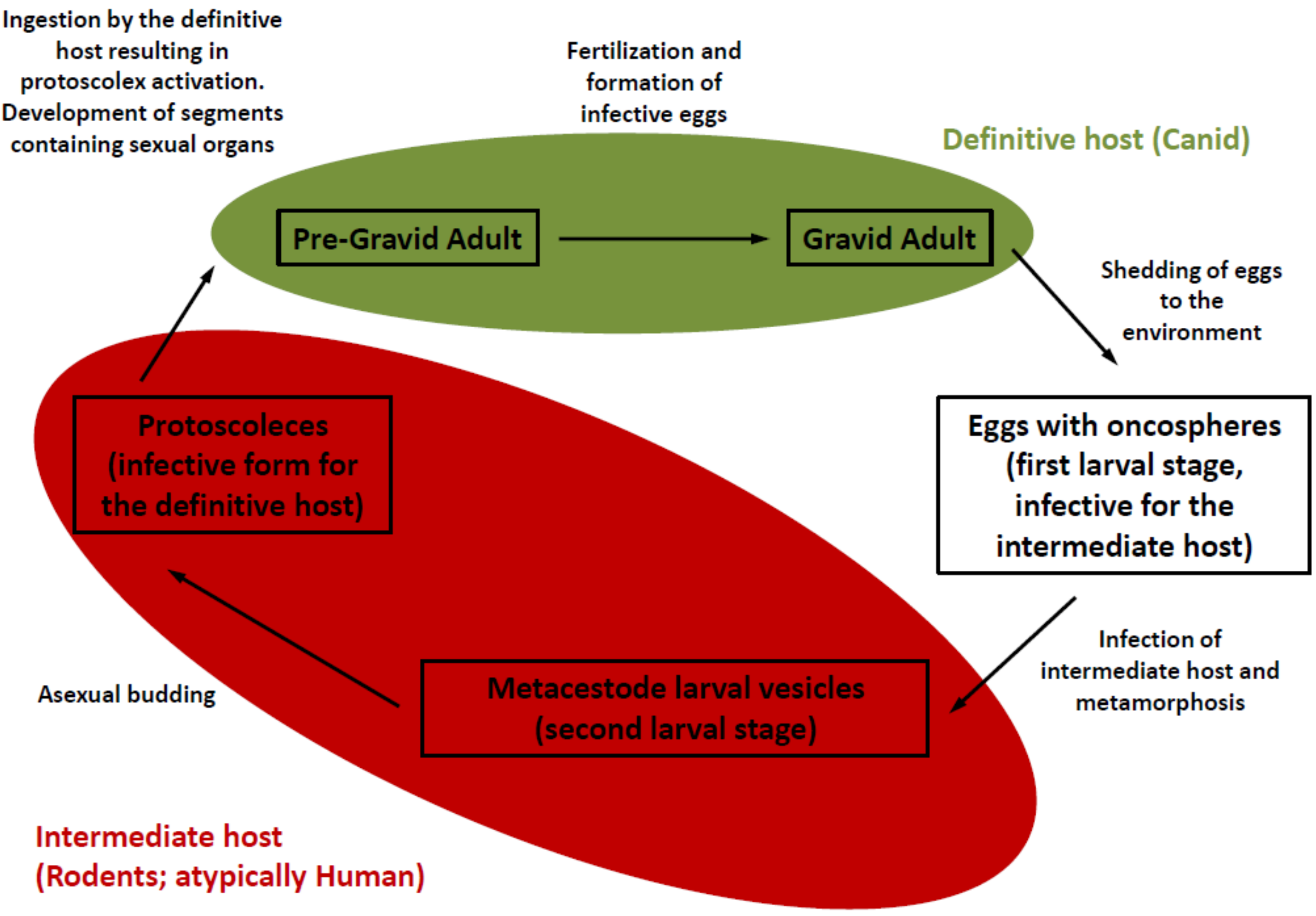

Supplementary Data 12. Heat-map showing the expression of full-length *ta-TRIM*s (counts per million reads) in different stages of *E. multilocularis* development (from Supplementary Data 11). (PC: primary cells; MV, metacestode vesicles; NAP, non-activated protoscoleces; AP, activated protoscoleces; PGA, pre-gravid adults; GA, gravid adults).

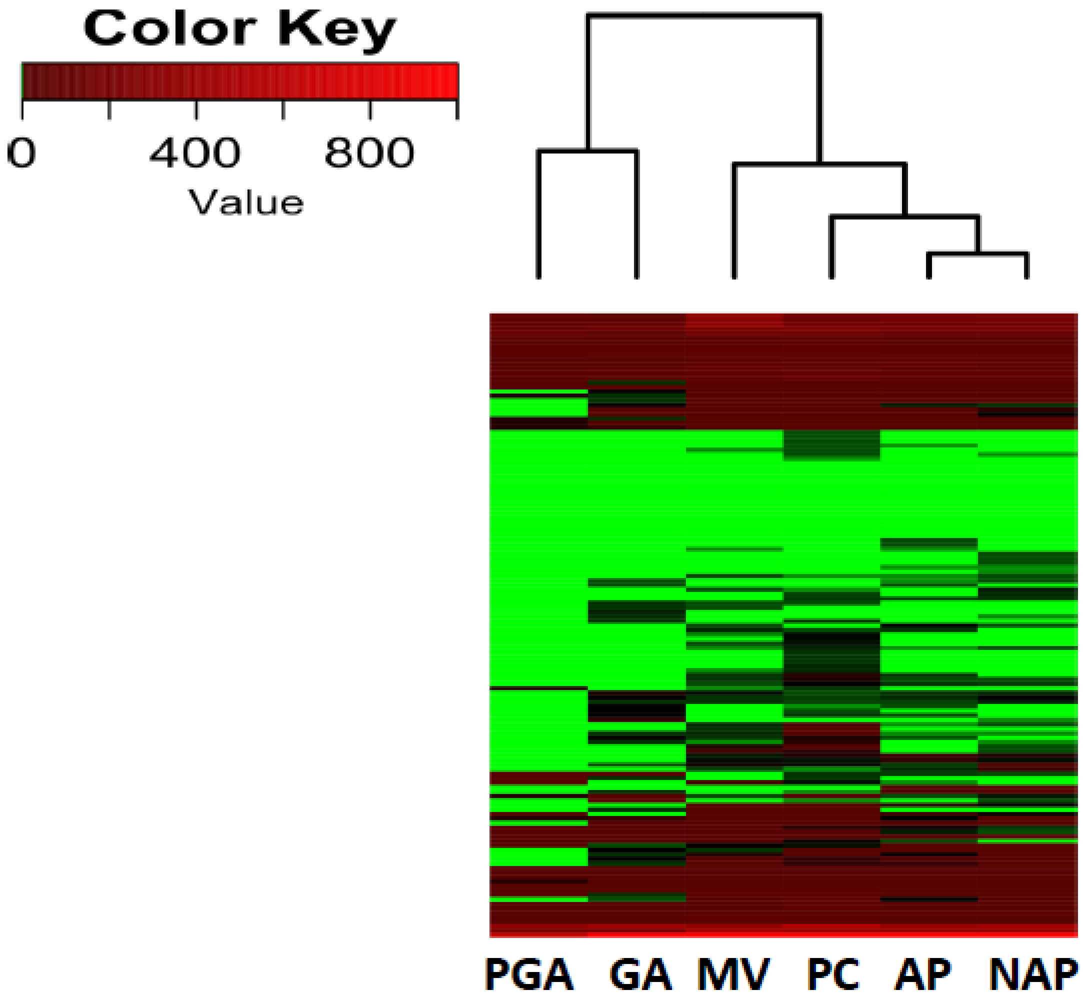

Supplementary Data 13. RNAseq evidence for expression of partial *ta-TRIMs*. Histogram showing the distribution of expression levels of individual partial *ta-TRIM*s of *E. multilocularis* (average reads per dataset, normalized by the total number of uniquely mapping reads times 10^6 (RPM)). **B.** Expression of representative individual solo-LTRs elements across datasets (PC: primary cells; MV, metacestode vesicles; NAP, non-activated protoscoleces; AP, activated protoscoleces; PGA, pre-gravid adults; GA, gravid adults). For the selection of the representative elements, all solo-LTRs were divided into four bins according to their RPM values (1-4), and the solo-LTRs with the median RPM value for each bin was selected and graphed.

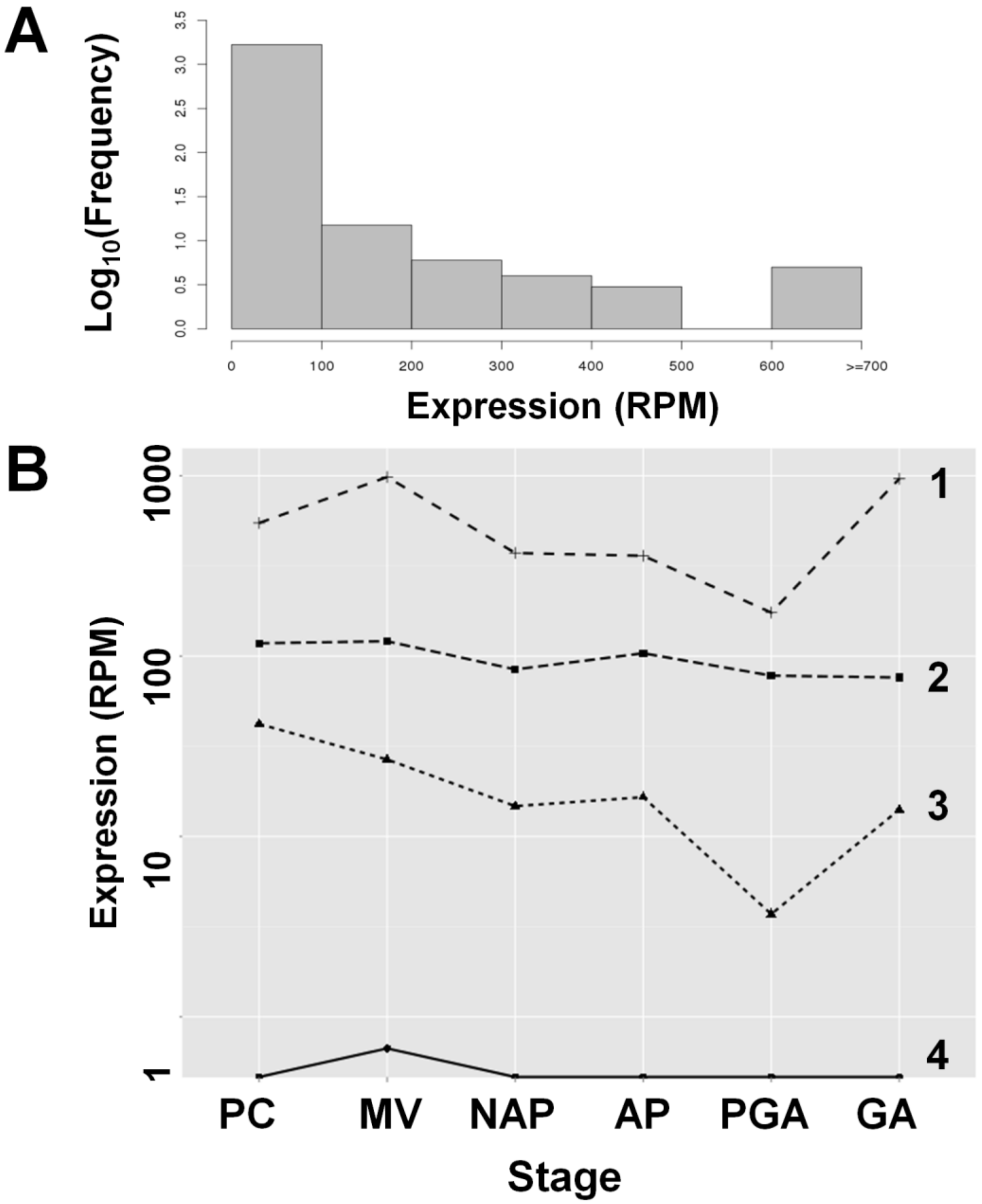

Supplement: Supplementary Data [file supp_evv126_suppl_data.zip › Supplementary_Data_Together_S1_S5_S6_S7_S9_S10_S12_S13.pdf]
