# Supplementary material for: A Novel Terminal-Repeat Retrotransposon in Miniature (TRIM) Is Massively Expressed in Echinococcus multilocularis Stem Cells
Source: Genome Biol Evol. 2015 Jul 1;7(8):2136–53. doi: 10.1093/gbe/evv126 (PMC4558846; doi:10.1093/gbe/evv126)
Supplement: Supplementary Data [file supp_evv126_suppl_data.zip › New_Microsoft_Office_Word_Document.docx]

**Supplementary data**

**Supplementary Data 1. Alignment of examples of *ta-TRIM* elements from taeniids.**

**Supplementary Data 2. GFF files and table of *ta-TRIM*s and *lennies* elements of *E. multilocularis.*** Gff files are to be used with version 4 of the *E. multilocularis* genome from www.genedb.org

**Supplementary Data 3. GFF files and table of *ta-TRIM*s and *lennie* elements of *E. granulosus*.** Gff files are to be used with version 3 of the *E. granulosus* genome from www.genedb.org

**Supplementary Data 4. GFF files of *ta-TRIM*s of *T. solium.*** Gff files are to be used with the *T. solium* genome from www.genedb.org

**Supplementary Data 5. Distribution of *ta-TRIM*s in the genome of *E. multilocularis.*.** The figure displays graphically the distribution of *ta-TRIM*s within the chromosomes of *E. multilocularis*. The number of sequences showing similarity to the LTR region of *ta-TRIM*s of *E. multilocularis*, obtained through a combination of blast and HMM analyses, was counted and graphed for non-sliding windows of 100 kb in each chromosome. Total lengths (X-axis) for chromosomes 1 to 9 are 20116480, 17597483, 14622996, 13762453, 11859843, 7212196, 7437390, 6203120 and 4267854 bp, respectively.**Supplementary Data 6. Examples of integrations of *ta-TRIM*s after the divergence of *Echinococcus* spp., and of *Taenia* spp.** Alignment of a *ta-TRIM* element and its surrounding region from *E. multilocularis* with the ortholog region of *E. granulosus*, and of a *ta-TRIM* element and its surrounding region from *T. solium* with the ortholog region of *T. asiatica.*

**Supplementary Data 7. Estimated age of insertion of *ta-TRIM* elements.** Histogram showing the age of insertion of *ta-TRIM* elements as estimated from the divergence between 5´ and 3´ LTRs (see the main text for details).**Supplementary Data 8. List of *E. multilocularis* loci with similarity to *ta-TRIM*s and with evidence of transcription from ESTs and 3’ RACE analyses.** Locus number and the position of ESTs and 3’ RACE sequences (mapped by BLASTN analyses) are indicated for versions 3 and 4 of the *E. multilocularis* genome, as well as details of the manual annotation of the loci. Note that one EST may have several discontinuous blast hits due to the presence of introns.

**Supplementary Data 9. Transcriptional fusion of an LTR with a downstream gene in *E. granulosus*.** One EST (CN650872) indicates an alternative isoform for gene EgrG_00080500, which begins with transcription from a solo-LTR and is spliced with internal exons of that gene. The predicted main splicing isoform is show with white bars. The *E. multilocularis* ortholog (EmuJ_000805000) also has a solo-LTR upstream of the gene but no EST evidence was found for transcriptional fusions in that case.

**Supplementary Data 10. Summary of the life cycle of *Echinococcus multilocularis*.**

**Supplementary Data 11. Table of RNA-Seq analysis for individual *ta-TRIM*s of *E. multilocularis*.** The number of reads for each element was normalized by the total number of uniquely mapping reads from each dataset. No normalization was attempted by sequence length since the proportion of each element that can be uniquely mapped varies. This should give only a small impact given the sequence length similarity across all elements of one kind.

**Supplementary Data 12**. **Heat-map showing the expression of full-length *ta-TRIM*s (counts per million reads) in different stages of *E. multilocularis* development** (data from Supplementary Data 11).

**Supplementary Data 13. RNA-Seq evidence for expression of partial *ta-TRIMs*.** Histogram showing the distribution of expression levels of individual partial *ta-TRIM*s of *E. multilocularis* (average reads per dataset, normalized by the total number of uniquely mapping reads (RPM)). **B.** Expression of representative individual solo-LTRs elements across datasets (PC: primary cells; MV, metacestode vesicles; NAP, non-activated protoscoleces; AP, activated protoscoleces; PGA, pre-gravid adults; GA, gravid adults). For the selection of the representative elements, all solo-LTRs were divided into four bins according to their RPM values (1-4), and the solo-LTRs with the median RPM value for each bin was selected and graphed.

**Supplementary Data 14. Fasta files of *ta-TRIM*s and *lennie* elements of *E. multilocularis*, *E. granulosus* and *T. solium*.**
